# Low oxygen but dynamic marine redox conditions permitted the Cambrian Radiation

**DOI:** 10.1126/sciadv.ads2846

**Published:** 2025-01-24

**Authors:** Ruaridh D. Alexander, Andrey Yu. Zhuravlev, Fred T. Bowyer, Laetitia Pichevin, Simon W. Poulton, Artem Kouchinsky, Rachel Wood

**Affiliations:** ^1^ School of GeoSciences, University of Edinburgh, James Hutton Road, Edinburgh EH9 3FE, UK.; ^2^ Borissiak Palaeontological Institute, Russian Academy of Sciences, Profsoyuznaya Street 123, Moscow 117647, Russia.; ^3^ School of Earth and Environment, University of Leeds, Leeds LS2 9JT, UK.; ^4^ Department of Palaeobiology, Swedish Museum of Natural History, Box 50007, Stockholm SE-10405, Sweden.

## Abstract

Whether metazoan diversification during the Cambrian Radiation was driven by increased marine oxygenation remains highly debated. Repeated global oceanic oxygenation events have been inferred during this interval, but the degree of shallow marine oxygenation and its relationship to biodiversification and clade appearance remain uncertain. To resolve this, we interrogate an interval from ~527 to 519 Ma, encompassing multiple proposed global oceanic oxygenation events. We integrate the spatial and temporal distribution of shallow water, in situ reef metazoans, and trilobites, with high-resolution multi-proxy redox data through the highly biodiverse Siberian Platform. We document primarily dysoxic water column conditions, suggesting that early Cambrian metazoans, including motile skeletal benthos, had low oxygen demands. We further document oxygenation events coincident with positive carbon isotope excursions that led to modestly elevated oxygen levels. These events correspond to regional increases in species richness and habitat expansion of mainly endemic species, offering a potentially globally applicable model for biodiversification during the Cambrian Radiation.

## Teaser

The Cambrian Radiation of animals occurred under dynamic but low marine oxygen conditions.

## INTRODUCTION

**Fig. 1.**
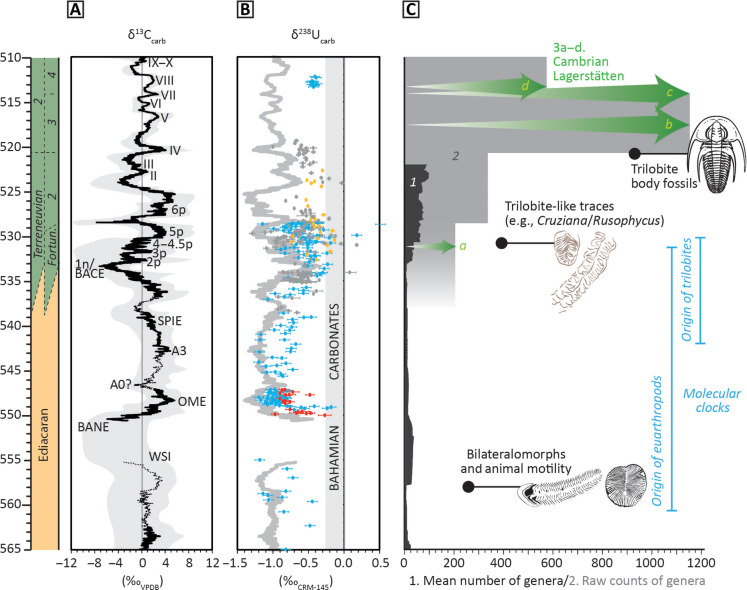
Carbon and redox cycles with reconstructed metazoan biodiversification. (**A**) Global composite δ^13^C_carb_ curve with uncertainty. (**B**) Temporally calibrated carbonate U isotope (δ^238^U_carb_) data from multiple sources. Data are color-coded on the basis of region (blue, South China; gray, Siberia; red, Kalahari craton; yellow, Morocco). The oscillating pale gray line shows an inverted representation of the 10-point moving average of global δ^13^C_carb_ data (A), used as a visual aid to show how a perfect anticorrelation between δ^13^C_carb_ and the most reliable δ^238^U_carb_ data would appear. (**C**) Reconstructed skeletal metazoan generic richness [1; from (*12*)] and frequency of genera per Cambrian stages [2; (*161*)]; origin of euarthropods (*162*) and trilobites (*26*) inferred from molecular clocks; first appearance of bilateralomorphs, trilobite-like trace fossils (*12*), and trilobite body fossils (*26*). Example Cambrian Lagerstätten: a, Kuanchuanpu; b, Chengjiang; c, Sirius Passet; d, Sinsk and Emu Bay Shale. Modified from (*12*).

The Ediacaran to Cambrian rise of animals (metazoans) was a fundamental event in the history of life, expressed by the canonical Cambrian Radiation [~539 to 515 million years ago (Ma)], which records the apparently rapid appearance and diversification of modern metazoan body plans and ecologies (Fig. 1C) (*1*). A causal relationship between increasing shallow marine oxygenation and the Cambrian Radiation has long been proposed (*2*), but it is not clear whether oxygen availability rose progressively through this interval (*3*, *4*) and, if so, how this promoted biotic innovation and the radiation of early metazoans.

Models for the early Cambrian suggest atmospheric oxygen concentration of ~5 to 10% [~24 to 48% of the present atmospheric level (PAL)] (*5*), which would have created shallower and more dynamic redoxclines than found in modern oceans, along with the widespread occurrence of oxygen minimum zones along productive continental margins (*6*, *7*). Carbonate carbon (δ^13^C_carb_), carbonate-associated sulfur (δ^34^S_CAS_), and uranium (δ^238^U_carb_) isotope compilations have been used to infer that Ediacaran-Cambrian oxygen levels were highly dynamic, with multiple ocean oxygenation events (OOEs) occurring globally over 1- to 10-Myr timescales (*8*–*11*).

There is a statistically significant antithetic relationship between the marine δ^13^C_carb_ and δ^238^U_carb_ records throughout the late Ediacaran to lower Cambrian interval (Fig. 1, A and B) (*12*). These co-occurring trends suggest that OOEs were linked to the dynamics of the global carbon cycle. The available data, while discontinuous, also suggest a baseline δ^238^U_carb_ trend toward more positive values through the Ediacaran to mid-Cambrian interval (~580 to 515 Ma), which may indicate a progressive increase or stabilization of environmental oxygen, but this was punctuated dynamically by OOEs and anoxic episodes (*12*). Pulsed shallow marine OOEs, as inferred from δ^238^U_carb_, δ^34^S_CAS_, and δ^13^C_carb_ records, occur at δ^13^C_carb_ maxima and throughout the subsequent falling limbs in δ^13^C_carb_ profiles (*11*–*13*). Here, falling δ^13^C_carb_ and δ^34^S_CAS_ reflect progressive oxygenation of the deeper water column, resulting in decreasing organic carbon and pyrite burial (*13*), consistent with coeval increases in δ^238^U_carb_. Conversely, rising limbs in δ^13^C_carb_ and δ^34^S_CAS_ profiles (and falling δ^238^U_carb_) reflect marine deoxygenation conducive to the progressive burial of organic carbon and pyrite, which leads to gradual atmospheric oxygenation (*13*). Thus, trends in each isotope system reflect global environmental oxygen availability, ultimately linked to the long-term carbon and sulfur cycles.

However, while these proxy systems can reveal trends in global environmental oxygenation, they do not inform ambient oxygen availability in regional shallow marine environments, where most early metazoans lived, and are also not sensitive to regional redox variability, especially on short timescales. Therefore, other regional redox proxy systems are necessary to infer more precise levels of oxygen and ecosystem habitability. Moreover, the biotic evolutionary response to redox change, as well as regional heterogeneity, can only be understood if local geochemical proxies are combined with local biotic data.

There has been much debate as to the actual drivers of an evolutionary response to oxygenation. Rising oxygen levels (potentially driven by increased productivity and organic matter burial) may have deepened the redoxcline, thereby extending habitable water depths (*14*). Enhanced oxygenation may also have enabled the evolution of more metabolically costly ecologies such as mobility and carnivory (*15*), as well as the ability to produce skeletal hard parts, thus promoting animal-sediment mixing [e.g., (*16*)] and evolutionary escalation (*17*). Conversely, the presence of shallow marine anoxia itself might have formed physical barriers to dispersal (*18*), and, thus, dynamic redox variability over evolutionary timescales may have promoted reproductive isolation and speciation (*19*).

The early Cambrian record of the Siberian Platform shows that phases of increased biodiversity, ecosystem complexity, and individual body sizes of reef-associated archaeocyath sponges and other metazoans synchronously coincided with the expansion of reef habitats on the shallow marine shelf (*14*, *20*). These biotic changes coincide with positive carbonate-carbon isotope (δ^13^C_carb_) peaks, reflecting carbon cycle perturbations and implying a (possibly transient) deepening of the redoxcline associated with each OOE (*12*–*14*). These intervals also coincide with increased rates of speciation during major sea-level lowstands, which may have permitted extensive oxygenation of shallow waters over the entire craton, providing oxic corridors for dispersal and the creation of new founder communities (*19*). Cambrian oxygen levels and their variability have not, however, been constrained by local paleoproxy data, and to trace the oxygen requirements of Cambrian metazoans necessitates the integration of local redox data with in situ*,* rather than reworked, fossil fauna (*21*, *22*).

While a rise in oxygen levels has been suggested to facilitate the evolution of costly ecologies [e.g., (*15*)], it does not offer a definitive mechanism for the origination of new clades or the appearance of key evolutionary innovations, such as a biomineralization, bilaterality, and segmentation, but may rather provide a viable mechanism by which existing metazoan clades are able to expand their habitat range and so diversify (Fig. 1C). The appearance of bilaterians required the formation of new mechanisms to coordinate multiple cell types and an expanded capacity to coordinate gene activities to generate new tissues and organs. However, how much developmental capacity was present in the last common ancestor of protostomes and deuterostomes, and how much arose independently in major clades, remains unclear (*23*). New spatial and temporal regulatory hierarchies enabled widespread co-option events in metazoans, possibly during the late Ediacaran, that allowed the independent appearance of many key metazoan innovations such as skeletons, segmentation, a nervous system and head, appendages, sensory systems, and a regionalized gut (*23*)*.* These are the hallmark of the Cambrian Radiation, and such a rapid and independent acquisition of different, novel characters in many different clades implicates possibly numerous external triggers (*23*).

All clades predate their first appearance of macroscopic representatives in the fossil record, which offers only a minimum estimate age for the clade (*24*, *25*). This is not only due to vagaries of preservation, sampling, and acquisition of taxonomically identifiable features but also suggests that the developmental and morphological novelties required to define a new clade are decoupled from the ecological and/or extrinsic drivers and their feedbacks that allowed these clades to proliferate and, hence, rise to ecological prominence in the fossil record. For example, molecular clocks suggest that the euarthropods may have appeared in the latest Ediacaran and trilobites in the Fortunian (broadly coincident with trilobite-like traces); however, trilobite body fossils did not appear until 521 Ma, after which they underwent a rapid radiation, i.e., an increase in species richness accompanied by morphological diversification (Fig. 1C) (*26*). In other words, developmental capacity is decoupled from morphologic complexity, and the developmental capacity that early metazoans had was not realized and promoted until subsequent environmental (such as oxygen or productivity) and/or ecological (such as competition and macro-predation) changes occurred, which together characterized and fueled the early Cambrian Radiation (*23*)*.*

**Fig. 2.**
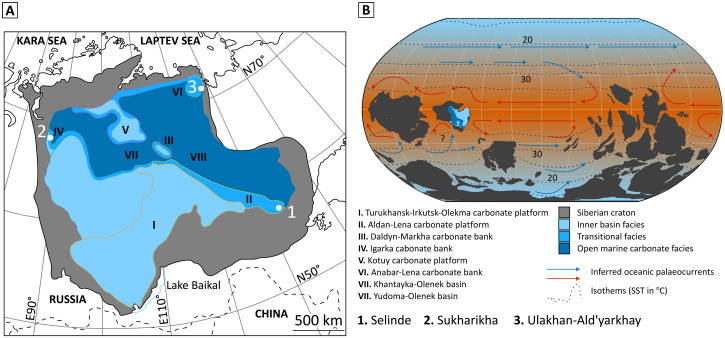
Early Cambrian of the Siberian craton. (**A**) Paleofacies map of Cambrian stages 2 to 3 (~529 to 514.5 Ma) showing the distribution of six banks/platforms (I to VI) and the study sites (1 to 3) [after (*28*)]. (**B**) Cambrian paleogeography at 521 Ma [after (*47*, *163*)] with position of the Siberian craton and possible ocean currents. Modeled sea surface temperature and isotherms [after (*48*)].

Here, we present an integrated, high-resolution early Cambrian record from ~527 to 519 Ma (Cambrian stages 2 to 3, Tommotian-Atdabanian), constrained within a chemostratigraphic age model of globally established and correlated δ^13^C_carb_ oscillations (from cycles 6p/7p to IV) (*27*), to understand potential relationships between the Cambrian Radiation and associated water column redox conditions. We combine major element and multiple paleoredox proxy data, including Fe speciation, redox-sensitive trace elements, and I/(Ca + Mg) ratios, with skeletal metazoan distributions and metrics of diversification, from three broadly coeval shallow water localities on the biodiversity hotspot of the lower Cambrian Siberian Platform (Fig. 2A). These data constrain the oxygen demands of early Cambrian skeletal metazoans and inform the drivers of the Cambrian Radiation coincident with the global appearance and diversification of trilobites, representing one of the earliest, major motile and skeletal groups.

### Geological background

**Fig. 3.**
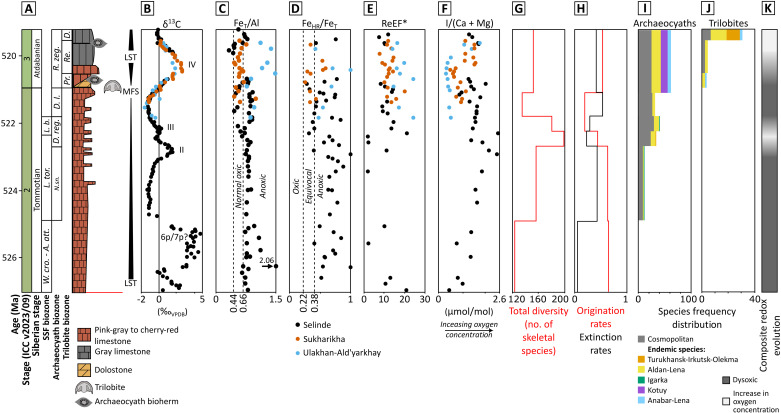
Summary of redox data and metazoan dynamics through the early Cambrian on the Siberian Platform. (**A**) Composite stratigraphy [after (*28*, *164*–*166*)]. MFS, maximum flooding surface. LST, lowstand systems tract. Siberian biozone subdivision after (*19*) and references therein. (**B**) C isotope (δ^13^C_carb_) record [from (*75*, *166*, *167*)] with the first appearance of trilobites and archaeocyath reefs. (**C**) Fe_T_/Al. (**D**) Fe_HR_/Fe_T_ ratios. Dashed lines in (C) represent the empirically derived normal oxic range (*36*). Dashed lines in (D) represent the empirically derived normal oxic and anoxic thresholds with an equivocal zone between (*37*). (**E**) Re enrichment factors (ReEF*). (**F**) I/(Ca + Mg). (**G**) Total diversity on the Siberian Platform from (*59*). (**H**) Rates of origination and extinction of skeletal species on the Siberian Platform (*59*). (**I**) Distribution of cosmopolitan and endemic species of all archaeocyath sponges on the Siberian Platform (*19*). (**J**) Distribution of cosmopolitan and endemic species of trilobites on the Siberian Platform (see table S7). (**K**) Reconstructed composite redox evolution.

The Siberian Platform formed a vast tropical continent (Fig. 2B) with several episodically isolated carbonate banks/platforms (Fig. 2A) (*28*, *29*) that housed over a third of all known skeletal taxa from the early Cambrian (*13*). The Siberian fauna diversified through the early Cambrian (Fig. 3G), with the total number of fossil-bearing sites increasing from 25 to 46 and expanding in geographical area through δ^13^C_carb_ peaks II and III (~523 to 522 Ma, stage 2, middle Tommotian; Fig. 3 and table S6). The δ^13^C_carb_ peak IV (~521 to 520 Ma, stage 3, early Atdabanian) coincides with a major sea-level lowstand (Fig. 3, A and B) and also with an acme of metazoan biodiversity (Fig. 3G) (*14*, *20*), high rates of overall origination (Fig. 3H), archaeocyath speciation and endemism (*14*) (Fig. 3I), and the global appearance and rapid diversification of trilobites (Fig. 3J and table S7) (*30*–*33*).

Here, we interrogate the biotic and geochemical record of three geographically disparate sites on the Siberian Platform: Selinde River, Sukharikha River, and Ulakhan-Ald’yarkhay Brook (Fig. 2A). Samples from the Selinde River section (Aldan-Lena carbonate platform, SE Siberian Platform) record the longest temporal interval, from δ^13^C_carb_ excursion 6p/7p (~527 Ma) to after δ^13^C_carb_ peak IV (~519.5 Ma) (Fig. 2A and figs. S1 and S2), but those from the Sukharikha River (Igarka carbonate bank, NW Siberian Platform) and the Ulakhan-Ald’yarkhay Creek at the Khara-Ulakh Mountains (Anabar-Lena carbonate platform, NE Siberian Platform; Fig. 2A) broadly cover the δ^13^C_carb_ peak IV interval only (Fig. 3A and figs. S1 and S2), with Sukharikha occupying a slightly deeper bathymetric position (see the Supplementary Materials) (*28*, *34*). These successions alone record diverse biotas of >100 species (Fig. 3) of mostly reworked small shelly fossils (SSFs), in situ archaeocyath sponge reefs, and the first trilobites (both in situ and slightly reworked), which appear coincident with δ^13^C_carb_ peak IV (see the Supplementary Materials; Fig. 3J, fig. S1, and table S5). This association of the δ^13^C_carb_ and metazoan fossil records is found throughout many other sections on the Siberian Platform (*13*, *27*, *35*).

## RESULTS

### Low oxygen levels and oscillations in the early Cambrian

Our multi-proxy approach allows a detailed assessment of the redox evolution on the Siberian Platform (see Materials and Methods for methods and geochemical framework, and tables S1 to S5 for all data). Samples with iron concentrations >0.5 wt% (*36*) were analyzed for total iron/aluminum (Fe_T_/Al), highly reactive/total iron (Fe_HR_/Fe_T_), and pyrite-bound iron/highly reactive iron (Fe_py_/Fe_HR_) ratios (Fig. 3, C and D, and fig. S1H). At Selinde, Fe_T_/Al ratios are consistently above the calibrated anoxic threshold (0.66) (*36*) through the lower part of the section, followed by fluctuations to lower values in the uppermost Tommotian and lower Atdabanian (Fig. 3C). Fe_HR_/Fe_T_ ratios are generally consistent with Fe_T_/Al ratios, with values above the anoxic threshold [0.38; (*37*)], with the exception of some samples that fall in the equivocal zone in the uppermost Tommotian (Fig. 3D). Fe_py_/Fe_HR_ ratios are all low (fig. S1H), indicating negligible free sulfide in the water column and during diagenesis (*37*). At Sukharikha, most Fe_T_/Al and Fe_HR_/Fe_T_ values fall below the anoxic threshold, with occasional elevated values, while low Fe_py_/Fe_HR_ ratios again indicate limited sulfide production. At Ulakhan-Ald’yarkhay, Fe_T_/Al and Fe_HR_/Fe_T_ ratios consistently fall above the anoxic thresholds, with low Fe_py_/Fe_HR_ ratios.

The common occurrence of enrichments in Fe_T_/Al and Fe_HR_/Fe_T_ ratios indicates at least periodic anoxic mobilization of Fe^2+^ and subsequent precipitation, while low Fe_py_/Fe_HR_ ratios suggest limited production of sulfide, even during diagenesis. However, this does not necessarily mean that the overlying water column was anoxic since upwelling of anoxic-ferruginous deeper waters into better ventilated shallower waters would result in precipitation of Fe^2+^ and hence Fe enrichments in the sediment (*38*). Thus, to provide further insight into redox conditions at the site of deposition, we use a refined approach for calculating enrichment factors (termed EF^*^) for redox-sensitive trace metals (e.g., U, Mo, and Re) in carbonate-rich lithologies (see Supplementary Materials) (*39*). Resultant EF^*^ values for U and Mo are typically low at all three sites (~1; figs. S2F and S3 and tables S2 and S3), indicating essentially no enrichment in either element, and, hence, anoxia was apparently not prevalent at the site of deposition (*40*). By contrast, all sections are highly enriched in Re (Fig. 3E and fig. S2H). Re has a higher reduction potential than U and Mo and begins to accumulate in sediments under dysoxic conditions [dysoxic conditions being defined as ~8.8 to 88 μmol/kg O_2_, or 9 to 89 μM; (*41*)] at the sediment-water interface (*42*). Hence, given that Sukharikha and Ulakhan-Ald’yarkhay are distal from continental input, our combined data are consistent with dysoxic conditions on the Siberian Platform, which promoted oxidation of Fe^2+^ in upwelling anoxic-ferruginous deeper waters.

We further measured I/(Ca + Mg) ratios to potentially reveal more subtle variations in oxygen availability at each site and through time. The data show no significant correlation with either total organic carbon or stable oxygen isotopes (δ^18^O) (figs. S4 to S6), implying minimal influence of organic matter or diagenesis on iodine concentrations. I/(Ca + Mg) ratios are low at all sites, relative to the typical “oxic” threshold of ~2.6 μmol/mol (*43*, *44*): Selinde (0.42 to 2.50 μmol/mol, mean = 1.47 μmol/mol), Sukharikha (0.47 to 1.56 μmol/mol, mean = 1.02 μmol/mol), and Ulakhan-Ald’yarkhay (0.29 to 1.73 μmol/mol, mean = 0.65 μmol/mol) (Fig. 3F), suggesting limited iodide oxidation within the water column at the depth of carbonate formation (*45*). Such values suggest that oxygen levels were low and predominantly conducive to iodate reduction (i.e., the water column was likely manganous to nitrogenous). Carbonate I/(Ca + Mg) ratios of <2.5 μmol/mol (equivalent to IO_3_^−^ < 0.25 μM in seawater) indicate seawater [O_2_] of <20 to 70 μM in modern and ancient oceans (*44*, *46*) and are thus consistent with our interpretation of prevalent dysoxia on the platform. These data are consistent with modeled shallow marine O_2_ concentrations throughout the early Palaeozoic (*4*). However, Selinde records the highest average ratios and Ulakhan-Ald’yarkhay records the lowest (Fig. 3F), implying a meaningful difference in oxygen concentrations between each of these sections and thus spatial variability in oxygen concentrations across the platform. In sum, our data reveal that Selinde was generally better oxygenated in the upper half of the section, but across δ^13^C_carb_ peak IV, [O_2_] was progressively depleted from Sukharikha to Ulakhan-Ald’yarkhay (Fig. 3F).

While our geochemical data necessarily represent time-averaged concentrations of homogenized samples (and hence integrate short-term variability), the stratigraphic trends in I/(Ca + Mg) ratios can be attributed to longer-term changes in relative oxygen concentrations (at levels sufficient to oxidize iodide) at the locus of carbonate mineral formation. I/(Ca + Mg) ratios at Selinde progressively increase through the lower half of the section to peak values broadly coincident with δ^13^C peaks II and III before falling to intermediate values, while ratios at Sukharikha and Ulakhan-Ald’yarkhay increase after peak IV, coincident with the sedimentation occurring in the shallowest waters (Fig. 3, A and F). Despite overall dysoxic conditions, I/(Ca + Mg) ratios therefore indicate the development of better oxygenated conditions coincident with δ^13^C peaks II, III, and after peak IV. Samples that show a progressive increase in I/(Ca + Mg) ratios in the lower half of the section at Selinde also show persistently elevated Fe_HR_/Fe_T_ ratios, but during the interval of sustained intermediate values at Selinde and Sukharikha (i.e., between δ^13^C peaks III and IV), Fe_HR_/Fe_T_ ratios fluctuate to values that are sometimes in the equivocal zone, supporting unstable but overall better oxygenated conditions. These data support the hypothesis that the Siberian Platform was primarily dysoxic, but with transient intervals of moderately enhanced oxygenation over δ^13^C peaks II, III, and IV. Oxygen levels during these better oxygenated intervals remained, however, at relatively low levels.

## DISCUSSION

### A shallow redoxcline limited habitable space

Our combined paleoredox data provide the first independent confirmation that oceanic oxygen levels remained low in the early Cambrian even within densely populated and biodiverse marine basins. Notwithstanding possible diagenetic effects (see Materials and Methods, Redox proxy systematics section), the low I/(Ca + Mg) values are consistent with all other proxy data, including redox-sensitive trace metal EFs. Our data also show modest, regional shallow marine oxygenation on the Siberian Platform coincident with major, and globally correlatable, positive δ^13^C_carb_ excursions, thereby supporting the hypothesis that these events represent global shallow marine OOEs (*13*). On the basis of available I/(Ca + Mg) data, the general levels of oxygenation appear to have been broadly similar, but possibly slightly elevated, to those recorded in some terminal Ediacaran (550 to 539 Ma) carbonate environments (*46*).

**Fig. 4.**
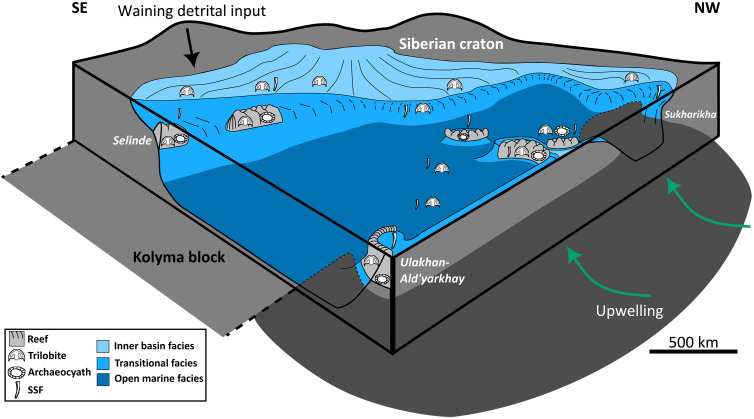
Block diagram summarizing the spatial redox and metazoan distribution on the Early Cambrian Siberian Platform. Paleofacies after (*28*); SSF distribution after (*58*); archaeocyath sponge distribution after (*19*); trilobite distribution after (*31*, *58*).

Our data, however, strongly suggest that OOEs were unlikely to have established pervasively oxic conditions below reef margins, and the redoxcline was therefore very shallow and dynamic, significantly defining and limiting habitable space. Deeper ferruginous waters were apparently present off the platform margin, with episodes of dynamic oceanic upwelling resulting in elevated Fe_HR_/Fe_T_ values in shallow marine settings, despite locally dysoxic conditions (Fig. 4) (*18*). Potential ocean circulation dynamics based on a recent global plate reconstruction (*47*) and modeled Cambrian oceanic surface temperatures (*48*) suggest that the southwest margin of the platform (the region of Ulakhan-Ald’yarkhay) may have been a site of nutrient upwelling and potentially anoxic waters (Fig. 2B) (*19*) but with upwelling precluded by the presence of the Kolyma-Omolon terrain on the present-day eastern margin of the platform (Fig. 4) (*49*, *50*). Upwelling of nutrients would have sustained high primary productivity in this region and reduced oxygen availability in the vicinity of upwelling relative to coeval sections on the platform. Given the similarity in the I/(Ca + Mg) trends at Sukharikha and Ulakhan-Ald’yarkhay, it is likely that they were both in close proximity to the open ocean and the influence of productivity-driven anoxic waters to the south (Fig. 4), with the higher I/(Ca + Mg) ratios at Selinde likely reflecting its more distal setting from anoxic waters and the open ocean.

### Metazoan habitat expansion and diversification in response to oxygenation events

Combining local redox data with the temporal and spatial distribution of metazoans on the Siberian Platform reveals the biotic response to oxygenation. Sukharikha shows a low overall biodiversity (i.e., species richness) and an absence of archaeocyath reefs in early stage 3 (fig. S1 and table S5), consistent with its deeper position and the influence of proximal anoxic waters. Although overall metazoan biodiversity during the same interval is high at Selinde, this consists mostly of SSFs (mainly hyoliths). These are generally slightly reworked (i.e., derived from the same sedimentary setting in which they were deposited) in the upper part of this section (beds 24 to 54) (fig. S1). Only three reef-associated archaeocyath sponges and five trilobite species are in situ (fig. S1). Metazoan reefs (with associated SSFs and trilobites) are recorded before peak IV at Ulakhan-Ald’yarkhay (Unit 6; fig. S1), suggesting that they were established before those at Selinde, which is also consistent with the shallower bathymetric position of Ulakhan-Ald’yarkhay (see the Supplementary Materials). A reworked fossil assemblage is preserved in limestone units below these reefs, but few fossils are preserved after δ^13^C_carb_ peak IV (fig. S1). Our paleoredox data indicate that these metazoan reefs grew under dynamic, primarily low-oxygen backgrounds before an increase in oxygen concentrations after δ^13^C_carb_ peak IV, and were thus either adapted to low-oxygen conditions or potentially colonized the sea floor during relatively short-lived higher-oxygen conditions but aliased by time-integrated sample homogenization [e.g., (*21*, *22*)].

At the base of the Selinde section (beds 15 to 23), marked by a transgressive surface, SSFs are by contrast more variably reworked, including those derived from other sedimentological settings, and preserved as casts replaced by glauconite and phosphate. High generic diversity during this interval may be due to the concentration of reworked skeletal fossils at the base of transgressive lags (*12*). Both early glauconitization and phosphatization are indicative of replacement under variable porewater redox conditions conducive to intense Fe-P cycling (*51*–*53*). Such replacement also tends to be associated with low sedimentation rates, stratigraphic condensation, and reworking (*54*–*56*). The unusually high abundance of authigenic glauconite in the Cambrian (*57*) indicates that these porewater redox oscillations occurred near the sediment/water interface, requiring low-oxygen and fluctuating conditions at or near the seafloor. The high abundance of glauconitized and phosphatized SSFs therefore supports the hypothesis that dynamic, low-oxygen conditions were pervasive on the Siberian Platform. While our data are in agreement with this, fluctuations in oxygen concentrations responsible for early glauconitization and phosphatization likely occur on even shorter timescales than our dataset is able to capture, further indicating highly dynamic redox conditions.

Other sites on the Siberian Platform that are characterized by a high abundance of fossils were also restricted to the shallowest settings, representing deposition at water depths within fair-weather wave to storm-wave base (see the Supplementary Materials) (*19*, *58*). Fossil distributions throughout these sections suggest, then, that lower-oxygen conditions in deeper waters restricted most skeletal metazoans to shallow, bank tops where oxygen concentrations were sufficiently high, and potentially enhanced by physical ventilation mechanisms, throughout δ^13^C peak 6p/7p and across peaks II and III.

**Fig. 5.**
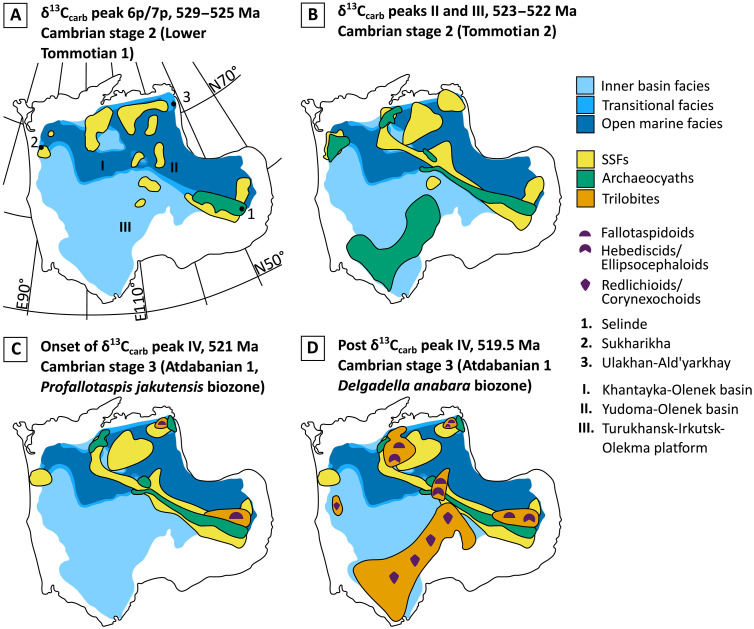
Metazoan temporal and spatial distribution on the early Cambrian Siberian Platform, from 529 to 519.5 Ma. (**A**) Cambrian Stage 2, 529-525 Ma. (**B**) Cambrian Stage 2, 523-522 Ma. (**C**) Cambrian Stage 3, 521 Ma. (**D**) Cambrian Stage 3, 519.5 Ma. Paleofacies after (*28*); SSF distribution after (*58*); archaeocyath sponge distribution after (*19*); trilobite distribution after (*31*, *58*).

Archaeocyath sponges diversified and underwent habitat expansion around δ^13^C peaks II, III, and IV (~521 to 520 Ma, early Atdabanian) (Figs. 3I and 5) (*19*). Trilobites appear and diversify before and during peak IV (*59*) from three genetically distinct populations. Species of the genus *Profallotaspis* (superfamily Fallotaspidoidea) first appear in the *Profallotaspis jakutensis* biozone and increase in diversity to 31 species in the *Delgadella anabara* biozone (Figs. 3J and 5 and table S7). On the basis of our paleoredox proxy data, δ^13^C peaks II, III, and IV can be interpreted as moderate OOEs that punctuated pervasively low-oxygen environments.

This diversification was accompanied by marked habitat expansion. Archaeocyaths notably expanded their ranges from the Aldan-Lena area to the Turukhansk-Irkutsk-Olekma and Kotuy carbonate platforms and the Daldyn-Markha carbonate bank (Fig. 3I) (*19*). Between peaks III and IV, archaeocyath geographic distribution contracted and only 32 species survived on the Aldan-Lena platform in the latest Tommotian, of which only five were endemic (*19*). During the peak IV interval, archaeocyaths again expanded their range to form endemic faunas in the Kotuy and Anabar-Lena banks (Fig. 3I) (*19*). Similarly, by the end of the peak IV interval, trilobites had diversified and extended their habitats after their first appearance (fallotaspidoids) in the reef settings of the Aldan-Lena (including Selinde) and Anabar-Lena (including Ulakhan-Ald’yarkhay) banks. They began to colonize the shallow calcareous-dolomitic muds of the inner Turukhansk-Irkutsk-Olekma basin (superfamillies Corynexochoidea and Redlichioidea) and the argillaceous-calcareous muds of the deeper Yudoma-Olenek and Khantayka-Olenek basins (the smallest species represented by the Family Hebediscidae and Superfamily Ellipsocephaloidea) (Figs. 3J and 5 and table S7), thus creating three distinct trilobite paleocommunities (*31*). Colonization of the previously unhabitated Turukhansk-Irkutsk-Olekma basin was the result of the apparently rapid appearance of numerous, short-lived endemic species (Figs. 3J and 5 and table S7).

During the peak IV interval, synchronous expansions of both diversity and habitat found in both archaeocyaths and trilobites coincident with an episode of oxygenation also occurred during a sea-level lowstand (Figs. 3A and 5). While archaeocyaths appear to have diversified before trilobites, beginning during the rising limb of δ^13^C_carb_ peak IV, this may not be the case due to possible aliasing of data. Trilobite fossil occurrences can be binned according to three relatively short-lived biozones, allowing a more precise stratigraphic placement within the peak IV interval (Fig. 3J), but archaeocyath data can only be placed within one biozone over the same interval (Fig. 3I). It is therefore not possible to determine whether the greatest increase in archaeocyath diversity and habitat expansion occurred before, during, or after peak IV. Regardless, these data support the hypothesis that when an OOE coincided with relatively low sea-level, this coupled environmental change promoted more extensive oxygenation of shallow waters over the entire craton, thus providing oxic corridors for dispersal and the creation of new founder communities (*19*). This is supported by the marked increase in both archaeocyath and trilobite diversity and habitat expansion during this interval (Figs. 3, I and J, and 5, C and D; and table S7).

We conclude that during the Cambrian Radiation, from ~528 to 519 Ma, the Siberian Platform was characterized by low-oxygen conditions, with a dysoxic water column in shallow marine depositional environments where metazoans lived. Our suggestion that early Cambrian metazoans, even mobile, calcified bilaterians such as trilobites, did not have high oxygen demands is supported by modern ecophysiological data indicating that all of the critical oxygen thresholds of function and ecology, including high-energy lifestyles, can be met at low oxygen levels [equating to ~22 μM or 8% of modern surface ocean levels; Fig. 3D (*60*)]. In the modern ocean, metazoans with the energetically costly lifestyle of carnivory may occur at very low oxygen concentrations (~0 to 9 μM), but the diversity of species increases with increasing oxygen concentrations with peak diversity being achieved from ~22 to 45 μM (*15*, *60*). It is possible, however, that even the modest oxygenation events discerned here may have moved metazoans past critical ecological thresholds that further facilitated diversification.

Adaptation to low oxygen levels is suggested to have been an ancestral character of several groups, including trilobites, which appear to have had the respiratory proteins and gill structures to cope with low oxygen levels (*61*, *62*). The upper limb branches of Cambrian biramous arthropods (including corynexochide and olenide trilobites) had dumbbell-shaped filaments, which would have overcome the limited effectiveness of the distributed system of cuticular diffusion found in small-sized animals (*62*). If early fallotaspidoid trilobites had relatively short carapaces with long pleurae, this would have allowed these forms to extend filament height and gill shaft length, thus maximizing the respiratory surface area and maintaining oxygen intake at higher levels, similar to some extant crustaceans inhabiting oxygen-depleted waters (*63*). Such morphologies might explain the extensive distribution of hebediscid trilobites over the Siberian Platform (Fig. 5) (*64*). Furthermore, modern sponges can tolerate oxygen levels of <4% PAL (*65*, *66*), and the slow, motile gastropods *Patella* spp. and *Haliotis fulgens*, which both belong to ancient lineages, are able to decrease their oxygen demands under hypoxia (*67*, *68*). Predatory chaetognaths, as represented by protoconodonts (*69*, *70*), have an efficient diffusive respiratory system given their small size, although they lack gills (*71*). It is possible, however, that many other early Cambrian metazoans may have had inefficient respiratory systems, potentially explaining the small size of Cambrian SSFs [not ≥80 mm but most <30 mm; (*20*, *72*–*75*)] compared to Ordovician representatives of the same groups, which were one to two orders larger (*76*, *77*).

### Toward a global model

The origin of major metazoan clades always predates the appearance of their first macroscopic representatives in the fossil record, and while environmental changes and ecological feedbacks may have been required to realize their developmental capacities, it is not yet clear whether these relate to any changes in ambient oxygen levels. Increases in oxygen availability may, however, have enabled metazoans to take advantage of both developmental potential and ecological opportunities subsequent to the establishment of a major group. Here, we show that increases in oxygen concentrations may have provided a viable mechanism by which existing groups were able to expand their habitat range and hence diversify, which was perhaps enhanced by reaching critical ecological thresholds. This is often accompanied by morphological and ecological diversification of these clades. However, our data show that high oxygen levels were not required for Cambrian metazoan diversification and radiation. Against the backdrop of low-oxygen conditions, pulsed, but only modest, increases in shallow marine OOEs facilitated an increase in species richness via habitat expansion in archaeocyath sponges and trilobites, which occurred approximately coincident with global δ^13^C_carb_ maxima recorded by peaks II, III, and IV. It was these dynamic, but consistently low, oxygen conditions within shallow marine environments, combined with cycles of geographical isolation and expansion, that drove diversification in the important biodiversity hotspot of the Siberian Platform.

The synchronous nature of δ^13^C_carb_ peaks and OOEs suggest that these likely record global redox dynamics (*12*), with oxygenation during peak IV potentially being further enhanced and more regionally extensive on the Siberian Platform due to its coincidence with a major sea-level lowstand. While this study, therefore, represents an integration of local paleoredox and biotic data, it creates a model that has a potentially wide application, as δ^13^C_carb_ cycles are extensively documented from many regions and have been demonstrated to be globally synchronous (*12*, *78*). Our data are also consistent with models that infer global OOEs through the correlation and integration of the C, S, and U isotope systems (Fig. 1, A and B) (*11*–*13*). Similar integrative studies are required for other Cambrian successions to better constrain redox heterogeneity and its impact on diversification, and while other regions may show higher oxygen levels during global OOEs (during δ^13^C_carb_ peaks), we argue that high-oxygen conditions are not required to explain the Cambrian Radiation.

## MATERIALS AND METHODS

### Sample preparation

Samples were halved with a diamond saw to retain archive material, and weathered surfaces were removed from one half. The samples were washed and dried at 40°C. Samples with visible signs of alteration and/or veining were rejected at this point. Halved samples were crushed and pulverized to homogeneous powder (<60 μm) using a tungsten carbide Tema laboratory disc mill.

### Total digestion

After ashing at 550°C for 8 hours, the samples were quantitatively dissolved in trace metal grade HNO_3_, HF, and HClO_4_, heated in open polytetrafluoroethylene cups and left to dry fully over a period of 24 hours before the addition of H_3_BO_3_ to prevent the formation of Al complexes. Dry residues were then dissolved in concentrated HNO_3_ and diluted with ultrapure 18 megohm H_2_O. Major element concentrations were analyzed using inductively coupled plasma optical emission spectrometry (ICP-OES, Thermo Fisher Scientific iCAP 7400), and trace element concentrations were measured using ICP mass spectrometry (ICP-MS, Thermo Fisher Scientific iCAPQc) at the School of Earth and Environment, University of Leeds. Total digestions of a standard material (SBC-1, US Geological Survey) yielded values within the certified range for all elements analyzed (<4%).

### Fe speciation

Iron speciation analyses follow the established method of (*79*), as modified in (*38*). An initial leach targeting iron bound in carbonate phases (Fe_carb_) used Na-acetate, buffered to pH 4.5 with acetic acid and agitated at 50°C for 48 hours. This was followed by a 2-hour iron (oxyhydr)oxide (Fe_ox_) extraction in Na-dithionite buffered to pH 4.8, and then a final extraction of magnetite (Fe_mag_) with ammonium oxalate for 6 hours. All steps of the sequential leach were performed at the Cohen Laboratories, University of Leeds, and resultant solutions were analyzed for Fe using a Thermo Fisher Scientific iCE-3000 series flame atomic absorption spectrometer, with replicate extractions for each step yielding relative standard deviations (RSDs) of <5%. To ensure accuracy, Fe speciation reference material (WHIT) (*80*) was run alongside each batch.

The concentration of pyrite iron (Fe_py_) was determined through a boiling chromous chloride [Cr(II)Cl_2_] distillation with a pre-leach in boiling 6 M HCl for quantitative extraction of acid volatile sulfide (AVS). Pre-leaching confirmed that no AVS was present. Weight percent Fe_py_ was determined gravimetrically after stoichiometric precipitation of Ag_2_S.

### I/(Ca + Mg)

Measurement of I/(Ca + Mg) ratios followed the procedure of (*81*). One hundred milligrams of powder was washed with 18 megohm of H_2_O and dried at 40°C for 48 hour. Five milligram of resultant powder was weighed for analysis. The samples were digested with 3% HNO_3_. Iodine was stabilized with 3% ammonium hydroxide to create a mother solution. This was made to volume with a matrix solution of 3% HNO_3_ + 0.5% ammonium hydroxide + 3% methanol. The solution was diluted with matrix to produce 50 ± 5 μg/ml Ca concentrations in each sample. Ca and Mg concentrations were measured using ICP-OES (Varian Vista Pro ICP-OES), and I concentrations were measured using ICP-MS (high-resolution single collector ICP-MS, AttoM) at the University of Edinburgh, using a Tellurium internal standard. JCP-1 coral standard was analyzed as a reference material with RSDs <4%.

### Elemental EFs

EFs provide a quantification of the level of accumulation of redox-sensitive trace metals (*40*, *82*), and EFs are commonly calculated for siliciclastic sediments by the following

$${\text{Element}}_{\mathrm{EF}}=\frac{\text{Element}/\mathrm{Al}}{{\text{Element}}_{\mathrm{UCC}}/{\mathrm{Al}}_{\text{UCC}}}$$ (1)

where UCC represents upper continental crust [here, we use concentrations in (*83*) except for Re, which is not reported, for which we use values in (*84*)]. However, using EF values in sediments rich in carbonates is problematic as low Al concentrations in such sediments result in elevated EFs relative to siliciclastic sediments (*85*). This may be navigated by calculating elemental excess concentrations (*39*), which are calculated by the following

$${\text{Element}}_{\text{excess}}={\mathrm{Element}}_{\text{sample}}-\left({\mathrm{Al}}_{\text{sample}}\times \frac{{\mathrm{Element}}_{\mathrm{UCC}}}{{\mathrm{Al}}_{\mathrm{UCC}}}\right)$$ (2)

These values are then used to calculate revised EF values (EF^*^) for carbonate-rich lithologies (*39*)

$${{\text{Element}}_{\mathrm{EF}}}^{*}=\frac{{\text{Element}}_{\text{excess}}/{\text{Element}}_{\text{UCC}}}{{\text{Element}}_{\text{UCC}}}$$ (3)

Using EF^*^ allows the assessment of elemental EFs in carbonate rocks and furthermore facilitates comparison to siliciclastic lithologies.

### Redox proxy systematics

We deploy a multi-proxy approach to assess the redox evolution of the Siberian Platform. Fe speciation facilitates the assessment of redox conditions by measuring the concentration of Fe_HR_ (that is, a highly reactive Fe pool composed of Fe in carbonate and ferric oxide minerals, magnetite, and pyrite—Fe_carb_, Fe_ox_, Fe_mag_, and Fe_py_) and normalizing these data to Fe_T_ (the total concentration of Fe within a sample) (*37*, *79*). Fe speciation has been calibrated in a variety of modern and ancient settings and is able to constrain oxic and “anoxic” conditions (*37*, *38*, *86*), whereby ratios ≤0.22 are commonly indicative of oxic water column conditions. Ratios ≥0.38 are commonly indicative of precipitation of Fe–bearing unsulfidized mineral phases and Fe sulfides under anoxic water column conditions in ferruginous and euxinic settings, respectively (*87*, *88*). These absolute thresholds may not be applicable to all depositional environments (e.g., high rates of deposition may obscure anoxic signals due to decreased Fe_HR_/Fe_T_ values or mineral transformation during diagenesis), and, hence, an equivocal zone is defined between these thresholds (*37*). Fe_T_/Al ratios may be used to further assess redox conditions in the case of obscured values due to diagenesis. Values >0.66 are indicative of deposition under anoxic waters, with a typical oxic range of 0.44 to 0.66 (*36*).

Anoxic ferruginous and euxinic conditions may be distinguished by the proportion of pyrite-bound Fe (Fe_Py_) within the Fe_HR_ pool, whereby values >0.8 are indicative of deposition under a euxinic water column (*37*). The potential for addition of Fe_HR_ into carbonates during diagenesis means that care must be taken when analyzing carbonates for Fe speciation. Calibration of Fe speciation data from carbonates suggests that samples with Fe_T_ > 0.5 wt% may record robust results, providing that no deep burial dolomitization has occurred (*36*). Fe speciation is, however, best deployed in addition to other proxies for water column redox conditions (*38*), and, therefore, we use such a strategy by undertaking analysis of redox-sensitive trace elements and I/(Ca + Mg) ratios to more accurately constrain redox conditions on the Siberian Platform.

Redox-sensitive trace element concentrations may be used to provide further constraints on redox conditions in modern and ancient settings (*40*, *85*, *89*, *90*). Of particular note are the behaviors of U, Mo, and Re. Under oxic conditions, Mo and U behave conservatively and occur primarily as MoO^2−^_4_ and UO_2_(CO_3_)^4−^_3_, with oxidation states Mo(VI) and U(VI) (*85*). Under reducing conditions, U(VI) is reduced to insoluble U(IV), resulting in enrichments in the sedimentary record (*91*). Mo may be moderately enriched under reducing conditions but becomes significantly enriched in the presence of free sulfide and is “activated” at a critical HS^−^ activity to form thiomolybdates (MoO_x_S^2−^_4-x_) (*92*). Mo concentrations may also be enriched by the function of a Mn-Fe particulate shuttle, whereby Mn-Fe (oxyhydr)oxides adsorb molybdate oxyanions, transporting them to the sediment-water interface (*82*). Calibration in modern and ancient settings means that enrichments in Mo and U can be used to assess the redox state of the depositional environment and whether a particulate shuttle is active (*40*). In oxic settings, Re is present as soluble Re(VII) and is reduced to insoluble Re(IV) under dysoxic conditions (*90*). Re enrichment is considered to be a reliable indicator of dysoxic conditions due to accumulation in the sediment at O_2_ penetration depths of < ~1 cm (*42*).

I/(Ca + Mg) ratios in carbonates are commonly used to assess redox conditions due to the sensitivity of I to redox conditions in seawater (*45*, *93*). The technique exploits the incorporation of oxidized iodate (IO_3_^−^) into the calcite and dolomite crystal lattice in place of the carbonate (CO_3_^2−^) ion. It has been shown experimentally that reduced iodide (I^−^) does not similarly substitute for the carbonate ion, and, thus, iodine concentrations may be used to assess redox conditions (*45*). Iodine has a high oxidation potential, similar to Mn, and is therefore sensitive to intermediate oxygen concentrations (*45*, *94*). The proxy has been calibrated in a variety of modern and ancient settings and is thus able to assess ancient redox conditions (*45*, *94*–*98*). Some studies suggest that I/(Ca + Mg) ratios may be used as proxies for absolute oxygen concentrations and that I/(Ca + Mg) values <2.5 to 3 μmol/mol in modern foraminiferal samples indicate formation under dysoxic conditions (*44*–*46*); however, defining absolute concentrations is complicated. Low I/(Ca + Mg) values have been reported in the presence of oxic waters due to the slow kinetics of iodide oxidation (*94*), and diagenesis has been shown to reduce I/(Ca + Mg) values (*96*, *99*).

Should a sample set be altered by meteoric diagenesis, sediment- or fluid-buffered diagenesis, and dolomitization, it is possible that I/(Ca + Mg) values may be lowered by as much as ~1.5 to 100% (*99*). Hence, I/(Ca + Mg) values must be carefully considered and a multi-proxy approach to corroborate the interpretation of values is preferable. We find no evidence for meteoric diagenesis or dolomitization in our sample set, and published δ^13^C_carb_ values are robust and record globally correlatable trends. Given the close relationship of trends in the published δ^13^C_carb_ data and our new I/(Ca + Mg) values, it is highly likely that the I/(Ca + Mg) values are recording genuine changes in oxygen concentration. Furthermore, the use of a multi-proxy approach has allowed us to cross-reference I/(Ca + Mg) values with other proxy data, such as redox-sensitive trace metal EFs, which yield consistent results. Notwithstanding possible diagenetic effects, major increases and decreases in I/(Ca + Mg) values are, however, robust indicators of elevated and depleted oxygen concentrations in seawater. Here, we interpret our data as such. We do not include the commonly reported sub-oxic-dysoxic threshold of 0.5 μmol/mol and instead refer to values between 0 and 2.6 as “dysoxic” (i.e., with limited oxygen availability).

## Supplementary Materials

### This PDF file includes:

Supplementary Text Figs. S1 to S6 Tables S1 to S7 References
