# Supplementary material for: Low oxygen but dynamic marine redox conditions permitted the Cambrian Radiation
Source: Sci Adv. 2025 Jan 24;11(4):eads2846. doi: 10.1126/sciadv.ads2846 (PMC11759046; doi:10.1126/sciadv.ads2846)
Supplement: Supplementary file 1 — Supplementary Text Figs. S1 to S6 Tables S1 to S7 References [file sciadv.ads2846_sm.pdf]

Science Advances
AAAS

# Supplementary Materials for

## Low oxygen but dynamic marine redox conditions permitted the Cambrian Radiation

Ruaridh D. Alexander *et al.*

Corresponding author: Ruaridh D. Alexander, Ruaridh.Alexander@ed.ac.uk

*Sci. Adv.* **11**, eads2846 (2025)
DOI: 10.1126/sciadv.ads2846

**This PDF file includes:**

Supplementary Text
Figs. S1 to S6
Tables S1 to S7
References

## The Siberian Platform

The Siberian Platform was a vast, shallow-marine Ediacaran-Cambrian platform composed of multiple isolated carbonate platforms, or banks, within an epicontinental sea on the Siberian Craton, which is divided into distinct regions, defined by regional tectonic association or geographic area (Fig. 1). The platform preserves inner basin carbonate-clastic, transitional, and open-marine carbonate facies which deepened over time – open marine carbonate facies of the Tommotian and Atdabanian stages were replaced by deposition of organic-rich carbonates and shales from the Botoman Stage (*27, 34*). According to tectonic and paleomagnetic models, the Siberian Craton was tropical, located between the equator and 30°S, and was rotated ~180° relative to its modern position (*47*).

Successions on the Siberian Platform are typified by a diverse assemblage of SSFs, which appear close to the base of the Cambrian, although some anabaritids can be found in Ediacaran units in the Ust'-Yudoma Formation at the Kyra-Ytyga River section on the Yudoma-Maya belt (*100*). After the initial diversification of SSFs, reef-building archaeocyath sponges diversified, initially appearing in the lower Tommotian (upper Terraneuvian) in the Aldan-Lena region, before diversifying across the entire platform. In addition to SSFs and Archaeocyaths, trilobites made their first appearance globally in the body fossil record in the Lower Atdabanian (lower Stage 3) (ca. 521 Ma) (*27*), and are found in a range of habitats from shallow inner basin to open marine facies (*31*).

During the $\delta^{13}C_{carb}$ cycle IV interval (ca. 521–520 Ma, early Atdabanian or Stage 3) the Siberian fauna achieved its first acme in diversity, abundance and individual animal size (*19, 20*). Trilobites, possible lobopodianss (mostly preserved as *Rhombocorniculum*) and possible arthropods (*Mongolitubulus*-type ornamented rear spines of carapaces), representing highly motile animals, and thick-valved rhynchonelliform brachiopods became common elements of Siberian marine communities (*29, 31, 101*). The number of occurrences reduced to 38 but they became more evenly distributed within the Siberian Platform embracing the Anabar-Lena carbonate bank and the vast Turukhansk-Irkutsk-Olekma inner basin. By the end of this interval, with the appearance of corynexochoids (*Malykania*), redlichioids (*Elganellus*), hebediscids (*Delgadella*) and ellipsocephaloids (*Triangullina*) during the *Delgadella* Zone, trilobites expanded into calcareous-dolomitic muds of the inner Turukhansk-Irkutsk-Olekma basin (corynexochioids, redlichioids) and to argillaceous-calcareous muds of the deeper Yudoma-Olenek and Khantayka-Olenek basins (hebediscids, ellipsocephaloids) and three different trilobite communities were formed (*31*).

The three areas of the Siberian Platform under consideration include the Sukharikha River section on the lower Enisey River (Krasny Porog Formation, Igarka carbonate bank), the Ulakhan-Ald'yarkhay section on the lower Lena River (Tyuser Formation, Bulkur anticline, Anabar-Lena carbonate platform), and the Selinde River section in the Aldan River basin (Pestrotsvet Formation, Aldan-Lena carbonate platform) (Fig. 2).

The sites characterised by a high abundance of fossils were restricted to the shallowest facies of the carbonate banks of the Aldan-Lena, Daldyn-Markha, Igarka, Kotuy and Anabar-Lena areas, representing shallow water depths within fair-weather wave base – storm wave base (*27*). Facies include cross-bedded grainstones and wackestones, calcimicrobial buildups, and, in places, sandstones (*30, 102, 103, 104, 105, 106*). Rare SSFs (excluding protoconodonts) occur in shallow-water facies of the Turukhan-Irkutsk-Olekma area adjacent to the Daldyn-Markha bank and in some localities of the Yudoma-Olenek and Khantayka-Olenek basins, which did not differ significantly in terms of water depth from carbonate banks during that time (*34, 107, 108*).

The three sections studied herein were restricted to wholly shallow shelf, subtidal environments above storm wave base, with episodic storm reworking, and are located on the shelf break to upper slope of the Siberian Platform. Ulakhan-Ald'yarkhay represents the shallowest section. Bed 5 probably accumulated under very shallow water as the mudstone is silty, and succeeds (lowermost bed 5, not sampled here) a fine-grained, micaceous-quartzose siltstone. Massive calcimicrobial-archaeocyath reefs flanked by both argillaceous mudstones and grainstones follow (bed 6), also indicative of shallow water conditions. Reefs are followed by wavy-bedded argillaceous mudstone interbedded with glauconitic grainstones (bed 7). The succession therefore represents an overall deepening sequence.

Selinde (beds 37–54) also represents a shallow water succession. Here, there are many calcimicrobial reefs and calcimicrobial-archaeocyath reefs, although archaeocyaths were not as abundant and diverse as in the Aldan-Lena area, indicating slightly (ca. < 5 metres) deeper conditions. Burrows and glauconitic accumulations mark intervals of relatively low energy, the latter after reworking. Grainstones and floatstones flanking the reefs indicate episodic storm reworking. The section also shows evidence of deepening of the local basin above the reef massive (beds 48–54).

The Sukharikha section lacks archaeocyaths or reefal buildups in the interval studied (the middle Krasny Porog Formation), probably indicative of deeper conditions than the other studied sections during the same time interval. Beds are massively bedded but with thin-laminations, dominated by argillaceous mudstones and extremely rare fossils. A diverse

complex of archaeocyaths with six species of the *Nochoroicyathus sunnaginicus* Biozone was, however, reported from the uppermost 2 m of the Sukharikha Formation (*109*). Subsequent publications by other workers (*165, 110, 111*) refer to this initial report, but did not confirm the presence of archaeocyaths. Archaeocyaths are known from the lower 20 m of the Krasnoporog Formation in the Sukharikha section (*110*).

**Biotic distribution and preservational criteria**

Fossils were attributed to three groups according to their preservation. The first group includes skeletons of *in situ* reefal fauna (mostly archaeocyaths) preserved in life position. The second comprises slightly reworked well-preserved cephalons (trilobites) and shells (molluscs, hyoliths, brachiopods), which accumulated in mudstone without traces of either transportation or secondary mineralogical replacement. The third group holds various re-worked small shelly fossils with features of dissolution and secondary replacement of moulds by glauconite infilling shell chambers and formation of outer and/or inner phosphatic crusts. These fossils commonly form packstone and grainstone in thin layers and erosional pockets. Probable primary phosphatic shells (tommotiids, hyolithelminthes, protoconodonts, mobergellans) are ascribed to the third group because they always co-occur with fossils of this group of the same size range and have shells durable enough to resist both abrasion and replacement.

105 species of SSFs, archaeocyaths and trilobites are documented at Selinde. Of these, eight (three archaeocyath and five trilobite species) are considered to be definitively *in-situ* as they form, or are found within, an Archaeocyath bioherm in life position (Fig. S1). Other species are either re-worked or slightly re-worked and are preserved as calcareous or phosphatic skeletons.

Trilobites are represented, by well-preserved intact calcareous cephalons, only in the Selinde and Ulakhan-Ald’yarkhay sections. [They are absent in the studied interval of the Sukharikha section, but appear higher in the stratihgraphy in the uppermost strata of the Krasny Porog Formation (*Judomia/Uktaspis (Prouktaspis)* trilobite Zone)]. Trilobites are restricted either to pelitomorphic lenticular limestone within calcimicrobial-archaeocyath reefs (Ulakhan-Ald’yarkhay; *32, 164*) or to argillaceous dolomitic mudstone yielding minor calcimicrobial reefs and shelly-glauconitic grainstone (Selinde; *30*). Due to their preservation and a lack of features indicative of transportation, these fossils are interpreted here as slightly reworked, with the possible exception of the uppermost trilobites from Selinde. During the cycle IV interval, a similar succession of trilobites is observed in both sections as well as on the middle Lena River: the succession starts with fallotaspidoids *Profallotaspis* (vicariants *P.*

*jakutensis*, *P. tyusserica* and *P. privica*) at the rise of the cycle IV, followed by *Repinaella* (*R. explicata* and *R. sibirica*) from the same superfamily at the peak of the cycle, and by a more diverse assemblage including *Nevadella effusa, Paranevadella subgroenlandica* (fallotaspidoids) and *Delgadella anabara* (hebediscids), and some others at the end of the cycle (*30, 32, 164, 112*). This succession served for the establishment of the *Profallotaspis jakutensis*, *Repinaella*, and *Delgadella anabara* trilobite zones on the Siberian Platform. Interestingly, the first trilobites (*Profallotaspis* spp.) are always restricted to calcimicrobial-archaeocyath and pure calcimicrobial reefal facies of carbonate platforms and banks. The majority of the earliest trilobites belong to the Fallotaspidoidea superfamily. .

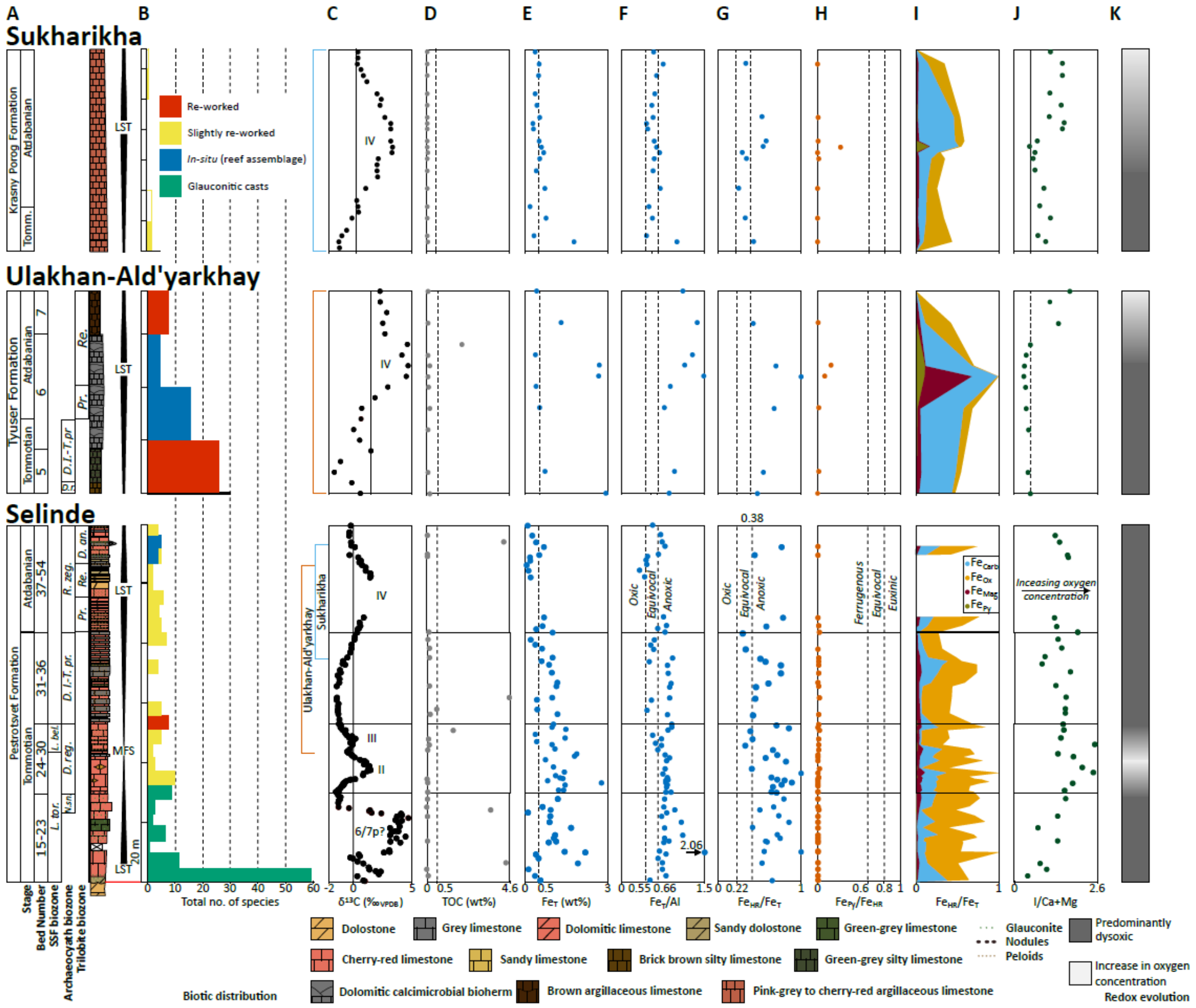

**Figure S1: Summary of redox and biotic distribution data. A:** Siberian time scale and biozone subdivisions and stratigraphy (after *28*, *164-166*); **B:** Biotic distribution data (see table S6 and references therein); **C:** $\delta^{13}C$; **D:** TOC; **E:** $Fe_T$; **F:** $Fe_T/Al$; **G:** $Fe_{HR}/Fe_T$; **H:** $Fe_{py}/Fe_T$; **I:** Separated Fe sequential extraction data; **J:** I/(Ca+Mg); **K:** Redox evolution.

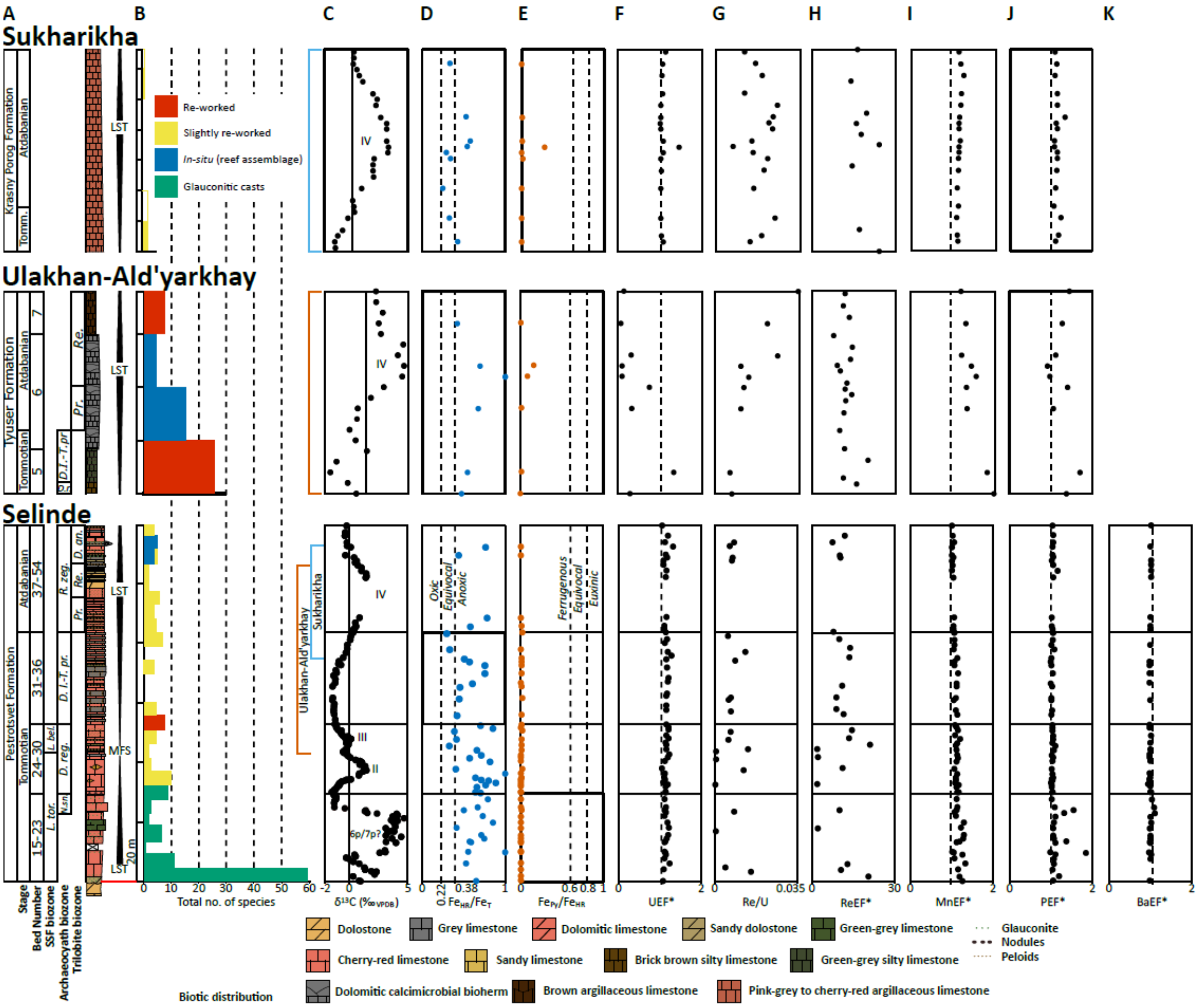

**Figure S2: Summary of Fe speciation, element, and biotic distribution data. A:** Siberian time scale and biozone subdivisions (after *28, 164-166*); **B:** Biotic distribution data (see table S6 and references therein); **C:** $\delta^{13}C$; **D:** $Fe_{HR}/Fe_T$; **E:** $Fe_{Py}/Fe_T$; **F:** UEF*; **G:** Re/U; **H:** ReEF*; **I:** MnEF*; **J:** PEF*; **K:** BaEF*.

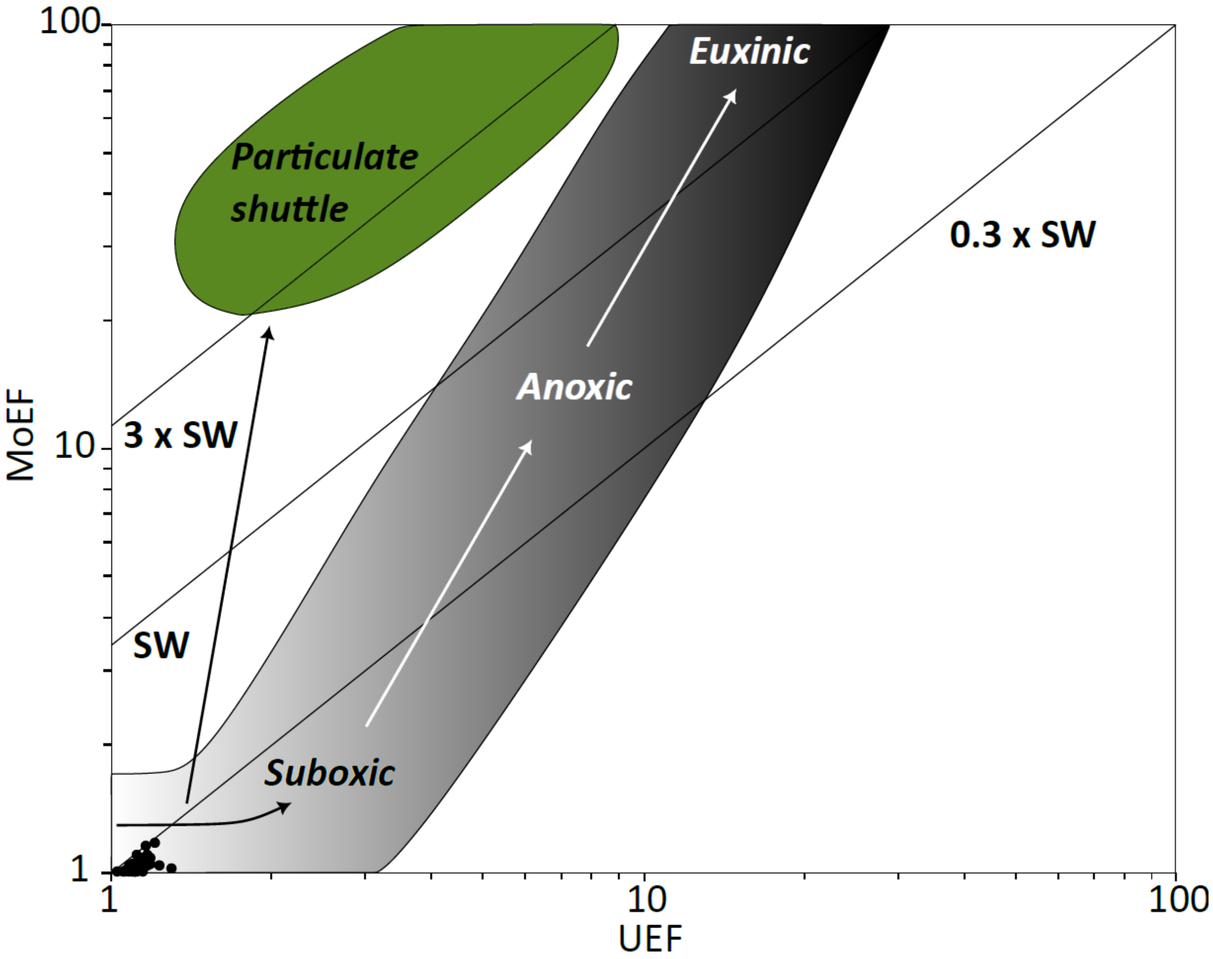


**Figure S3: Selinde MoEF* and UEF* relationships.** After (*40*).

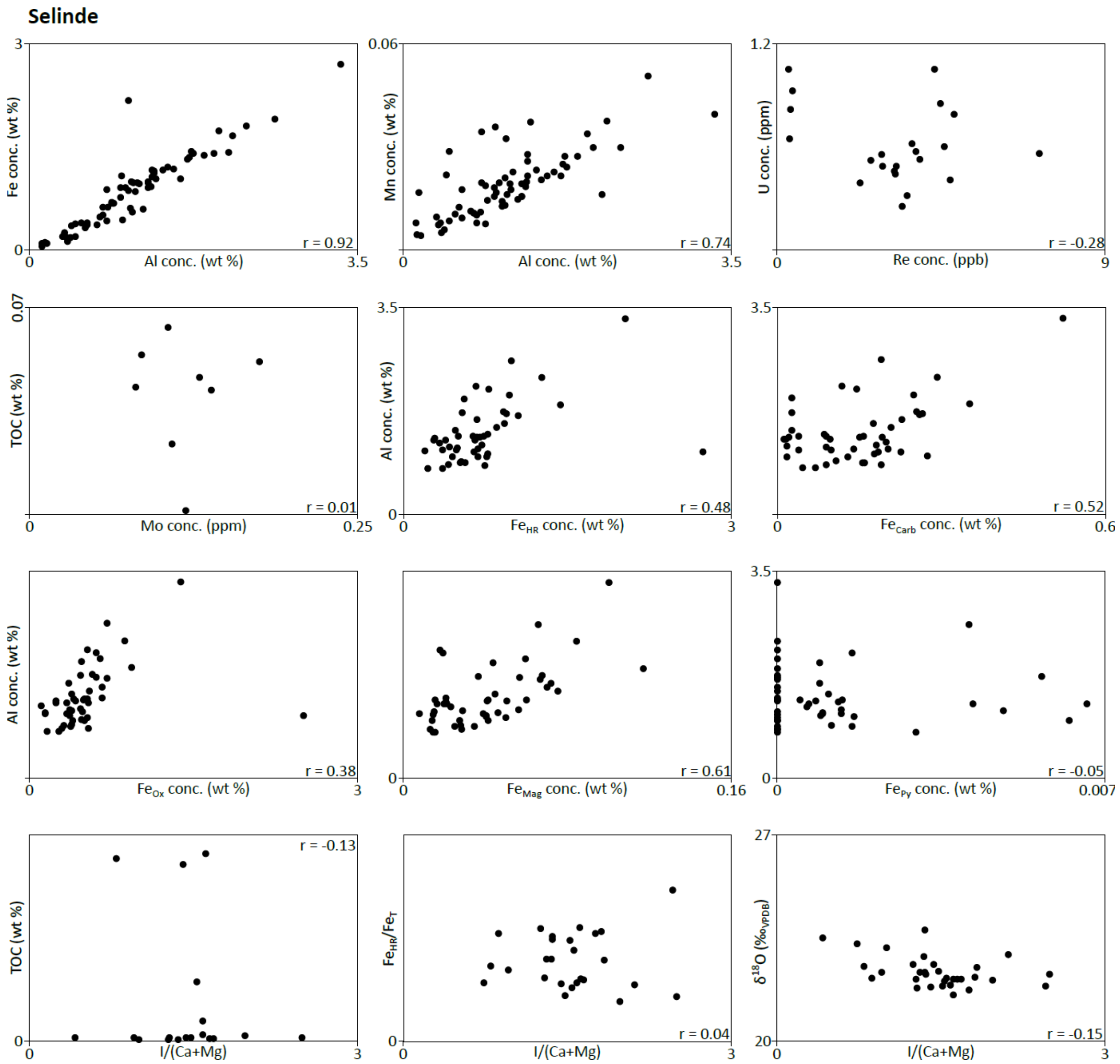


**Figure S4: Relationships of all elemental and isotopic data for Selinde.**

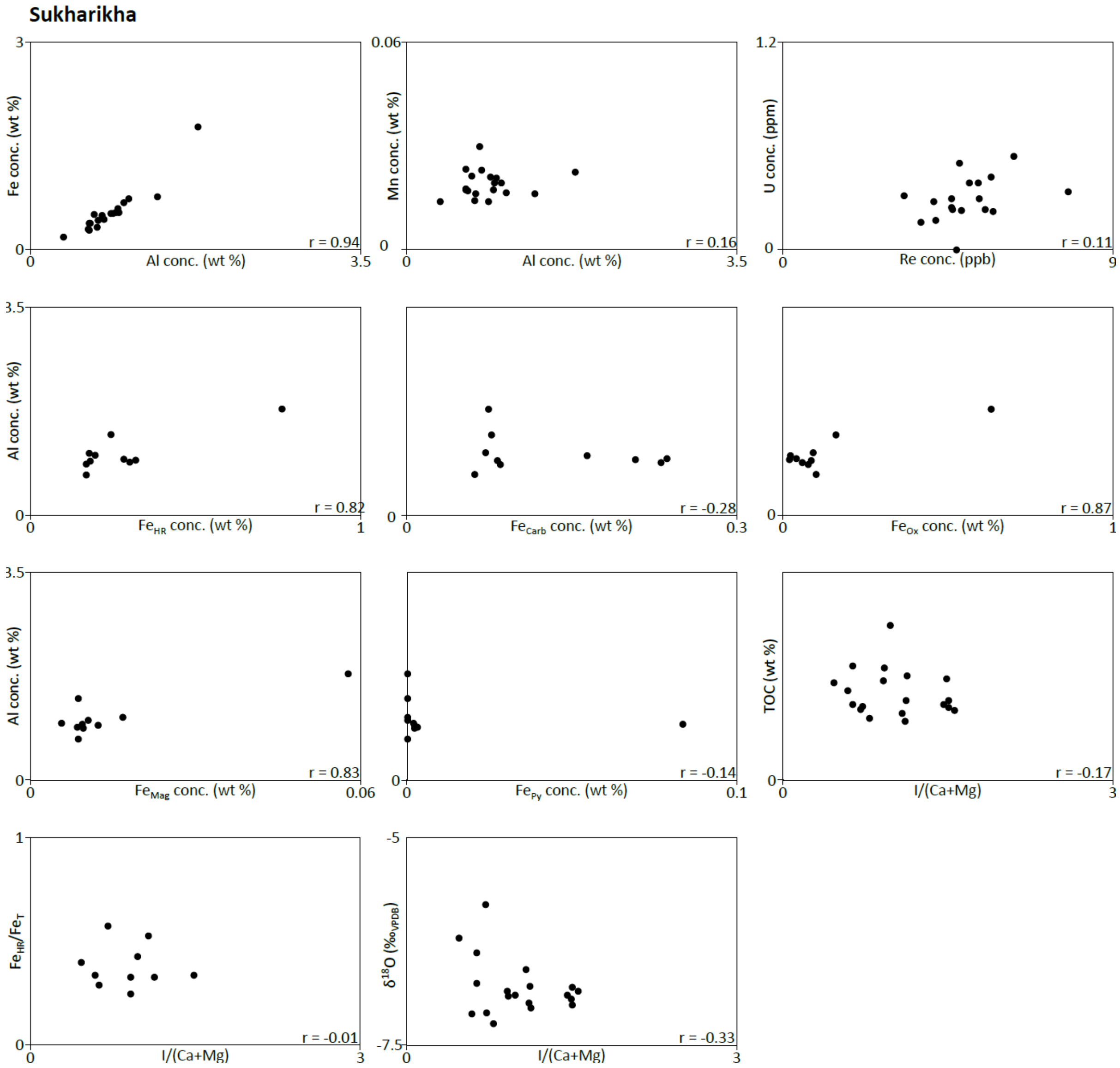


**Figure S5: Relationships of all elemental and isotopic data for Sukharikha.**

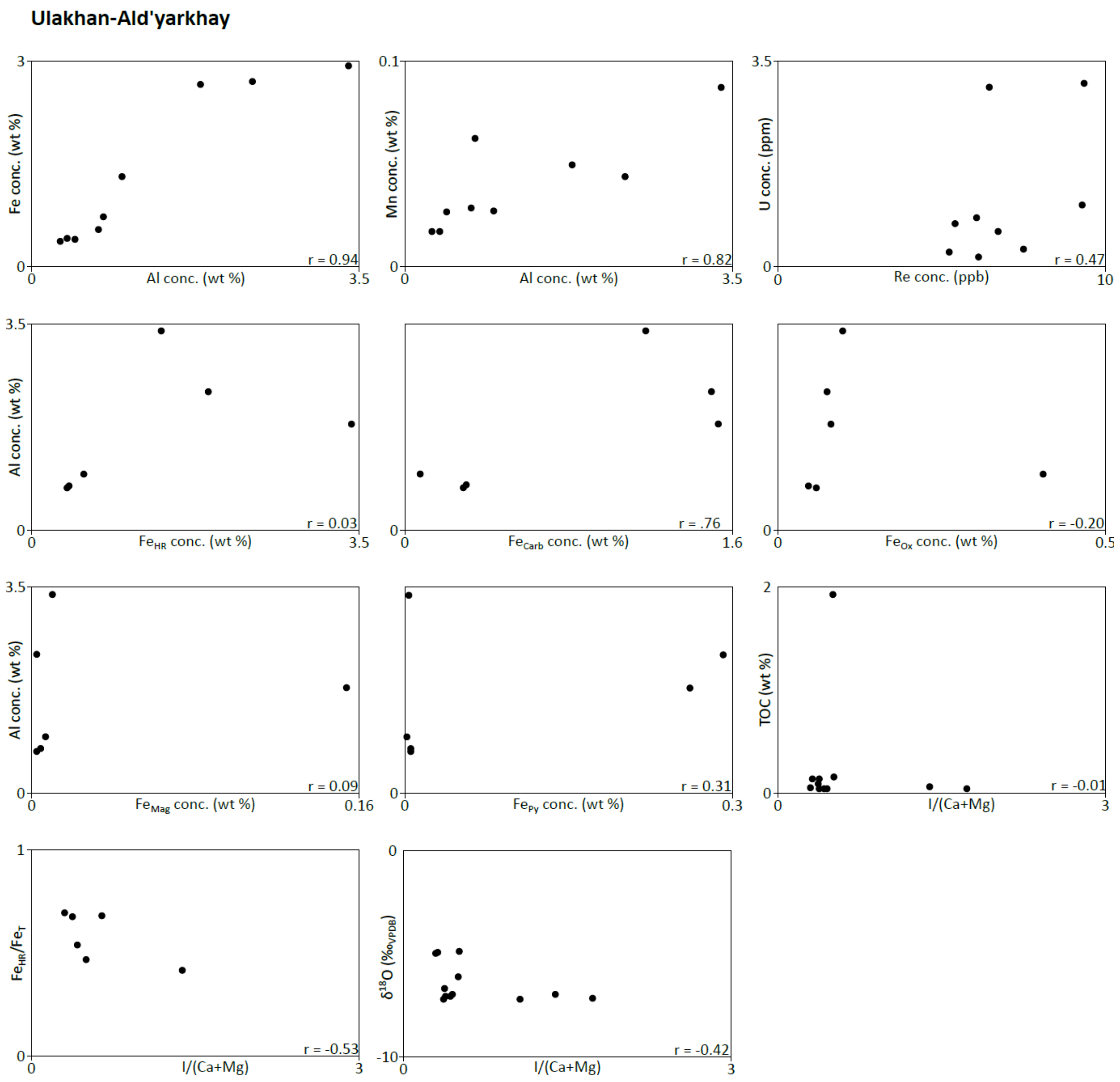


**Figure S6: Relationships of all elemental and isotopic data for Ulakhan-Ald'yarkhay.**

## Table S1: Major element data

| Sample ID | Position (m) | Age (Ma) | TOC (wt%) | Al (wt%) | Ca (wt%) | Mg (wt%) | Fe (wt%) | K (wt%) | Mn (wt%) | MnEF* | P (wt%) | PEF* | Ti (wt%) |
|---|---|---|---|---|---|---|---|---|---|---|---|---|---|
| | | | | | | Selinde | | | | | | | |
| 2 | 0.40 | 527.05 | | 1.10 | 21.27 | | 0.56 | 0.70 | 0.03 | 1.25 | 0.02 | 1.04 | 0.06 |
| 3 | 1.70 | 526.93 | 0.08 | 0.45 | 18.53 | 10.96 | 0.36 | | 0.02 | 1.19 | 0.04 | 1.18 | |
| 6 | 3.70 | 526.73 | 0.03 | 0.17 | 32.96 | 0.43 | 0.12 | | 0.02 | 1.16 | 0.01 | 1.03 | |
| 9 | 5.50 | 526.56 | 4.43 | 2.61 | 15.50 | 7.60 | 1.92 | | 0.05 | 1.33 | 0.05 | 1.06 | |
| 11 | 7.00 | 526.41 | | 0.75 | 41.62 | | 0.49 | 0.57 | 0.01 | 1.06 | 0.02 | 1.07 | 0.06 |
| 14 | 8.20 | 526.30 | | 0.49 | 32.44 | | 0.40 | 0.39 | 0.03 | 1.26 | 0.03 | 1.09 | 0.03 |
| 15 | 8.80 | 526.24 | | 1.06 | 33.56 | | 2.18 | 0.74 | 0.01 | 1.06 | 0.18 | 1.85 | 0.04 |
| 16 | 9.00 | 526.22 | | 2.12 | 29.32 | | 1.44 | 1.46 | 0.02 | 1.02 | 0.03 | 0.99 | 0.11 |
| 18 | 11.80 | 525.95 | | 1.13 | 35.39 | | 0.86 | 0.79 | 0.02 | 1.12 | 0.04 | 1.10 | 0.06 |
| 19 | 12.10 | 525.92 | 0.06 | 1.33 | 36.02 | 1.35 | 1.13 | 0.96 | 0.03 | 1.19 | 0.09 | 1.36 | 0.06 |
| 20 | 13.10 | 525.82 | | 1.35 | 29.90 | | 1.05 | 1.07 | 0.04 | 1.28 | 0.03 | 1.06 | 0.11 |
| 31 | 14.00 | 525.73 | | 0.98 | 30.19 | | 1.09 | 0.72 | 0.04 | 1.29 | 0.02 | 1.05 | 0.07 |
| 35 | 16.20 | 525.52 | | 2.17 | 21.40 | 3.27 | 1.67 | 1.77 | 0.04 | 1.23 | 0.04 | 1.05 | 0.16 |
| 37 | 17.90 | 525.36 | | 0.83 | 29.73 | | 0.89 | 0.68 | 0.03 | 1.29 | 0.02 | 1.06 | 0.07 |
| 41 | 19.70 | 525.18 | 0.05 | 1.26 | 35.79 | 0.76 | 0.92 | 0.96 | 0.02 | 1.11 | 0.04 | 1.10 | 0.10 |
| 45 | 20.80 | 525.08 | | 0.13 | 50.78 | | 0.11 | 0.09 | 0.01 | 1.07 | 0.06 | 1.31 | 0.01 |
| 47 | 21.60 | 525.00 | 3.55 | 0.97 | 27.42 | 1.64 | 0.93 | | 0.02 | 1.12 | 0.12 | 1.55 | |
| 49 | 22.50 | 524.65 | 0.02 | 0.84 | 37.77 | | 0.64 | 0.62 | 0.02 | 1.14 | 0.03 | 1.09 | 0.08 |
| 53 | 24.90 | 524.29 | 0.04 | 1.54 | 35.59 | 1.09 | 1.19 | 1.23 | 0.02 | 1.11 | 0.03 | 1.02 | 0.14 |
| 57 | 27.30 | 523.92 | | 1.71 | 29.06 | | 1.36 | 1.31 | 0.03 | 1.14 | 0.03 | 1.04 | 0.16 |
| 58 | 27.40 | 523.91 | 0.05 | 1.33 | 34.29 | 0.76 | 1.17 | 0.96 | 0.03 | 1.17 | 0.03 | 1.04 | 0.14 |
| 62 | 30.00 | 523.51 | | 1.74 | 30.07 | | 1.42 | 1.28 | 0.02 | 1.12 | 0.03 | 1.02 | 0.17 |
| 65(2) | 31.00 | 523.36 | | 3.32 | 15.14 | 4.57 | 2.72 | 2.35 | 0.04 | 1.17 | 0.05 | 1.01 | 0.28 |
| 67(2) | 32.00 | 523.21 | 0.04 | 0.97 | 40.43 | | 0.78 | 0.73 | 0.02 | 1.09 | 0.01 | 1.00 | 0.08 |
| 69 | 33.10 | 523.04 | | 1.68 | 32.35 | | 1.34 | 1.33 | 0.02 | 1.10 | 0.03 | 1.04 | 0.17 |
| 71 | 33.60 | 522.97 | 0.00 | 1.47 | 35.23 | | 1.22 | 1.11 | 0.02 | 1.11 | 0.02 | 1.02 | 0.16 |
| 72 | 34.50 | 522.93 | | 1.30 | 34.33 | | 1.08 | 1.13 | 0.02 | 1.10 | 0.03 | 1.05 | 0.19 |
| 74 | 36.40 | 522.84 | | 1.86 | 27.85 | 4.21 | 1.39 | 1.56 | 0.03 | 1.15 | 0.03 | 1.03 | 0.20 |
| 77 | 38.80 | 522.73 | | 1.27 | 30.38 | 0.52 | 0.99 | | 0.02 | 1.07 | 0.02 | 1.01 | |
| 82 | 42.50 | 522.56 | | 0.87 | 38.29 | | 0.71 | 0.66 | 0.02 | 1.13 | 0.02 | 1.03 | 0.08 |
| 84 | 44.20 | 522.48 | | 2.02 | 26.80 | 1.99 | 1.74 | 1.72 | 0.03 | 1.16 | 0.04 | 1.05 | 0.28 |
| 86 | 45.50 | 522.42 | | 2.32 | 18.60 | 5.55 | 1.82 | 1.88 | 0.03 | 1.14 | 0.04 | 1.02 | 0.24 |
| 89 | 48.00 | 522.31 | 0.05 | 1.61 | 25.29 | | 1.05 | 1.56 | 0.02 | 1.12 | 0.04 | 1.07 | 0.19 |
| 90 | 50.20 | 522.20 | 0.10 | 1.30 | 23.89 | 1.74 | 0.93 | | 0.02 | 1.11 | 0.04 | 1.09 | |
| 92 | 51.30 | 522.15 | | 0.62 | 44.47 | | 0.38 | 0.48 | 0.02 | 1.14 | 0.01 | 1.02 | 0.07 |
| 95 | 53.30 | 522.06 | 0.09 | 1.96 | 16.90 | 5.49 | 1.41 | | 0.03 | 1.21 | 0.04 | 1.05 | |
| 98 | 54.90 | 521.99 | | 0.60 | 40.11 | | 0.33 | 0.52 | 0.01 | 1.09 | 0.02 | 1.04 | 0.07 |
| 101 | 57.40 | 521.87 | 1.45 | 1.73 | 22.22 | 2.51 | 1.45 | | 0.03 | 1.16 | 0.03 | 1.04 | |
| 103 | 58.80 | 521.81 | | 1.02 | 38.34 | | 0.93 | 0.79 | 0.02 | 1.13 | 0.02 | 1.03 | 0.14 |
| 105 | 60.20 | 521.74 | | 1.11 | 37.63 | 0.66 | 1.00 | 0.90 | 0.02 | 1.09 | 0.02 | 1.04 | 0.14 |

| Sample ID | Position (m) | Age (Ma) | TOC (wt%) | Al (wt%) | Ca (wt%) | Mg (wt%) | Fe (wt%) | K (wt%) | Mn (wt%) | MnEF* | P (wt%) | PEF* | Ti (wt%) |
|---|---|---|---|---|---|---|---|---|---|---|---|---|---|
| 109 | 63.20 | 521.61 | 0.15 | 1.42 | 25.92 | 1.84 | 1.18 | | 0.02 | 1.14 | 0.02 | 1.03 | |
| 111 | 64.50 | 521.55 | 0.49 | 0.99 | 31.23 | 1.10 | 0.45 | | 0.02 | 1.10 | 0.02 | 1.04 | |
| 114 | 67.20 | 521.42 | | 0.82 | 43.83 | | 0.44 | 0.60 | 0.01 | 1.06 | 0.01 | 1.01 | 0.07 |
| 115 | 67.80 | 521.40 | 4.56 | 1.15 | 30.75 | 1.33 | 0.99 | | 0.02 | 1.10 | 0.02 | 1.02 | |
| 116 | 71.00 | 521.25 | 0.08 | 1.33 | 24.83 | 3.04 | 1.14 | | 0.02 | 1.13 | 0.03 | 1.04 | |
| 117 | 72.00 | 521.21 | | 1.31 | 32.28 | | 1.18 | 0.98 | 0.02 | 1.11 | 0.02 | 1.01 | 0.13 |
| 120 | 74.80 | 521.12 | | 1.17 | 34.74 | 2.20 | 0.98 | 0.88 | 0.02 | 1.15 | 0.03 | 1.06 | 0.11 |
| 122 | 77.00 | 521.04 | | 1.05 | 31.79 | 3.56 | 0.87 | 0.76 | 0.01 | 1.07 | 0.02 | 1.03 | 0.10 |
| 124 | 78.00 | 521.00 | | 1.22 | 35.23 | | 0.60 | 0.88 | 0.02 | 1.07 | 0.02 | 1.00 | 0.09 |
| 125 | 79.00 | 520.97 | 0.09 | 1.09 | 23.91 | 3.67 | 1.00 | | 0.02 | 1.14 | 0.02 | 1.02 | |
| 127 | 81.50 | 520.88 | 0.08 | 1.08 | 29.39 | 1.05 | 0.62 | | 0.01 | 1.06 | 0.03 | 1.08 | |
| 128 | 82.60 | 520.84 | | 0.72 | 43.29 | | 0.38 | 0.59 | 0.01 | 1.07 | 0.01 | 1.01 | 0.06 |
| 129 | 84.00 | 520.79 | 0.04 | 0.36 | 33.89 | 0.70 | 0.21 | | 0.01 | 1.07 | 0.01 | 1.05 | |
| 131 | 86.00 | 520.73 | 0.15 | 1.27 | 26.37 | 1.81 | 1.00 | | 0.02 | 1.07 | 0.03 | 1.05 | |
| 132 | 87.00 | 520.69 | | 0.62 | 42.54 | | 0.41 | 0.49 | 0.01 | 1.05 | 0.02 | 1.03 | 0.05 |
| 134 | 87.90 | 520.66 | | 0.78 | 43.99 | 1.61 | 0.64 | 0.63 | 0.01 | 1.05 | 0.01 | 1.01 | 0.07 |
| 136 | 90.50 | 520.57 | | 0.90 | 36.72 | 3.53 | 0.69 | 0.77 | 0.01 | 1.08 | 0.02 | 1.02 | 0.08 |
| 137 | 102.50 | 519.80 | | 0.49 | 47.42 | | 0.21 | 0.42 | 0.01 | 1.05 | 0.02 | 1.04 | 0.03 |
| 140 | 104.50 | 519.71 | | 0.40 | 45.48 | | 0.13 | 0.36 | 0.01 | 1.03 | 0.03 | 1.14 | 0.02 |
| 142 | 106.20 | 519.63 | | 0.14 | 50.77 | | 0.06 | 0.11 | 0.00 | 1.04 | 0.01 | 1.05 | 0.01 |
| 144 | 107.50 | 519.57 | | 0.44 | 52.54 | | 0.20 | 0.39 | 0.01 | 1.03 | 0.01 | 1.01 | 0.03 |
| 145 | 108.50 | 519.53 | 0.06 | 0.39 | 33.71 | 0.49 | 0.19 | | 0.01 | 1.05 | 0.01 | 1.04 | |
| 146 | 109.20 | 519.49 | 0.05 | 0.78 | 33.69 | 0.77 | 0.52 | | 0.01 | 1.03 | 0.02 | 1.03 | |
| 147 | 111.60 | 519.38 | | 0.88 | 41.93 | | 0.69 | 0.70 | 0.01 | 1.02 | 0.02 | 1.02 | 0.06 |
| 149 | 113.00 | 519.32 | 4.30 | 0.55 | 32.22 | 0.53 | 0.41 | | 0.01 | 1.07 | 0.02 | 1.05 | |
| 150 | 115.00 | 519.23 | 0.03 | 0.37 | 34.10 | 0.57 | 0.27 | | 0.01 | 1.05 | 0.01 | 1.05 | |
| 152 | 118.00 | 519.09 | | 0.18 | 58.01 | | 0.10 | 0.12 | 0.00 | 1.03 | 0.01 | 1.02 | 0.01 |
| **Sukharikha** | | | | | | | | | | | | | |
| 356 | 587.6 | 521.27 | 0.05 | 0.86 | 30.30 | 0.24 | 0.52 | | 0.01 | 1.08 | 0.02 | 1.10 | |
| 358 | 589.6 | 521.16 | 0.07 | 1.78 | 25.49 | 0.32 | 1.79 | | 0.02 | 1.10 | 0.04 | 1.11 | |
| 359 | 590.6 | 521.11 | 0.04 | 0.72 | 30.11 | 0.25 | 0.32 | | 0.01 | 1.09 | 0.03 | 1.17 | |
| 362 | 593.6 | 520.95 | 0.05 | 1.36 | 30.77 | 0.27 | 0.76 | | 0.02 | 1.07 | 0.04 | 1.23 | |
| 364 | 595.6 | 520.84 | 0.03 | 0.36 | 43.31 | 0.32 | 0.18 | | 0.01 | 1.11 | 0.01 | 1.06 | |
| 367 | 598.6 | 520.68 | 0.05 | 1.05 | 21.73 | 0.29 | 0.73 | | 0.02 | 1.09 | 0.02 | 1.10 | |
| 370 | 601.6 | 520.51 | 0.04 | 0.73 | 30.14 | 0.56 | 0.42 | | 0.02 | 1.11 | 0.02 | 1.13 | |
| 372 | 603.6 | 520.41 | 0.04 | 0.92 | 28.56 | 0.48 | 0.54 | | 0.02 | 1.11 | 0.03 | 1.15 | |
| 373 | 604.6 | 520.35 | 0.05 | 1.00 | 28.77 | 1.22 | 0.69 | | 0.02 | 1.13 | 0.03 | 1.15 | |
| 374 | 605.6 | 520.30 | 0.05 | 0.93 | 27.09 | 2.11 | 0.60 | | 0.02 | 1.13 | 0.02 | 1.07 | |
| 375 | 606.6 | 520.24 | 0.03 | 0.89 | 28.27 | 2.61 | 0.52 | | 0.02 | 1.15 | 0.02 | 1.08 | |
| 377 | 608.6 | 520.14 | 0.04 | 0.62 | 31.52 | 0.28 | 0.30 | | 0.02 | 1.13 | 0.02 | 1.14 | |
| 378 | 609.6 | 520.08 | 0.03 | 0.63 | 31.66 | 0.28 | 0.29 | | 0.02 | 1.13 | 0.02 | 1.12 | |
| 379 | 610.6 | 520.03 | 0.03 | 0.95 | 21.39 | 1.02 | 0.54 | | 0.02 | 1.14 | 0.05 | 1.33 | |
| 381 | 612.6 | 519.92 | 0.04 | 0.79 | 29.90 | 0.25 | 0.44 | | 0.02 | 1.18 | 0.03 | 1.14 | |
| 383 | 614.6 | 519.81 | 0.03 | 0.63 | 30.71 | 0.22 | 0.38 | | 0.02 | 1.19 | 0.02 | 1.15 | |

| Sample ID | Position (m) | Age (Ma) | TOC (wt%) | Al (wt%) | Ca (wt%) | Mg (wt%) | Fe (wt%) | K (wt%) | Mn (wt%) | MnEF* | P (wt%) | PEF* | Ti (wt%) |
|---|---|---|---|---|---|---|---|---|---|---|---|---|---|
| 386 | 617.6 | 519.65 | 0.03 | 0.77 | 30.85 | 0.48 | 0.49 | | 0.03 | 1.25 | 0.02 | 1.09 | |
| 388 | 619.6 | 519.54 | 0.05 | 0.68 | 31.76 | 0.27 | 0.52 | | 0.02 | 1.17 | 0.02 | 1.13 | |
| 390 | 621.6 | 519.43 | 0.04 | 0.64 | 31.85 | 0.69 | 0.38 | | 0.02 | 1.13 | 0.02 | 1.09 | |
| **Ulakhan-Ald'yarkhay** | | | | | | | | | | | | | |
| 21/31 | 31.00 | 521.74 | 0.16 | 3.38 | 13.53 | 3.33 | 2.93 | | 0.12 | 1.99 | 0.12 | 1.38 | |
| 21/33 | 33.00 | 521.42 | 0.05 | 0.76 | 33.57 | 0.42 | 0.73 | | 0.09 | 1.82 | 0.15 | 1.71 | |
| 21/37 | 37.00 | 520.92 | 0.05 | | 37.94 | 0.32 | | | | | | | |
| 21/39 | 39.00 | 520.80 | 0.14 | 0.71 | 35.79 | 0.76 | 0.55 | | 0.04 | 1.36 | 0.02 | 1.06 | |
| 21/41 | 41.00 | 520.57 | 0.09 | 0.46 | 36.21 | 0.49 | 0.40 | | 0.04 | 1.34 | 0.09 | 1.40 | |
| 21/42 | 42.00 | 520.41 | 0.06 | 1.80 | 17.46 | 7.09 | 2.66 | | 0.07 | 1.58 | 0.02 | 0.96 | |
| 21/43 | 43.00 | 520.24 | 0.15 | 2.35 | 18.27 | 7.57 | 2.70 | | 0.06 | 1.45 | 0.02 | 0.92 | |
| 21/44 | 44.00 | 520.08 | 0.05 | 0.30 | 37.99 | 0.56 | 0.38 | | 0.02 | 1.22 | 0.03 | 1.12 | |
| 21/45 | 45.00 | 519.92 | 1.93 | | 31.55 | 0.52 | | | | | | | |
| 21/47 | 47.00 | 519.66 | 0.07 | 0.96 | 35.53 | 0.27 | 1.31 | | 0.04 | 1.32 | 0.07 | 1.28 | |
| 21/49 | 49.00 | 519.54 | | | 35.35 | 0.32 | | | | | | | |
| 21/50 | 50.00 | 519.49 | 0.05 | 0.38 | 37.55 | 0.26 | 0.42 | | 0.02 | 1.21 | 0.09 | 1.44 | |

**Table S2: Trace element data. BD = Below Detection.**

| Sample ID | Ba (wt%) | BaEF* | V (ppm) | VEF* | Cr (ppm) | CrEF* | Cu (ppm) | CuEF* | Zn (ppm) | ZnEF* | Mo (ppm) | MoEF* |
|---|---|---|---|---|---|---|---|---|---|---|---|---|
| | | | | | **Selinde** | | | | | | | |
| 2 | 0.003 | 0.97 | 17.53 | 1.16 | 14.91 | 1.21 | 1.60 | 1.00 | 9.09 | 1.05 | 0.09 | 1.00 |
| 3 | | | 14.73 | 1.17 | 8.18 | 1.12 | 5.07 | 1.22 | 9.96 | 1.11 | | |
| 6 | | | 5.58 | 1.06 | 3.95 | 1.06 | 1.49 | 1.06 | 10.99 | 1.14 | | |
| 9 | | | 50.07 | 1.50 | 48.93 | 1.74 | 8.78 | 1.26 | 31.34 | 1.25 | | |
| 11 | 0.003 | 0.98 | 13.42 | 1.13 | 16.78 | 1.27 | 2.11 | 1.05 | 12.73 | 1.12 | 0.10 | 1.04 |
| 14 | 0.003 | 1.00 | 8.46 | 1.08 | 8.57 | 1.13 | 4.42 | 1.18 | 10.38 | 1.11 | 0.11 | 1.06 |
| 15 | 0.003 | 0.97 | 24.85 | 1.26 | 17.77 | 1.26 | 2.58 | 1.06 | 19.91 | 1.20 | 0.14 | 1.05 |
| 16 | 0.006 | 0.94 | 37.67 | 1.37 | 32.02 | 1.46 | 4.81 | 1.09 | 13.84 | 1.04 | 0.12 | 0.96 |
| 18 | 0.003 | 0.97 | 22.14 | 1.22 | 20.08 | 1.30 | 4.10 | 1.13 | 14.52 | 1.12 | 0.09 | 1.00 |
| 19 | 0.004 | 0.97 | 27.11 | 1.28 | 27.00 | 1.42 | 3.91 | 1.10 | 10.23 | 1.05 | 0.11 | 1.00 |
| 20 | 0.005 | 0.97 | 24.87 | 1.24 | 21.92 | 1.32 | 4.92 | 1.15 | 9.47 | 1.04 | 0.12 | 1.01 |
| 31 | 0.003 | 0.98 | 21.52 | 1.22 | 18.17 | 1.28 | 4.51 | 1.16 | 15.42 | 1.14 | 0.13 | 1.04 |
| 35 | 0.008 | 0.96 | 42.38 | 1.43 | 41.05 | 1.63 | 6.67 | 1.18 | 13.13 | 1.03 | 0.16 | 0.99 |
| 37 | 0.003 | 0.99 | 14.92 | 1.15 | 13.97 | 1.21 | 4.67 | 1.17 | 14.15 | 1.14 | 0.11 | 1.04 |
| 41 | 0.004 | 0.97 | 29.21 | 1.31 | 27.54 | 1.43 | 3.06 | 1.07 | 17.36 | 1.15 | 0.09 | 0.99 |
| 45 | 0.007 | 1.08 | 4.30 | 1.05 | 5.76 | 1.10 | 1.55 | 1.07 | 6.06 | 1.07 | 0.11 | 1.09 |
| 47 | | | 22.69 | 1.24 | 16.75 | 1.25 | 7.97 | 1.33 | 29.49 | 1.34 | | |
| 49 | 0.009 | 1.06 | 21.44 | 1.23 | 20.19 | 1.32 | 6.84 | 1.28 | 20.35 | 1.22 | 0.11 | 1.04 |
| 53 | 0.008 | 1.01 | 32.17 | 1.33 | 30.16 | 1.46 | 9.21 | 1.35 | 20.26 | 1.17 | 0.14 | 1.02 |
| 57 | 0.008 | 0.99 | 35.35 | 1.36 | 31.00 | 1.47 | 9.35 | 1.35 | 23.23 | 1.20 | 0.13 | 1.00 |
| 58 | 0.006 | 0.99 | 31.65 | 1.34 | 29.07 | 1.46 | 12.40 | 1.52 | 23.26 | 1.23 | 0.13 | 1.02 |
| 62 | 0.007 | 0.97 | 39.39 | 1.41 | 38.88 | 1.61 | 8.76 | 1.31 | 23.13 | 1.20 | 0.17 | 1.04 |
| 65(2) | 0.012 | 0.93 | 62.66 | 1.62 | 60.40 | 1.91 | 17.13 | 1.62 | 37.74 | 1.29 | 0.32 | 1.06 |
| 67(2) | 0.003 | 0.98 | 20.81 | 1.22 | 20.03 | 1.31 | 4.69 | 1.17 | 12.54 | 1.11 | 0.08 | 1.00 |
| 69 | 0.006 | 0.96 | 36.48 | 1.38 | 37.13 | 1.59 | 10.36 | 1.40 | 22.29 | 1.19 | 0.16 | 1.03 |
| 71 | 0.004 | 0.96 | 32.60 | 1.34 | 39.45 | 1.64 | 8.60 | 1.32 | 21.17 | 1.19 | 0.12 | 1.00 |
| 72 | 0.005 | 0.98 | 28.39 | 1.30 | 62.13 | 2.08 | 7.77 | 1.30 | 17.40 | 1.15 | 0.11 | 1.01 |

| Sample ID | Ba (wt%) | BaEF* | V (ppm) | VEF* | Cr (ppm) | CrEF* | Cu (ppm) | CuEF* | Zn (ppm) | ZnEF* | Mo (ppm) | MoEF* |
|---|---|---|---|---|---|---|---|---|---|---|---|---|
| 74 | 0.006 | 0.96 | 40.85 | 1.43 | 34.60 | 1.53 | 12.20 | 1.48 | 23.22 | 1.19 | 0.16 | 1.02 |
| 77 | | | 27.26 | 1.28 | 27.64 | 1.43 | 8.11 | 1.31 | 32.14 | 1.35 | | |
| 82 | 0.003 | 0.98 | 38.44 | 1.46 | 25.70 | 1.42 | 4.60 | 1.17 | 16.54 | 1.17 | 0.12 | 1.05 |
| 84 | 0.007 | 0.96 | 59.95 | 1.67 | 35.88 | 1.54 | 13.17 | 1.51 | 29.81 | 1.27 | 0.27 | 1.10 |
| 86 | 0.009 | 0.96 | 46.17 | 1.47 | 39.06 | 1.58 | 12.51 | 1.46 | 30.33 | 1.26 | 0.37 | 1.17 |
| 89 | 0.007 | 0.98 | 31.94 | 1.32 | 28.77 | 1.43 | 12.10 | 1.49 | 23.75 | 1.22 | 0.18 | 1.04 |
| 90 | | | 27.63 | 1.29 | 27.78 | 1.44 | 10.21 | 1.42 | 41.72 | 1.48 | | |
| 92 | 0.002 | 0.98 | 22.01 | 1.26 | 12.80 | 1.20 | 3.44 | 1.13 | 11.17 | 1.11 | 0.07 | 1.02 |
| 95 | | | 39.13 | 1.40 | 41.75 | 1.65 | 12.42 | 1.48 | 45.35 | 1.49 | | |
| 98 | 0.002 | 0.99 | 10.96 | 1.11 | 9.65 | 1.14 | 6.07 | 1.26 | 155.66 | 3.09 | 0.08 | 1.03 |
| 101 | | | 35.82 | 1.37 | 32.98 | 1.50 | 12.57 | 1.50 | 55.08 | 1.64 | | |
| 103 | 0.003 | 0.97 | 35.77 | 1.41 | 23.84 | 1.38 | 7.29 | 1.29 | 18.76 | 1.19 | 0.18 | 1.09 |
| 105 | 0.003 | 0.97 | 25.89 | 1.27 | 21.54 | 1.33 | 6.90 | 1.27 | 18.57 | 1.18 | 0.13 | 1.04 |
| 109 | | | 28.10 | 1.28 | 25.13 | 1.38 | 10.38 | 1.42 | 43.66 | 1.50 | | |
| 111 | | | 19.25 | 1.19 | 18.45 | 1.28 | 4.96 | 1.18 | 33.32 | 1.39 | | |
| 114 | 0.002 | 0.98 | 21.01 | 1.23 | 14.49 | 1.22 | 3.36 | 1.11 | 13.08 | 1.12 | 0.07 | 1.01 |
| 115 | | | 26.21 | 1.28 | 21.23 | 1.32 | 6.49 | 1.24 | 28.36 | 1.31 | | |
| 116 | | | 24.83 | 1.25 | 26.90 | 1.42 | 12.66 | 1.54 | 32.17 | 1.35 | | |
| 117 | 0.004 | 0.97 | 30.01 | 1.32 | 28.03 | 1.44 | 8.02 | 1.31 | 17.74 | 1.15 | 0.27 | 1.15 |
| 120 | 0.004 | 0.97 | 24.07 | 1.25 | 21.56 | 1.33 | 5.10 | 1.17 | 17.29 | 1.16 | 0.16 | 1.06 |
| 122 | 0.003 | 0.97 | 22.19 | 1.23 | 20.28 | 1.31 | 5.00 | 1.18 | 15.61 | 1.14 | 0.19 | 1.10 |
| 124 | 0.004 | 0.96 | 24.55 | 1.25 | 21.96 | 1.33 | 9.22 | 1.37 | 15.76 | 1.13 | 0.13 | 1.03 |
| 125 | | | 22.84 | 1.23 | 21.10 | 1.32 | 27.40 | 2.28 | 22.20 | 1.23 | | |
| 127 | | | 19.98 | 1.20 | 19.96 | 1.30 | 6.20 | 1.23 | 24.52 | 1.26 | | |
| 128 | 0.002 | 0.98 | 19.33 | 1.21 | 13.28 | 1.20 | 27.16 | 2.30 | 15.13 | 1.16 | 0.08 | 1.02 |
| 129 | | | 6.55 | 1.06 | 9.88 | 1.16 | 4.76 | 1.21 | 17.77 | 1.22 | | |
| 131 | | | 23.71 | 1.23 | 21.91 | 1.33 | 5.24 | 1.17 | 29.84 | 1.32 | | |
| 132 | 0.002 | 0.98 | 12.81 | 1.13 | 10.77 | 1.16 | 2.23 | 1.07 | 8.75 | 1.08 | 0.07 | 1.02 |
| 134 | 0.002 | 0.98 | 16.50 | 1.17 | 13.96 | 1.21 | 3.75 | 1.13 | 9.88 | 1.08 | 0.11 | 1.04 |

| Sample ID | Ba (wt%) | BaEF* | V (ppm) | VEF* | Cr (ppm) | CrEF* | Cu (ppm) | CuEF* | Zn (ppm) | ZnEF* | Mo (ppm) | MoEF* |
|---|---|---|---|---|---|---|---|---|---|---|---|---|
| 136 | 0.003 | 0.98 | 21.86 | 1.23 | 19.48 | 1.31 | 3.10 | 1.09 | 12.27 | 1.11 | 0.16 | 1.08 |
| 137 | 0.002 | 0.99 | 7.29 | 1.06 | 7.30 | 1.10 | 4.33 | 1.18 | 10.12 | 1.10 | 0.04 | 1.01 |
| 140 | 0.001 | 0.99 | 5.81 | 1.05 | 5.94 | 1.08 | 8.85 | 1.41 | 9.67 | 1.10 | 0.03 | 1.00 |
| 142 | 0.001 | 1.00 | 2.14 | 1.02 | 3.29 | 1.05 | 0.62 | 1.02 | 8.10 | 1.10 | 0.02 | 1.01 |
| 144 | 0.001 | 0.99 | 5.99 | 1.05 | 6.17 | 1.09 | 3.44 | 1.14 | 8.35 | 1.08 | 0.03 | 1.00 |
| 145 | | | 6.32 | 1.06 | 7.22 | 1.11 | 4.95 | 1.22 | 34.93 | 1.45 | | |
| 146 | | | 11.29 | 1.10 | 12.04 | 1.17 | 6.32 | 1.26 | 44.35 | 1.55 | | |
| 147 | 0.003 | 0.97 | 15.72 | 1.15 | 14.18 | 1.21 | 3.42 | 1.11 | 13.54 | 1.13 | 0.09 | 1.02 |
| 149 | | | 10.57 | 1.11 | 10.37 | 1.16 | 5.19 | 1.22 | 15.44 | 1.17 | | |
| 150 | | | 7.04 | 1.07 | 7.10 | 1.11 | 1.73 | 1.06 | 15.31 | 1.18 | | |
| 152 | 0.001 | 1.00 | 3.97 | 1.04 | 3.56 | 1.05 | 0.70 | 1.02 | 5.56 | 1.06 | 0.02 | 1.00 |
| | | | | | **Sukharikha** | | | | | | | |
| 356 | | | 15.43 | 1.15 | 11.38 | 1.16 | 11.96 | 1.53 | 15.99 | 1.16 | BD | |
| 358 | | | 29.85 | 1.28 | 23.41 | 1.32 | 1.16 | 0.94 | 27.80 | 1.26 | BD | |
| 359 | | | 11.12 | 1.10 | 9.35 | 1.13 | 3.59 | 1.13 | 13.09 | 1.13 | BD | |
| 362 | | | 22.46 | 1.21 | 14.45 | 1.18 | 2.38 | 1.02 | 24.50 | 1.24 | BD | |
| 364 | | | 3.09 | 1.02 | 3.97 | 1.05 | 0.64 | 1.01 | 9.68 | 1.11 | BD | |
| 367 | | | 17.22 | 1.16 | 14.48 | 1.20 | 2.63 | 1.06 | 15.37 | 1.14 | BD | |
| 370 | | | 11.43 | 1.11 | 9.58 | 1.13 | 5.88 | 1.24 | 17.37 | 1.19 | BD | |
| 372 | | | 16.66 | 1.16 | 12.26 | 1.17 | 5.09 | 1.19 | 14.37 | 1.13 | BD | |
| 373 | | | 15.78 | 1.15 | 12.65 | 1.17 | 5.34 | 1.20 | 21.25 | 1.22 | BD | |
| 374 | | | 16.99 | 1.17 | 11.75 | 1.16 | 7.42 | 1.30 | 15.40 | 1.15 | BD | |
| 375 | | | 12.57 | 1.11 | 13.13 | 1.19 | 3.25 | 1.10 | 18.87 | 1.20 | BD | |
| 377 | | | 9.98 | 1.09 | 7.49 | 1.10 | 7.92 | 1.35 | 13.90 | 1.15 | BD | |
| 378 | | | 9.57 | 1.09 | 7.47 | 1.10 | 10.47 | 1.48 | 11.28 | 1.11 | BD | |
| 379 | | | 6.95 | 1.03 | 8.67 | 1.10 | BD | | 12.78 | 1.11 | BD | |
| 381 | | | 12.77 | 1.12 | 10.08 | 1.14 | 1.23 | 1.01 | 22.78 | 1.26 | BD | |
| 383 | | | 10.22 | 1.10 | 8.15 | 1.11 | 17.24 | 1.81 | 13.32 | 1.14 | BD | |
| 386 | | | 11.05 | 1.10 | 9.36 | 1.12 | 1.26 | 1.01 | 16.30 | 1.17 | BD | |

| Sample ID | Ba (wt%) | BaEF* | V (ppm) | VEF* | Cr (ppm) | CrEF* | Cu (ppm) | CuEF* | Zn (ppm) | ZnEF* | Mo (ppm) | MoEF* |
|---|---|---|---|---|---|---|---|---|---|---|---|---|
| 388 | | | 12.27 | 1.12 | 8.79 | 1.12 | 7.26 | 1.31 | 17.20 | 1.19 | BD | |
| 390 | | | 10.92 | 1.10 | 7.38 | 1.10 | 2.45 | 1.08 | 14.58 | 1.16 | BD | |
| 21/31 | | | 63.91 | 1.04 | 47.25 | 1.66 | 6.38 | 1.08 | 61.50 | 1.61 | BD | |
| 21/33 | | | 14.92 | 1.04 | 11.62 | 1.17 | 3.52 | 1.12 | 17.17 | 1.18 | BD | |
| 21/37 | | | | | | | | | | | | |
| 21/39 | | | 10.22 | 1.01 | 7.70 | 1.10 | 3.09 | 1.10 | 20.82 | 1.24 | BD | |
| 21/41 | | | 8.26 | 1.01 | 7.24 | 1.11 | 5.60 | 1.25 | 43.87 | 1.57 | 0.09 | 1.04 |
| 21/42 | | | 27.01 | 1.01 | 23.50 | 1.32 | 14.82 | 1.61 | 68.49 | 1.82 | 0.03 | 0.91 |
| 21/43 | | | 37.84 | 1.01 | 31.74 | 1.44 | 10.67 | 1.37 | 33.16 | 1.29 | BD | |
| 21/44 | | | 4.29 | 1.00 | 4.40 | 1.06 | 4.36 | 1.20 | 8.82 | 1.10 | BD | |
| 21/45 | | | | | | | | | | | | |
| 21/47 | | | 15.15 | 1.00 | 14.10 | 1.20 | 2.41 | 1.05 | 29.19 | 1.33 | BD | |
| 21/49 | | | | | | | | | | | | |
| 21/50 | | | 6.36 | 1.00 | 6.29 | 1.09 | 0.88 | 1.02 | 12.22 | 1.14 | BD | |

**Table S3: Trace element data and I/(Ca+Mg).**

| Sample ID | Cd (ppm) | CdEF* | Th (ppm) | ThEF* | U (ppm) | UEF* | Re (ppb) | ReEF* | I (ppm) | I/(Ca+Mg) (µmol/mol) |
|---|---|---|---|---|---|---|---|---|---|---|
| | | | | | Selinde | | | | | |
| 2 | 0.35 | 4.14 | 1.21 | 1.03 | 0.35 | 1.06 | | | | |
| 3 | | | | | | | 7.86 | 20.63 | 0.15 | 0.42 |
| 6 | | | | | 0.25 | 1.08 | 3.83 | 10.55 | 0.18 | 1.01 |
| 9 | | | | | 1.05 | 1.22 | 4.81 | 12.86 | 0.26 | 0.8 |
| 11 | 0.32 | 3.88 | 0.89 | 1.03 | 0.39 | 1.1 | | | | |
| 14 | 0.26 | 3.31 | 1.34 | 1.08 | 0.44 | 1.13 | | | | |
| 15 | 0.29 | 3.59 | 1.3 | 1.04 | 0.48 | 1.11 | | | | |
| 16 | 0.33 | 3.88 | 2.24 | 1.05 | 0.65 | 1.1 | | | | |
| 18 | 0.18 | 2.57 | 1.57 | 1.06 | 0.48 | 1.1 | | | | |
| 19 | 0.18 | 2.53 | 1.7 | 1.06 | 0.54 | 1.11 | | | 0.22 | 1.35 |
| 20 | 0.1 | 1.85 | 2.15 | 1.1 | 0.64 | 1.15 | | | | |
| 31 | 0.34 | 4.02 | 1.63 | 1.08 | 0.67 | 1.19 | | | | |
| 35 | 0.18 | 2.5 | 2.97 | 1.11 | 0.93 | 1.2 | 0.49 | 2.06 | 0.13 | 0.74 |
| 37 | 0.25 | 3.22 | 1.46 | 1.07 | 0.61 | 1.17 | | | | |
| 41 | 0.2 | 2.76 | 1.83 | 1.08 | 0.49 | 1.1 | | | 0.2 | 1.36 |
| 45 | 0.17 | 2.52 | 0.39 | 1.03 | 0.37 | 1.13 | | | | |
| 47 | | | | | 0.44 | 1.1 | 3.63 | 10 | 1.39 | |
| 49 | 0.52 | 5.69 | 1.13 | 1.04 | 0.48 | 1.12 | | | | |
| 53 | 0.23 | 2.97 | 1.65 | 1.04 | 0.53 | 1.09 | | | 0.25 | 1.61 |
| 57 | 0.19 | 2.61 | 1.92 | 1.05 | 0.61 | 1.11 | | | | |
| 58 | 0.27 | 3.36 | 1.59 | 1.05 | 0.55 | 1.12 | | | 0.23 | 1.56 |
| 62 | 0.2 | 2.72 | 1.85 | 1.04 | 0.64 | 1.12 | | | | |
| 65(2) | 0.08 | 1.51 | 3.78 | 1.11 | 1.06 | 1.17 | 0.36 | 1.67 | 0.38 | 1.81 |
| 67(2) | 0.2 | 2.73 | 1.1 | 1.03 | 0.32 | 1.05 | | | | |
| 69 | 0.19 | 2.62 | 1.66 | 1.03 | 0.54 | 1.09 | | | | |
| 71 | 0.16 | 2.34 | 1.5 | 1.03 | 0.53 | 1.1 | | | | |
| 72 | 0.17 | 2.49 | 1.41 | 1.04 | 0.46 | 1.09 | | | | |
| 74 | 0.13 | 2.08 | 1.88 | 1.04 | 0.59 | 1.1 | | | 0.46 | 2.46 |
| 77 | | | | | 0.32 | 1.03 | 3.98 | 10.86 | 0.38 | 2.12 |
| 82 | 0.18 | 2.55 | 0.98 | 1.03 | 0.41 | 1.1 | | | | |
| 84 | 0.21 | 2.78 | 2.22 | 1.06 | 0.82 | 1.17 | 0.43 | 1.94 | 0.29 | 1.83 |
| 86 | 0.19 | 2.53 | 2.64 | 1.07 | 0.97 | 1.21 | | | 0.27 | 1.36 |
| 89 | 0.23 | 2.95 | 1.85 | 1.05 | 0.65 | 1.13 | 0.38 | 1.83 | | |
| 90 | | | | | 0.57 | 1.12 | 8.02 | 20.97 | 0.49 | 2.5 |
| 92 | 0.17 | 2.54 | 0.67 | 1.02 | 0.42 | 1.12 | | | | |
| 95 | | | | | 0.86 | 1.19 | 4.99 | 13.34 | 0.39 | 1.44 |
| 98 | 0.23 | 3.01 | 0.69 | 1.02 | 0.44 | 1.12 | | | | |
| 101 | | | | | 0.79 | 1.18 | 5.39 | 14.37 | 0.32 | 1.54 |
| 103 | 0.21 | 2.84 | 0.95 | 1.01 | 0.67 | 1.18 | | | | |
| 105 | 0.3 | 3.67 | 0.99 | 1.01 | 0.57 | 1.14 | | | 0.23 | 1.52 |
| 109 | | | | | 0.58 | 1.12 | 4.25 | 11.52 | 0.31 | 1.59 |
| 111 | | | | | 0.49 | 1.12 | 3.23 | 9.01 | 0.3 | 1.59 |

| Sample ID | Cd (ppm) | CdEF* | Th (ppm) | ThEF* | U (ppm) | UEF* | Re (ppb) | ReEF* | I (ppm) | I/(Ca+Mg) (µmol/mol) |
|---|---|---|---|---|---|---|---|---|---|---|
| 114 | 0.17 | 2.53 | 0.82 | 1.02 | 0.44 | 1.11 | | | | |
| 115 | | | | | 0.56 | 1.13 | 3.21 | 8.95 | 0.31 | 1.62 |
| 116 | | | | | 0.62 | 1.14 | 4.11 | 11.19 | 0.28 | 1.29 |
| 117 | 0.16 | 2.33 | 1.41 | 1.04 | 0.68 | 1.16 | | | | |
| 120 | 0.2 | 2.75 | 1.2 | 1.03 | 0.55 | 1.13 | | | 0.28 | 1.76 |
| 122 | 0.17 | 2.43 | 1.05 | 1.02 | 0.51 | 1.12 | | | 0.15 | 0.87 |
| 124 | 0.13 | 2.09 | 1.19 | 1.02 | 0.84 | 1.23 | | | | |
| 125 | | | | | 0.6 | 1.15 | 5.1 | 13.68 | 0.21 | 0.96 |
| 127 | | | | | 0.41 | 1.08 | 5.28 | 14.12 | 0.28 | 1.48 |
| 128 | 0.19 | 2.69 | 0.76 | 1.02 | 0.48 | 1.13 | | | | |
| 129 | | | | | | | 3.66 | 10.12 | 0.25 | 1.37 |
| 131 | | | | | 0.53 | 1.11 | 2.86 | 8.05 | 0.39 | 1.98 |
| 132 | 0.08 | 1.68 | 0.65 | 1.02 | 0.34 | 1.09 | | | | |
| 134 | 0.12 | 2.03 | 0.8 | 1.02 | 0.36 | 1.08 | | | 0.2 | 1.31 |
| 136 | 0.19 | 2.64 | 1.12 | 1.04 | 0.53 | 1.14 | | | 0.21 | 1.25 |
| 137 | 0.17 | 2.5 | 0.68 | 1.03 | 0.41 | 1.12 | | | | |
| 140 | 0.11 | 1.97 | 0.61 | 1.03 | 0.43 | 1.13 | | | | |
| 142 | 0.08 | 1.72 | 0.24 | 1.01 | 0.21 | 1.07 | | | | |
| 144 | 0.11 | 1.96 | 0.57 | 1.02 | 0.27 | 1.07 | | | | |
| 145 | | | | | 0.49 | 1.16 | 3.65 | 10.11 | 0.31 | 1.69 |
| 146 | | | | | 0.46 | 1.12 | 3.57 | 9.88 | 0.3 | 1.65 |
| 147 | 0.1 | 1.82 | 0.87 | 1.02 | 0.95 | 1.3 | | | | |
| 149 | | | | | 0.39 | 1.11 | 2.53 | 7.3 | 0.26 | 1.41 |
| 150 | | | | | 0.53 | 1.17 | 4.35 | 11.85 | 0.23 | 1.28 |
| 152 | 0.11 | 2 | 0.18 | 1 | 0.1 | 1.03 | | | | |
| **Sukharikha** | | | | | | | | | | |
| 356 | | | | | 0.38 | 1.09 | 4.92 | 13.23 | 0.16 | 0.92 |
| 358 | | | | | 0.54 | 1.08 | 5.99 | 15.85 | 0.18 | 0.98 |
| 359 | | | | | 0.25 | 1.04 | 4.1 | 11.2 | 0.13 | 0.73 |
| 362 | | | | | 0.33 | 1.03 | 7.66 | 20.05 | 0.2 | 1.13 |
| 364 | | | | | BD | | 4.25 | 11.6 | 0.14 | 0.79 |
| 367 | | | | | 0.28 | 1.03 | 3.59 | 9.9 | 0.16 | 0.91 |
| 370 | | | | | 0.23 | 1.04 | 4.15 | 11.32 | 0.12 | 0.64 |
| 372 | | | | | 0.23 | 1.02 | 4.43 | 12 | 0.11 | 0.59 |
| 373 | | | | | 0.42 | 1.09 | 5.32 | 14.22 | 0.12 | 0.63 |
| 374 | | | | | 1.38 | 1.46 | 4.4 | 11.92 | 0.09 | 0.47 |
| 375 | | | | | 0.38 | 1.08 | 4.66 | 12.58 | 0.14 | 0.71 |
| 377 | | | | | 0.17 | 1.02 | 3.64 | 10.06 | 0.27 | 1.51 |
| 378 | | | | | 0.16 | 1.02 | 3.19 | 8.93 | 0.28 | 1.56 |
| 379 | | | | | 0.23 | 1.02 | 5.13 | 13.76 | 0.21 | 1.08 |
| 381 | | | | | 0.22 | 1.03 | 5.36 | 14.35 | 0.26 | 1.46 |
| 383 | | | | | 0.31 | 1.07 | 2.68 | 7.65 | 0.2 | 1.11 |
| 386 | | | | | 0.29 | 1.06 | 4.94 | 13.29 | 0.27 | 1.5 |
| 388 | | | | | 0.3 | 1.06 | 4.11 | 11.22 | 0.27 | 1.49 |

| Sample ID | Cd (ppm) | CdEF* | Th (ppm) | ThEF* | U (ppm) | UEF* | Re (ppb) | ReEF* | I (ppm) | I/(Ca+Mg) (µmol/mol) |
|---|---|---|---|---|---|---|---|---|---|---|
| 390 | | | | | 0.5 | 1.14 | 4.35 | 11.84 | 0.2 | 1.12 |
| **Ulakhan-Ald'yarkhay** | | | | | | | | | | |
| 21/31 | | | | | 3.12 | 1.94 | 9.37 | 24.18 | 0.12 | 0.51 |
| 21/33 | | | | | 3.06 | 2.1 | 6.46 | 17.11 | 0.07 | 0.42 |
| 21/37 | | | | | | | | | 0.07 | 0.45 |
| 21/39 | | | | | 0.74 | 1.23 | 5.41 | 14.47 | 0.06 | 0.38 |
| 21/41 | | | | | 1.06 | 1.37 | 9.28 | 24.18 | 0.06 | 0.36 |
| 21/42 | | | | | 0.61 | 1.11 | 6.71 | 17.66 | 0.08 | 0.29 |
| 21/43 | | | | | 0.84 | 1.15 | 6.07 | 16.02 | 0.08 | 0.31 |
| 21/44 | | | | | 0.3 | 1.09 | 7.49 | 19.71 | 0.06 | 0.37 |
| 21/45 | | | | | | | | | 0.09 | 0.5 |
| 21/47 | | | | | 0.26 | 1.03 | 5.23 | 14 | 0.23 | 1.39 |
| 21/49 | | | | | | | | | 0.15 | 1.06 |
| 21/50 | | | | | 0.18 | 1.04 | 6.12 | 16.28 | 0.28 | 1.73 |

**Table S4: Fe speciation data.**

| Sample ID | $Fe_{carb}$ (wt%) | $Fe_{ox}$ (wt%) | $Fe_{mag}$ (wt%) | $Fe_{py}$ (wt%) | $Fe_{HR}/Fe_T$ | $Fe_{py}/Fe_{HR}$ |
|---|---|---|---|---|---|---|
| | | | **Selinde** | | | |
| 2 | 0.20 | 0.15 | 0.01 | 0.00 | 0.64 | 0.00 |
| 9 | 0.19 | 0.71 | 0.08 | 0.00 | 0.51 | 0.00 |
| 15 | 0.18 | 2.50 | 0.05 | 0.00 | 1.00 | 0.00 |
| 16 | 0.14 | 0.61 | 0.02 | 0.00 | 0.54 | 0.00 |
| 18 | 0.09 | 0.39 | 0.02 | 0.00 | 0.58 | 0.00 |
| 19 | 0.09 | 0.53 | 0.02 | 0.00 | 0.56 | 0.00 |
| 20 | 0.09 | 0.66 | 0.03 | 0.00 | 0.74 | 0.00 |
| 31 | 0.27 | 0.47 | 0.02 | 0.00 | 0.70 | 0.00 |
| 35 | 0.12 | 0.53 | 0.02 | 0.00 | 0.40 | 0.00 |
| 37 | 0.19 | 0.54 | 0.02 | 0.00 | 0.84 | 0.00 |
| 41 | 0.10 | 0.54 | 0.02 | 0.00 | 0.72 | 0.00 |
| 47 | 0.02 | 0.39 | 0.03 | 0.01 | 0.49 | 0.01 |
| 49 | 0.09 | 0.29 | 0.04 | 0.00 | 0.66 | 0.00 |
| 53 | 0.18 | 0.66 | 0.09 | 0.00 | 0.78 | 0.00 |
| 57 | 0.26 | 0.60 | 0.07 | 0.00 | 0.69 | 0.00 |
| 58 | 0.16 | 0.50 | 0.08 | 0.00 | 0.63 | 0.00 |
| 62 | 0.25 | 0.57 | 0.08 | 0.00 | 0.64 | 0.00 |
| 65(2) | 0.52 | 1.38 | 0.13 | 0.00 | 0.75 | 0.00 |
| 67(2) | 0.13 | 0.50 | 0.05 | 0.00 | 0.87 | 0.00 |
| 69 | 0.26 | 0.70 | 0.08 | 0.00 | 0.78 | 0.00 |
| 71 | 0.21 | 0.55 | 0.09 | 0.00 | 0.70 | 0.00 |
| 72 | 0.19 | 0.42 | 0.06 | 0.00 | 0.63 | 0.00 |
| 74 | 0.35 | 0.94 | 0.15 | 0.00 | 1.00 | 0.00 |
| 77 | 0.02 | 0.34 | 0.03 | 0.00 | 0.39 | 0.01 |
| 82 | 0.16 | 0.38 | 0.03 | 0.00 | 0.80 | 0.00 |
| 84 | 0.25 | 0.65 | 0.07 | 0.00 | 0.56 | 0.00 |
| 86 | 0.29 | 0.87 | 0.11 | 0.00 | 0.70 | 0.00 |

| Sample ID | $Fe_{carb}$ (wt%) | $Fe_{ox}$ (wt%) | $Fe_{mag}$ (wt%) | $Fe_{py}$ (wt%) | $Fe_{HR}/Fe_T$ | $Fe_{py}/Fe_{HR}$ |
|---|---|---|---|---|---|---|
| 89 | 0.23 | 0.36 | 0.09 | 0.00 | 0.64 | 0.00 |
| 90 | 0.02 | 0.24 | 0.03 | 0.00 | 0.31 | 0.00 |
| 95 | 0.03 | 0.48 | 0.05 | 0.00 | 0.40 | 0.00 |
| 101 | 0.03 | 0.46 | 0.05 | 0.01 | 0.37 | 0.01 |
| 103 | 0.18 | 0.53 | 0.06 | 0.00 | 0.83 | 0.00 |
| 105 | 0.14 | 0.49 | 0.06 | 0.00 | 0.69 | 0.00 |
| 109 | 0.03 | 0.39 | 0.06 | 0.00 | 0.40 | 0.00 |
| 115 | 0.02 | 0.37 | 0.04 | 0.00 | 0.43 | 0.01 |
| 116 | 0.04 | 0.41 | 0.05 | 0.00 | 0.44 | 0.00 |
| 117 | 0.15 | 0.49 | 0.05 | 0.00 | 0.59 | 0.00 |
| 120 | 0.18 | 0.46 | 0.07 | 0.00 | 0.73 | 0.00 |
| 122 | 0.23 | 0.37 | 0.05 | 0.00 | 0.74 | 0.00 |
| 124 | 0.20 | 0.10 | 0.03 | 0.00 | 0.55 | 0.00 |
| 125 | 0.10 | 0.34 | 0.05 | 0.00 | 0.49 | 0.00 |
| 127 | 0.04 | 0.14 | 0.02 | 0.00 | 0.32 | 0.00 |
| 131 | 0.01 | 0.24 | 0.02 | 0.01 | 0.28 | 0.02 |
| 134 | 0.07 | 0.27 | 0.02 | 0.00 | 0.56 | 0.01 |
| 136 | 0.11 | 0.39 | 0.03 | 0.00 | 0.77 | 0.00 |
| 146 | 0.05 | 0.16 | 0.02 | 0.00 | 0.42 | 0.00 |
| 147 | 0.16 | 0.32 | 0.04 | 0.00 | 0.75 | 0.00 |
| | | | **Sukharikha** | | | |
| 356 | 0.08 | 0.08 | 0.01 | 0.00 | 0.33 | 0.01 |
| 358 | 0.07 | 0.63 | 0.06 | 0.00 | 0.43 | 0.00 |
| 362 | 0.08 | 0.16 | 0.01 | 0.00 | 0.33 | 0.00 |
| 367 | 0.07 | 0.09 | 0.02 | 0.00 | 0.25 | 0.00 |
| 372 | 0.08 | 0.09 | 0.01 | 0.00 | 0.34 | 0.01 |
| 373 | 0.16 | 0.02 | 0.01 | 0.00 | 0.29 | 0.00 |
| 374 | 0.21 | 0.02 | 0.01 | 0.08 | 0.54 | 0.26 |
| 375 | 0.23 | 0.06 | 0.01 | 0.00 | 0.58 | 0.01 |

| Sample ID | $Fe_{carb}$ (wt%) | $Fe_{ox}$ (wt%) | $Fe_{mag}$ (wt%) | $Fe_{py}$ (wt%) | $Fe_{HR}/Fe_T$ | $Fe_{py}/Fe_{HR}$ |
|---|---|---|---|---|---|---|
| 379 | 0.24 | 0.04 | 0.01 | 0.00 | 0.53 | 0.01 |
| 388 | 0.06 | 0.10 | 0.01 | 0.00 | 0.33 | 0.00 |
| **Ulakhan-Ald'yarkhay** | | | | | | |
| 21/31 | 1.18 | 0.10 | 0.10 | 0.00 | 0.47 | 0.00 |
| 21/33 | 0.30 | 0.05 | 0.04 | 0.01 | 0.54 | 0.01 |
| 21/39 | 0.29 | 0.06 | 0.02 | 0.01 | 0.68 | 0.02 |
| 21/42 | 1.54 | 0.08 | 1.54 | 0.26 | 1.28 | 0.08 |
| 21/43 | 1.50 | 0.07 | 0.02 | 0.29 | 0.70 | 0.15 |
| 21/47 | 0.08 | 0.41 | 0.07 | 0.00 | 0.42 | 0.00 |

**Table S5: Fossil occurrences throughout sampled time interval.**

| Species | Affinity | Skeleton | Reference |
|---|---|---|---|
| **Selinde** | | | |
| *Tiksitheca licis* | anabaritid | aragonite | 72 |
| *Cambrotubulus decurvatus* | anabaritid | aragonite | 73 |
| *Selindeochrea tricarinata* | anabaritid | aragonite | 74 |
| *Anabarites trisulcatus* | anabaritid | aragonite | 73 |
| *Selindeochrea ternaria* | anabaritid | aragonite | 74 |
| *Anabarites kelleri* | anabaritid | aragonite | 72 |
| *Anabarites hexasulcatus* | anabaritid | aragonite | 74 |
| *Selindeochrea tripartita* | anabaritid | aragonite | 74 |
| *Anabarites tristichus* | anabaritid | aragonite | 72 |
| *Mariochrea sinuosa* | anabaritid | aragonite | 72 |
| *Selindeochrea tecta* | anabaritid | aragonite | 73 |
| *Gastreochrea viva* | anabaritid | aragonite | 72 |
| *archaeocyath fragments* | archaeocyath | Mg-calcite | 113 |
| *Nochoroicyathus mirabilis* | archaeocyath | Mg-calcite | 114 |
| *Cambrocyathellus tschuranicus* | archaeocyath | Mg-calcite | 114 |
| *Nochoroicyathus "arteintervallum"* | archaeocyath | Mg-calcite | 114 |
| *Neoloculicyathus sibiricus* | archaeocyath | Mg-calcite | 114 |
| *Dictyosycon gravis* | archaeocyath | Mg-calcite | 114 |
| *Chancelloriida gen. & sp. indet.* | chancelloriid | aragonite | 115 |
| *Coleolus trigonus* | coleolid | aragonite | 116 |
| *Halkieria sacciformis* | halkieriid | aragonite | 73 |
| *Siphogonuchites triangualaris* | halkieriid | aragonite | 117 |
| *Halkieria proboscidea* | halkieriid | aragonite | 73 |
| *Hyolithellus vladimirovae* | hyolithelminth | phosphate | 73 |
| *Hyolithellus tenuis* | hyolithelminth | phosphate | 73 |
| *Hyolithellus grandis* | hyolithelminth | phosphate | 73 |
| *Torellella lentiformis* | hyolithelminth | phosphate | 116 |
| *Torellella biconvexa* | hyolithelminth | phosphate | 30 |
| *Notabilitus simplex* | hyolithomorph | calcite? | 73 |
| *Oblisicornus dupleconcavus* | hyolithomorph | calcite? | 73 |
| *Notabilitus orientalis* | hyolithomorph | calcite? | 73 |
| *Burithes distortus* | hyolithomorph | calcite? | 73 |
| *Crestjahitus figuratus* | hyolithomorph | calcite? | 73 |
| *Dorsojugatus sp.* | hyolithomorph | calcite? | 30 |
| *Doliutus sp.* | hyolithomorph | calcite? | 30 |
| *Mobergella radiolata* | mobergellid | phosphate | 118 |
| *Igorella ungulata* | mollusc | aragonite | 119 |
| *Oelandiella korobkovi* | mollusc | aragonite | 117 |

| | | | |
|---|---|---|---|
| *Bemella jacutica* | mollusc | aragonite | 116 |
| *Hamusella rustica* | mollusc | aragonite | 73 |
| *Aldanella crassa* | mollusc | aragonite | 120 |
| *Aldanella rozanovi* | mollusc | aragonite | 121 |
| *Aldanella attleborensis* | mollusc | aragonite | 120 |
| *Anabarella plana* | mollusc | aragonite | 122 |
| *Purella antiqua* | mollusc | aragonite | 123 |
| *Purella cristata* | mollusc | aragonite | 121 |
| *Mellopegma indecora* | mollusc | aragonite | 124 |
| *Nomgoliella sinistrovolubilis* | mollusc | aragonite | 75 |
| *Securiconus costulatus* | mollusc | aragonite | 116 |
| *Watsonella crosbyi* | mollusc | aragonite | 117 |
| *Barskovia hemisymmetrica* | mollusc | aragonite | 121 |
| *Aldanella utchurica* | mollusc | aragonite | 120 |
| *Ceratoconus striatus* | mollusc | aragonite | 116 |
| *Nomgoliella rotunda* | mollusc | aragonite | 112 |
| *Watsonella crosbyi* | mollusc | aragonite | 117 |
| *Bemella parula* | mollusc | aragonite | 115 |
| *Pseudoscenella gracilis* | mollusc | aragonite | 119 |
| *Aldanella sibirica* | mollusc | aragonite | 120 |
| *Turcutheca crassecochlia* | orthothecimorph | aragonite | 116 |
| *Turcutheca rugata* | orthothecimorph | aragonite | 116 |
| *Ladatheca annae* | orthothecimorph | aragonite | 73 |
| *Ladatheca dorsocava* | orthothecimorph | aragonite | 72 |
| *Kotuitheca curta* | orthothecimorph | aragonite | 73 |
| *Turcutheca cotujensis* | orthothecimorph | aragonite | 124 |
| *Ovalitheca mongolica* | orthothecimorph | aragonite | 124 |
| *Spinulitheca rotunda* | orthothecimorph | aragonite | 73 |
| *Kugdatheca voluta* | orthothecimorph | aragonite | 124 |
| *Spinulitheca billingsi* | orthothecimorph | aragonite | 73 |
| *Exilitheca multa* | orthothecimorph | aragonite | 73 |
| *Conotheca mammilata* | orthothecimorph | aragonite | 116 |
| *Loculitheca anulata* | orthothecimorph | aragonite | 73 |
| *Nikatheca digna* | orthothecimorph | aragonite | 73 |
| *Allatheca cana* | orthothecimorph | aragonite | 73 |
| *Allatheca concinna* | orthothecimorph | aragonite | 116 |
| *Spinulitheca aff. limbulirata* | orthothecimorph | aragonite | 30 |
| *Tchuranitheca curvata* | orthothecimorph | aragonite | 73 |
| *Allatheca corrugata* | orthothecimorph | aragonite | 73 |
| *Tchuranitheca sinuata* | orthothecimorph | aragonite | 73 |
| *Eonovitatus superbus* | orthothecimorph | aragonite | 73 |
| *Obliquatheca aldanica* | orthothecimorph | aragonite | 73 |
| *Gracilitheca ternata* | orthothecimorph | aragonite | 73 |
| *Pseudoorthotheca sp.* | problematic | phosphate | 124 |

| | | | |
|---|---|---|---|
| *Fomitchella acinaciformis* | protoconodont | phosphate | 124 |
| *Fomitchella infundibuliformis* | protoconodont | phosphate | 116 |
| *Lapworthella sp.* | tommotiid | phosphate | 30 |
| *Tommotia sp.* | tommotiid | phosphate | 30 |
| *Camenella garbowskae* | tommotiid | phosphate | 116 |
| *Tommotia zonata* | tommotiid | phosphate | 30 |
| *Profallotaspis privica* | trilobite | calcite | 30 |
| *Profallotaspis sp.* | trilobite | calcite | 30 |
| *Profallotaspis jacutensis* | trilobite | calcite | 30 |
| *Repinaella explicata* | trilobite | calcite | 125 |
| *Repinaella sibirica* | trilobite | calcite | 125 |
| *Bigotinella malycanica* | trilobite | calcite | 126 |
| *Selindella sp.* | trilobite | calcite | 30 |
| *Pelmanaspis jurii* | trilobite | calcite | 127 |
| *Elganellus pensus* | trilobite | calcite | 30 |
| *Elganellus elegans* | trilobite | calcite | 127 |
| *Selindella gigantea* | trilobite | calcite | 128 |
| *Triangullina parvula* | trilobite | calcite | 30 |
| *Nevadella aff. effusa* | trilobite | calcite | 30 |
| *Bemella* | mollusc | aragonite? | 119 |
| *Oelandiella korobkovi* | mollusc | aragonite? | 119 |
| *Igorella* | mollusc | aragonite? | 119 |
| *Obtusoconus rotriptuetus* | mollusc | aragonite? | 119 |
| **Sukharikha** | | | |
| *Halkieria sp.* | halkieriid | aragonite | 165 |
| *Trapezovitus sp.* | hyolithomorph | calcite? | 129 |
| *Tetratheca clinisepta* | orthothecimorph | aragonite | 129 |
| **Ulakhan-Ald'Yarkhay** | | | |
| *Archaeolynthus polaris* | archaeocyath | Mg-calcite | 19 |
| *Nochoroicyathus tkatchenkoi* | archaeocyath | Mg-calcite | 19 |
| *Nochoroicyathus subturbidus* | archaeocyath | Mg-calcite | 19 |
| *Rotundocyathus? grandis* | archaeocyath | Mg-calcite | 19 |
| *Rotundocyathus orbus* | archaeocyath | Mg-calcite | 19 |
| *Rotundocyathus tetracyclicus* | archaeocyath | Mg-calcite | 19 |
| *Rotundocyathus apertus* | archaeocyath | Mg-calcite | 19 |
| *Taylorcyathus eximius* | archaeocyath | Mg-calcite | 164 |
| *Lenocyathus lenaicus* | archaeocyath | Mg-calcite | 164 |
| *Retecoscinus proximus* | archaeocyath | Mg-calcite | 164 |
| *Korshunovicyathus melnikovi* | archaeocyath | Mg-calcite | 19 |
| *Halkieria sacciformis* | halkieriid | aragonite | 164 |
| *Hyolithellus vladimirovae* | hyolithelminth | phosphate | 164 |
| *Torellella sp.* | hyolithelminth | phosphate | 164 |
| *Crestjahitus figuratus* | hyolithomorph | calcite? | 164 |
| *Microcornus cf. eximius* | hyolithomorph | calcite? | 75 |

| | | | |
|---|---|---|---|
| *Burithes triangularis* | hyolithomorph | calcite? | 164 |
| *Atdabanites infractus* | hyolithomorph | calcite? | 164 |
| *Doliutus inflatus* | hyolithomorph | calcite? | 164 |
| *Mobergella radiolata* | mobergellid | phosphate | 118 |
| *Fordilla sibirica* | mollusc | phosphate | 130 |
| *Turcutheca crassecochlia* | orthothecimorph | aragonite | 164 |
| *Conotheca mammilata* | orthothecimorph | aragonite | 164 |
| *Majatheca tumefacta* | orthothecimorph | aragonite | 164 |
| *Obliquatheca bicostata* | orthothecimorph | aragonite | 131 |
| *Conotheca circumflexa* | orthothecimorph | aragonite | 164 |
| *C. cf. longiconica* | orthothecimorph | aragonite | 75 |
| *Tetratheca clinisepta* | orthothecimorph | aragonite | 164 |
| *Minitheca multa* | orthothecimorph | aragonite | 164 |
| *Lapworthella bella* | tommotiid | phosphate | 164 |
| *Lapworthella corniformis* | tommotiid | phosphate | 164 |
| *Lapworthella lata* | tommotiid | phosphate | 164 |
| *Camenella complicata* | tommotiid | phosphate | 164 |
| *Lapworthella lucida* | tommotiid | phosphate | 164 |
| *Profallotaspis tyusserica* | trilobite | calcite | 32 |
| *Repinaella explicata* | trilobite | calcite | 32 |

**Table S6: Fossil-bearing sites from the Tommotian 1, 3, and Atdabanian 1 ($\delta^{13}$C peak 6p/7p, III, and IV), with references, formation names.** Sections constrained by a $\delta^{13}$C record in **bold.** Possibly of a later age is highlighted in grey.

| Sections | References | Formation |
|---|---|---|
| ***Lower Tommotian sections ($\delta^{13}$C peak 6p/7p)*** | | |
| **Sukharikha River** | 109 | Sukharikha |
| GR-13 borehole (Gremyaka area)/1 | 34 | Polba |
| Megelyakh-1151 borehole/55 | 34 | Bilir |
| Middle Botuoba-10 borehole/58 | 34 | Bilir |
| Middle Markha-2250 borehole/60 | 34 | Bilir |
| Sokhsogolokh-706 borehole/79 | 34 | Bilir |
| Bysytykh-1201 borehole/89 | 34 | Bilir |
| Ledyanskaya-358 borehole/91 | 34 | Kheta |
| 3г, Г-5 boreholes (Anabar area)/93 | 34 | Emyaksin |
| **Bol'shaya Kuonamka River** | 75, 106 | Emyaksin |
| **Kotuykan River** | 106, 107 | Medvezh'ya |
| **Kotuy River/Kugda section** | 106, 109 | Medvezh'ya |
| **Kotuy River/Ary-Mas-Yuryakh section** | 106, 109 | Medvezh'ya |
| Kotuy River/Odikhincha section | 109 | Medvezh'ya |
| Kotuy River/Eriechka section | 109 | Medvezh'ya |
| Kotuy River/Fomich section | 109 | Medvezh'ya |
| Nekekit River/95 | 107 | Erkeket |
| Kenyada River/96 | 34, 73 | Emyaksin |
| **Khorbusuonka River/98** | 105 | Mattaia |
| borehole 203/102 | 34 | Emyaksin |
| **Eyik-343-O borehole/103** | 108 | Yuryakh |
| Gynym River | 104 | Pestrotsvet |
| Gonam River | 132 | Pestrotsvet |
| Berdyakit River | 104 | Pestrotsvet |
| Chakdala River | 104 | Pestrotsvet |
| **Selinde River** | 30 etc. | Pestrotsvet |
| Dzhanda River | 103, 133 | Pestrotsvet |

***Tommotian 3 sections***
***($\delta^{13}C$ peak III)***

| Sections | References | Formation |
|---|---|---|
| **Sukharikha River /20** | 109 etc. | Krasny Porog |
| ИС-133 borehole /14 | 34 | Krasny Porog |
| Gorbiyachin River? | 134 | Krasny Porog |
| Brus River /19? | 134 | Krasny Porog |
| 2-Б borehole | 135 | Usol'e |
| borehole 54 (Troitsk anticline)/38 | 135 | Usol'e |
| Nepa 7 borehole /46 | 34, 136 | Usol'e |
| Bratsk 2 borehole | 136 | Usol'e |
| Markovo 17 borehole | 135 | Usol'e |
| Markovo 27 borehole | 135 | Usol'e |
| Krivolutskaya 3 borehole | 135 | Usol'e |
| Osa River borehole | 137 | Usol'e |
| Markovo 23-p borehole /47 | 34 | Usol'e |
| Yarakta 12 borehole /48 | 136 | Usol'e |
| Megelyakh-1151 /55 | 34 | Sygdakh |
| Ambardakh-Maymecha rivers | 138 | Medvezh'ya |
| Onkhoydokh-252-0 borehole /74? | 34 | Sygdakh |
| Onkuchakh-2861 borehole /76 | 34 | Emyaksin |
| Markha 1 borehole /87? | 139 | Emyaksin |
| Orto-Siligir 2621 borehole /90? | 34 | Emyaksin |
| 3г, Г-5 (Anabar area)/93 | 34 | Emyaksin |
| **Bol'shaya & Malaya Kuonamka rivers** | 73, 107, 140 | Emyaksin |
| Kotuy River/Odikhincha section | 109 | Medvezh'ya |
| Rassokha River | 141 | Emyaksin |
| Kotuy River/Fomich section | 109 | Medvezh'ya |
| Kyungkyuy-Yuryakh River | 141 | Medvezh'ya |
| Khastyr River | 141 | Medvezh'ya |
| Medvezh'ya & Daldyn rivers | 142, 143 | Medvezh'ya |
| Kenyada River/96 | 34, 73 | Emyaksin |
| **Khorbusuonka River/98** | 34, 75 | Nouyo |
| borehole 203 /102 | 34 | Emyaksin |
| **Eyik-343-O borehole /103** | 34 | Emyaksin |
| Chuchukan-1п borehole /104 | 34 | Pestrotsvet |
| **middle Lena River** | 109 etc. | Pestrotsvet |
| Nizhniy Kuranakh-27 borehole | 144 | Pestrotsvet |
| Gorely-16 borehole | 144 | Pestrotsvet |
| Kurum-Kyunkyu-26 borehole | 144 | Pestrotsvet |
| Ulu-3 borehole | 144 | Pestrotsvet |
| **middle Aldan River** | 109 etc. | Pestrotsvet |

| | | |
|---|---|---|
| Gynym River | 104, 145 | Pestrotsvet |
| **Sections** | **References** | **Formation** |
| Gonam River? | 132 etc. | Pestrotsvet |
| Berdyakit River? | 104 | Pestrotsvet |
| Chakdala River | 104 | Pestrotsvet |
| **Selinde River** | 30 etc. | Pestrotsvet |
| Dzhanda River | 103, 133 | Pestrotsvet |
| ***Atdabanian 1 sections*** ***($\delta^{13}$C peak IV)*** | | |
| **Sukharikha River /20** | 109 etc. | Krasny Porog |
| Lower Tungusska 6 borehole /22 | 34 | Abakun |
| Ust'-Deltula 214 borehole /29 | 34 | Abakun |
| borehole 54 (Troitsk anticline)/38 | 34 | Bel'sk |
| lower Angara River boreholes 2ц, 5л, 92 | 146 | Klimovo |
| Belaya & Urik rivers | 146 | Bel'sk |
| **Irkut River** | 146, 147 | Bel'sk |
| Nepa 1 borehole /46 | 34 | Bel'sk |
| Markovo 1 borehole /47a | 34 | Bel'sk |
| Vitim River mouth | 148 | El'gyan |
| Middle Ygyatty 2630 borehole /60 | 34 | El'gyan |
| Kederge 432 borehole /65 | 34 | El'gyan |
| Upper Cherendey 2170 & Kurdara 255019 boreholes /63, 69 | 34 | El'gyan |
| Ambardakh-Maymecha rivers | 138 | Kugda-Yuryakh |
| Nakyn 2950 borehole /75 | 34 | Sygdakh |
| Sokhsogolokh-706 borehole /79 | 34 | Emyaksin |
| Markha 1 borehole /87 | 139 | Emyaksin |
| **Bol'shaya & Malaya Kuonamka rivers** | 73, 107, 140 | Emyaksin |
| Kotuy River/Chomp-Yuryakh Creek | 149 | Kyndyn |
| Kotuy River/D'yama-Yuryakh Creek | 150 | Kyndyn |
| Kotuy River/Fomich section | 109, 151 | Parfen-Yuryakh |
| Kyungkyuy-Yuryakh River | 141 | Parfen-Yuryakh |
| Eriechka River | 141 | Parfen-Yuryakh |
| Medvezh'ya & Daldyn rivers | 142, 143 | Medvezh'ya |
| Nekekit & Olenek rivers | 107, 152, 153 | Erkeket |
| Kenyada River/96 | 34, 107, 152 | Emyaksin |

| | | |
|---|---|---|
| Khorbusuonka River/98 | 154, 155 | Erkeket |
| **Ulakhan-Ald'yarkhay Brook/99** | 75, 164 | Tyuser |
| Tyuser Brook | 164 | Tyuser |
| Chekurovka Village/100 | 164 | Tyuser |
| borehole 203 /102 | 34 | Emyaksin |
| Eyik-343-O borehole /103 | 34 | Emyaksin |
| Malykay 405 borehole /107 | 34 | El'gyan |
| **middle Lena River** | 109 etc. | Pestrotsvet |
| middle Aldan River | 109 etc. | Pestrotsvet |
| **Selinde River** | 30 etc. | Pestrotsvet |
| Dzhanda River | 133 | Pestrotsvet |

**Table S7: Trilobite temporal and spatial distribution.** Colour coding refers to study regions outlined in Figs. 1 and 2.

| Species | | Atdabanian 1 (ca. 521-519.5 Ma) | | | Stratigraphy | Systematics |
|---|---|---|---|---|---|---|
| | | ***Profallotaspis jakutensis* zone** | ***Repinella* zone** | ***Delgadella anabara* zone** | | |
| *Profallotaspis jakutensis* | Fallotaspidoidea | | | | 127 | 64 |
| *P. privica* | Fallotaspidoidea | | | | 127 | 64 |
| *P. tyusserica* | Fallotaspidoidea | | | | 32 | 64 |
| *Repinaella explicata* | Fallotaspidoidea | | | | 32 | 64 |
| *R. sibirica* | Fallotaspidoidea | | | | 127 | 64 |
| *Nevadella effusa* | Fallotaspidoidea | | | | 34 | 64 |
| *Archaeaspis hupei* | Fallotaspidoidea | | | | 156 | 64 |
| *Paranevadella subgroenlandica* | Fallotaspidoidea | | | | 32 | 64 |
| *Lenallina lata* | Fallotaspidoidea | | | | 127 | 64 |
| *Pelmanaspis jurii* | Fallotaspidoidea | | | | 127 | 64 |
| *"Paedemias" cf. groenlandicus* | Fallotaspidoidea | | | | 29 | 29 |
| *Selindella gigantea* | Fallotaspidoidea | | | | 127 | 64 |
| *Delgadella anabara* | Hebediscidae | | | | 157 | 64 |
| *D. pervulgata* | Hebediscidae | | | | 32 | 64 |
| *D. sakhaica* | Hebediscidae | | | | 32 | 64 |
| *Pauliceps granosa* | Redlichioidea | | | | 29 | 29 |
| *Elganellus probus* | Redlichioidea | | | | 157 | 64 |
| *E. pensus* | Redlichioidea | | | | 157 | 64 |
| *E. acceptus* | Redlichioidea | | | | 34 | 64 |
| *E. elegans* | Redlichioidea | | | | 34 | 64 |
| *"Comluella"? atdabanensis* | Redlichioidea | | | | 29 | 29 |
| *Pseudoresserpos oculatus* | Redlichioidea | | | | 156 | 64 |
| *Bigotina angulata* | Redlichioidea | | | | 158 | 126 |
| *B. copiosa* | Redlichioidea | | | | 159 | 126 |
| *Bigotinella rara* | Redlichioidea | | | | 160 | 160 |
| *B. malykanica* | Redlichioidea | | | | 34 | 126 |
| *Suvorovaella? patria* | Redlichioidea | | | | 29 | 126 |
| *Suvorovaella priva* | Redlichioidea | | | | 159 | 126 |
| *Tolbinella varlamovi* | Redlichioidea | | | | 157 | 157 |

| | | | | | | |
|---|---|---|---|---|---|---|
| *Malykania gribovae* | Corynexochoidea | | | | 29 | 157 |
| *M. nochtujensis* | Corynexochoidea | | | | 158 | 157 |
| *M. grandis* | Corynexochoidea | | | | 157 | 157 |
| *M. vaganovae* | Corynexochoidea | | | | 157 | 157 |
| *Compsocephalus generosus* | Corynexochoidea | | | | 156 | 156 |
| *Tetragonocephalus gratus* | Ellipsocephaloidea? | | | | 29 | 29 |
| *Triangullina parvula* | Ellipsocephaloidea? | | | | 29 | 64 |

## REFERENCES AND NOTES

1. D. H. Erwin, M. Laflamme, S. M. Tweedt, E. A. Sperling, D. Pisani, K. J. Peterson, The Cambrian conundrum: Early divergence and later ecological success in the early history of animals. *Science* **334**, 1091–1097 (2011).

2. J. R. Nursall, Oxygen as a prerequisite to the origin of the Metazoa. *Nature* **183**, 1170–1172 (1959).

3. E. A. Sperling, T. H. Boag, M. I. Duncan, C. R. Endriga, J. A. Marquez, D. B. Mills, P. M. Monarrez, J. A. Sclafani, R. G. Stockey, J. L. Payne, Breathless through time: Oxygen and animals across Earth's history. *Biol. Bull.* **243**, 184–206 (2022).

4. R. G. Stockey, D. B. Cole, U. C. Farrell, H. Agić, T. H. Boag, J. J. Brocks, D. E. Canfield, M. Cheng, P. W. Crockford, H. Cui, T. W. Dahl, L. Del Mouro, K. Dewing, S. Q. Dornbos, J. F. Emmings, R. R. Gaines, T. M. Gibson, B. C. Gill, G. J. Gilleaudeau, K. Goldberg, R. Guilbaud, G. Halverson, E. U. Hammarlund, K. Hantsoo, M. A. Henderson, C. M. Henderson, M. S. W. Hodgskiss, A. J. M. Jarrett, D. T. Johnston, P. Kabanov, J. Kimmig, A. H. Knoll, M. Kunzmann, M. A. LeRoy, C. Li, D. K. Loydell, F. A. Macdonald, J. M. Magnall, N. T. Mills, L. M. Och, B. O'Connell, A. Pagès, S. E. Peters, S. M. Porter, S. W. Poulton, S. R. Ritzer, A. D. Rooney, S. Schoepfer, E. F. Smith, J. V. Strauss, G. J. Uhlein, T. White, R. A. Wood, C. R. Woltz, I. Yurchenko, N. J. Planavsky, E. A. Sperling, Sustained increases in atmospheric oxygen and marine productivity in the Neoproterozoic and Palaeozoic eras. *Nat. Geosci.* **17**, 667–674 (2024).

5. B. J. W. Mills, A. J. Krause, I. Jarvis, B. D. Cramer, Evolution of atmospheric O2 through the Phanerozoic, revisited. *Annu. Rev. Earth Planet. Sci.* **51**, 253–276 (2023).

6. E. U. Hammarlund, R. R. Gaines, M. G. Prokopenko, C. Qi, X.-G. Hou, D. E. Canfield, Early Cambrian oxygen minimum zone-like conditions at Chengjiang. *Earth Planet. Sci. Lett.* **475**, 160–168 (2017).

7. R. Guilbaud, B. J. Slater, S. W. Poulton, T. H. P. Harvey, J. J. Brocks, B. J. Nettersheim, N. J. Butterfield, Oxygen minimum zones in the early Cambrian ocean. *Geochem. Perspect. Lett.* **6**, 33–38 (2018).

8. S. K. Sahoo, N. J. Planavsky, G. Jiang, B. Kendall, J. D. Owens, X. Wang, X. Shi, A. D. Anbar, T. W. Lyons, Oceanic oxygenation events in the anoxic Ediacaran ocean. *Geobiology* **14**, 457–468 (2016).

9. G.-Y. Wei, N. J. Planavsky, L. G. Tarhan, X. Chen, W. Wei, D. Li, H.-F. Ling, Marine redox fluctuation as a potential trigger for the Cambrian explosion. *Geology* **46**, 587–590 (2018).

10. G.-Y. Wei, N. J. Planavsky, L. G. Tarhan, T. He, D. Wang, G. A. Shields, W. Wei, H.-F. Ling, Highly dynamic marine redox state through the Cambrian explosion highlighted by authigenic δ238U records. *Earth Planet. Sci. Lett.* **544**, 116361 (2020).

11. T. W. Dahl, J. N. Connelly, D. Li, A. Kouchinsky, B. C. Gill, S. Porter, A. C. Maloof, M. Bizzarro, Atmosphere–ocean oxygen and productivity dynamics during early animal radiations. *Proc. Natl. Acad. Sci. U.S.A.* **116**, 19352–19361 (2019).

12. F. T. Bowyer, R. A. Wood, M. Yilales, Sea level controls on Ediacaran-Cambrian animal radiations. *Sci. Adv.* **10**, eado6462 (2024).

13. T. He, M. Zhu, B. J. W. Mills, P. M. Wynn, A. Y. Zhuravlev, R. Tostevin, P. A. E. P. von Strandmann, A. Yang, S. W. Poulton, G. A. Shields, Possible links between extreme oxygen perturbations and the Cambrian radiation of animals. *Nat. Geosci.* **12**, 468–474 (2019).

14. A. Y. Zhuravlev, E. G. Mitchell, F. Bowyer, R. Wood, A. M. Penny, Increases in reef size, habitat and metacommunity complexity associated with Cambrian radiation oxygenation pulses. *Nat. Commun.* **13**, 7523 (2022).

15. E. A. Sperling, C. A. Frieder, A. V. Raman, P. R. Girguis, L. A. Levin, A. H. Knoll, Oxygen, ecology, and the Cambrian radiation of animals. *Proc. Natl. Acad. Sci. U.S.A.* **110**, 13446–13451 (2013).

16. A. T. Cribb, C. G. Kenchington, B. Koester, B. M. Gibson, T. H. Boag, R. A. Racicot, H. Mocke, M. Laflamme, S. A. F. Darroch, Increase in metazoan ecosystem engineering prior to the Ediacaran-Cambrian boundary in the Nama Group Namibia. *R. Soc. Open Sci.* **6**, 190548 (2019).

17. R. Wood, A. Y. Zhuravlev, Escalation and ecological selectively of mineralogy in the Cambrian radiation of skeletons. *Earth Sci. Rev.* **115**, 249–261 (2012).

18. R. Wood, D. H. Erwin, Innovation not recovery: Dynamic redox promotes metazoan radiations. *Biol. Rev.* **93**, 863–873 (2018).

19. A. Y. Zhuravlev, R. A. Wood, F. T. Bowyer, Cambrian radiation speciation events driven by sea level and redoxcline changes on the Siberian craton. *Sci. Adv.* **9**, eadh2558 (2023).

20. A. Y. Zhuravlev, R. Wood, Dynamic and synchronous changes in metazoan body size during the Cambrian explosion. *Sci. Rep.* **10**, 6784 (2020).

21. T. W. Dahl, M.-L. Siggaard-Andersen, N. H. Schovsbo, D. O. Persson, S. Husted, I. W. Hougård, A. J. Dickson, K. Kjær, A. T. Nielsen, Brief oxygenation events in locally anoxic oceans during the Cambrian solves the animal breathing paradox. *Sci. Rep.* **9**, 11669 (2019).

22. R. A. Wood, S. W. Poulton, A. R. Prave, K.-H. Hoffmann, M. O. Clarkson, R. Guilbaud, J. W. Lyne, R. Tostevin, F. Bowyer, A. M. Penny, A. Curtis, S. A. Kasemann, Dynamic redox conditions control late Ediacaran metazoan ecosystems in the Nama Group Namibia. *Precambrian Res.* **261**, 252–271 (2015).

23. D. H. Erwin, Developmental capacity and the early evolution of animals. *J. Geol. Soc. London* **178**, jgs2020-245 (2021).

24. P. C. J. Donoghue, Z. Yang, The evolution of methods for establishing evolutionary timescales. *Philos. Trans. R. Soc. B: Biol. Sci.* **371**, 20160020 (2016).

25. D. H. Erwin, The origin of animal body plans: A view from fossil evidence and the regulatory genome. *Development* **147**, dev182899 (2020).

26. J. R. Paterson, G. D. Edgecombe, M. S. Y. Lee, Trilobite evolutionary rates constrain the duration of the Cambrian explosion. *Proc. Natl. Acad. Sci. U.S.A.* **116**, 4394–4399 (2019).

27. F. T. Bowyer, A. Y. Zhuravlev, R. Wood, F. Zhao, S. S. Sukhov, R. D. Alexander, S. W. Poulton, M. Zhu, Implications of an integrated late Ediacaran to early Cambrian stratigraphy of the Siberian platform Russia. *Geol. Soc. Am. Bull.* **135**, 2428–2450 (2023).

28. S. S. Sukhov, T. V. Pegel, Yu. Ya. Shabanov*, Regional Stratigraphic Chart of the Cambrian Strata of the Siberian Platform: Decisions of the All-Russian Stratigraphic Meeting on the Development of Stratigraphic Charts of the Upper Precambrian and Palaeozoic of Siberia*. (Novosibirsk, Siberian Scientific-Research Institute of Geology, Geophysics and Mineral Resources, 2021).

29. V. V. Khomentovsky, L. N. Repina, *The Lower Cambrian of the Stratotype Section of Siberia*. (Nauka, 1965).

30. L. N. Repina, Z. V. Borodaevskaya, V. V. Ermak, in *Cambrian of Siberia and Middle Asia* (eds I. T. Zhuravleva, L. N. Repina). *Trans. Inst. Geol. Geophys. Siberian Branch USSR Acad. Sci.* **720**, 3–31 [in Russian] (Moscow, Nauka, 1988).

31. T. V. Pegel, Evolution of Trilobite biofacies in Cambrian basins of the Siberian Platform. *J. Paleo.* **74**, 1000–1019 (2000).

32. E. Bushuev, I. Goryaeva, V. Pereladov, New discoveries of the oldest trilobites Profallotaspis and Nevadella in the northeastern Siberian Platform Russia. *Bull. Geosci.* **89**, 347–364 (2014).

33. E. Landing, M. D. Schmitz, G. Geyer, R. B. Trayler, S. A. Bowring, Precise early Cambrian U–Pb zircon dates bracket the oldest trilobites and archaeocyaths in Moroccan West Gondwana. *Geol. Mag.* **158**, 219–238 (2021).

34. S. S. Sukhov, Yu. Ya. Shabanov, T. V. Pegel, S. V. Saraev, Y. F. Filippov, I. V. Korovnikov, V. M. Sundukov, A. B. Fedorov, A. I. Varlamov, A. S. Efimov, V. A. Kontorovich, A. E. Kontorovich, *Stratigraphy of Oil and Gas Basins of Siberia. Cambrian of Siberian Platform, V. 1: Stratigraphy.* (Novosibirsk, Institute of Petroleum Geology and Geophysics, Siberian Branch, Russian Academy of Sciences, Novosibirsk, 2016).

35. M. D. Brasier, A. Y. Rozanov, A. Y. Zhuravlev, R. M. Corfield, L. A. Derry, A carbon isotope reference scale for the Lower Cambrian succession in Siberia: Report of IGCP Project 303. *Geol. Mag.* **131**, 767–783 (1994).

36. M. O. Clarkson, S. W. Poulton, R. Guilbaud, R. A. Wood, Assessing the utility of Fe/Al and Fe-speciation to record water column redox conditions in carbonate-rich sediments. *Chem. Geol.* **382**, 111–122 (2014).

37. S. W. Poulton, D. E. Canfield, Ferruginous conditions: A dominant feature of the ocean through Earth's history. *Elements* **7**, 107–112 (2011).

38. S. W. Poulton, *The Iron Speciation Paleoredox Proxy. Elements in Geochemical Tracers in Earth System Science*, (Cambridge Univ. Press, 2021).

39. C. Krewer, S. W. Poulton, R. J. Newton, C. März, B. J. W. Mills, T. Wagner, Controls on the termination of cretaceous oceanic anoxic event 2 in the Tarfaya Basin, Morocco. *Am. J. Sci.* **324**, 11 (2024).

40. N. Tribovillard, T. J. Algeo, F. Baudin, A. Riboulleau, Analysis of marine environmental conditions based onmolybdenum–uranium covariation—Applications to Mesozoic paleoceanography. *Chem. Geol.* **324-325**, 46–58 (2012).

41. D. B. Cole, D. B. Mills, D. H. Erwin, E. A. Sperling, S. M. Porter, C. T. Reinhard, N. J. Planavsky, On the co-evolution of surface oxygen levels and animals. *Geobiology* **18**, 260–281 (2020).

42. J. L. Morford, S. R. Emerson, E. J. Breckel, S. H. Kim, Diagenesis of oxyanions (V, U, Re, and Mo) in pore waters and sediments from a continental margin. *Geochim. Cosmochim. Acta* **69**, 5021–5032 (2005).

43. N. Glock, V. Liebetrau, A. Eisenhauer, I/Ca ratios in benthic foraminifera from the Peruvian oxygen minimum zone: Analytical methodology and evaluation as a proxy for redox conditions. *Biogeosciences* **11**, 7077–7095 (2014).

44. Z. Lu, B. A. A. Hoogakker, C.-D. Hillenbrand, X. Zhou, E. Thomas, K. M. Gutchess, W. Lu, L. Jones, R. E. M. Rickaby, Oxygen depletion recorded in upper waters of the glacial Southern Ocean. *Nat. Commun.* **7**, 11146 (2016).

45. Z. Lu, H. C. Jenkyns, R. E. M. Rickaby, Iodine to calcium ratios in marine carbonate as a paleo-redox proxy during oceanic anoxic events. *Geology* **38**, 1107–1110 (2010).

46. K. Huang, M. Cheng, T. J. Algeo, J. Hu, H. Wang, Z. Zhang, M. S. Dodd, Y. Wu, W. Guo, C. Li, Interaction of Shibantan Biota and environment in the terminal Ediacaran ocean: Evidence from I/(Ca+Mg) and sulfur isotopes. *Precambrian Res.* **379**, 106814 (2022).

47. A. S. Merdith, S. E. Williams, A. S. Collins, M. G. Tetley, J. A. Mulder, M. L. Blades, A. Young, S. E. Armistead, J. Cannon, S. Zahirovic, R. D. Müller, Extending full-plate tectonic models into deep time: Linking the Neoproterozoic and the Phanerozoic. *Earth Sci. Rev.* **214**, 103477 (2021).

48. T. W. Wong Hearing, A. Pohl, M. Williams, Y. Donnadieu, T. H. P. Harvey, C. R. Scotese, P. Sepulchre, A. Franc, T. R. A. Vandenbroucke, Quantitative comparison of geological data and model simulations constrains early Cambrian geography and climate. *Nat. Commun.* **12**, 3868 (2021).

49. V. A. Atashkin, T. V. Pegel, L. N. Repina, A. Y. Rozanov, Y. Y. Shabanov, A. Y. Zhuravlev, S. S. Sukhov, V. M. Sundukov, The Cambrian system on the Siberian platform: Correlation chart and explanatory notes. *Internat. Un. Geol. Sci. Publ.* **27**, 1–133 (1991).

50. T. H. Torsvik, L. R. M. Cocks, Gondwana from top to base in space and time. *Gondw. Res.* **24**, 999–1030 (2013).

51. G. W. O'Brien, A. R. Milnes, H. H. Veeh, D. T. Heggie, S. R. Riggs, D. J. Cullen, J. F. Marshall, P. J. Cook, Sedimentation dynamics and redox iron-cycling: Controlling factors for the apatite—Glauconite association on the East Australian continental margin. *Geol. Soc. Spec. Publ.* **52**, 61–86 (1990).

52. J.-G. Bréhéret, Glauconitization episodes in marginal settings as echoes of mid-Cretaceous anoxic events in the Vocontian basin (SE France). *Geol. Soc. Spec. Publ.* **58**, 415–425 (1991).

53. J. R. Creveling, D. T. Johnston, S. W. Poulton, B. Kotrc, C. März, D. P. Schrag, A. H. Knoll, Phosphorus sources for phosphatic Cambrian carbonates. *GSA Bull.* **126**, 145–163 (2014).

54. K. B. Föllmi, Sedimentary condensation. *Earth Sci. Rev.* **152**, 143–180 (2016).

55. R. L. Freeman, B. F. Dattilo, C. E. Brett, An integrated stratinomic model for the genesis and concentration of "small shelly fossil"-style phosphatic microsteinkerns in not-so-exceptional conditions. *Palaeogeogr. Palaeoclimatol. Palaeoecol.* **535**, 109344 (2019).

56. S. B. Pruss, B. C. Gill, Life on the edge: The Cambrian marine realm and oxygenation. *Annu. Rev. Earth Planet. Sci.* **52**, 109–132 (2024).

57. S. E. Peters, R. R. Gaines, Formation of the 'Great Unconformity' as a trigger for the Cambrian explosion. *Nature* **484**, 363–366 (2012).

58. V. A. Luchinina, I. V. Korovnikov, N. V. Novozhilova, D. A. Tokarev, Benthic Cambrian biofacies of the Siberian Platform (hyoliths, small shelly fossils, archeocyaths, trilobites and calcareous algae). *Stratigr. Geol. Correl.* **21**, 131–149 (2013).

59. A. Y. Zhuravlev, R. A. Wood, The two phases of the Cambrian explosion. *Sci. Rep.* **8**, 16656 (2018).

60. E. A. Sperling, A. H. Knoll, P. R. Girguis, The ecological physiology of Earth's second oxygen revolution. *Ann. Rev. Ecol. Evol. Syst.* **46**, 215–235 (2015).

61. M. J. Pushie, B. R. Pratt, T. C. MacDonald, G. N. George, I. J. Pickering, Evidence for biogenic copper (hemocyanin) in the middle Cambrian arthropod Marrella from the Burgess Shale. *PALAIOS* **29**, 512–524 (2014).

62. J.-B. Hou, N. C. Hughes, M. J. Hopkins, The trilobite upper limb branch is a well-developed gill. *Sci. Adv.* **7**, eabe7377 (2021).

63. J. B. Graham, Ecological, evolutionary, and physical factors influencing aquatic animal respiration. *Am. Zool.* **30**, 137–146 (1990).

64. H. B. Whittington,, B. D. E. Chatterton, S. E. Speyer, R. A. Fortey, R. M. Owens, W. T. Chang, W. T. Dean, P. A. Jell, J. R. Laurie, A. R. Palmer, L. N. Repina, A. W. A. Rushton, J. H. Shergold, E. N. K. Clarkson, N. V. Wilmot, S. R. A. Kelly, Part O, revised. Trilobita (Introduction, Order Agnostida, Order Redlichiida) *Treatise on Invertebrate Paleontology* (Geological Society of America and University of Kansas, 1997), p. 530.

65. D. B. Mills, L. M. Ward, C. Jones, B. Sweeten, M. Forth, A. H. Treusch, D. E. Canfield, Oxygen requirements of the earliest animals. *Proc. Natl. Acad. Sci. U.S.A.* **111**, 4168–4172 (2014).

66. V. Micaroni, F. Strano, R. McAllen, L. Woods, J. Turner, L. Harman, J. J. Bell, Adaptive strategies of sponges to deoxygenated oceans. *Glob. Change Biol.* **28**, 1972–1989 (2022).

67. J. V. Bannister, The respiration in air and in water of the limpets *Patella caerulea* (L.) and *Patella lusitanica* (Gmelin). *Comp. Biochem. Physical.* **49**, 407–411 (1974).

68. S. Calderon Lievanos, "Respuesta Bioenergética, Actividad Bioquímica Y Expresión Proteómica A Corto Y Largo Plazo, En Abulón Azul (*Haliotis Fulgens*, Philippi 1845) Expuesto A Cambios Repentinos De Estrés Térmico Y De Hipoxia," thesis, Centro de Investigaciones Biológicas del Noroeste, S.C. (2019).

69. H. Szaniawski, New evidence for the protoconodont origin of chaetognaths. *Acta Palaeontol. Pol.* **47**, 405–419 (2002).

70. D. Shu, S. Conway Morris, J. Han, J. F. Hoyal Cuthill, Z. Zhang, M. Cheng, H. Huang, Multi-jawed chaetognaths from the Chengjiang Lagerstätte (Cambrian, series 2, stage 3) of Yunnan, China. *Palaeontology* **60**, 763–772 (2017).

71. D. Pauly, C. Liang, W. Xian, E. Chu, N. Bailly, The sizes, growth and reproduction of arrow worms (Chaetognatha) in light of the gill-oxygen limitation theory (GOLT). *J. Mar. Sci. Eng.* **9**, 1397 (2021).

72. A. K. Val'kov, Biostratigraphy of the Lower Cambrian in the East of the Siberian Platform (Uchur-Maya Region). (Nauka, 1982).

73. A. K. Val'kov, Biostratigraphy of the Lower Cambrian in the East of the Siberian Platform (Yudoma-Olenek Region). (Nauka, 1987).

74. A. Kouchinsky, S. Bengtson, W. Feng, R. Kutygin, A. Val'kov, The lower cambrian fossil anabaritids: Affinities, occurrences and systematics. *J. Syst. Palaeontol.* **7**, 241–298 (2009).

75. A. Kouchinsky, R. Alexander, S. Bengtson, F. Bowyer, S. Clausen, L. E. Holmer, K. A. Kolesnikov, I. V. Korovnikov, V. E. Pavlov, C. B. Skovsted, G. Ushatinskaya, R. Wood, A. Y. Zhuravlev, Early–Middle Cambrian stratigraphy and faunas from northern Siberia. *Acta Palaeontol. Pol.* **67**, 341–464 (2022).

76. P. M. Novack-Gottshall, Ecosystem-wide body-size trends in Cambrian–Devonian marine invertebrate lineages. *Paleobiology* **34**, 210–228 (2008).

77. J. L. Payne, A. G. Boyer, J. H. Brown, S. Finnegan, M. Kowalewski, R. A. Krause Jr., S. K. Lyons, C. R. McClain, D. W. McShea, P. M. Novack-Gottshall, F. A. Smith, J. A. Stempien, S. C. Wang, Two-phase increase in the maximum size of life over 3.5 billion years reflects biological innovation and environmental opportunity. *Proc. Natl. Acad. Sci. U.S.A.* **106**, 24–27 (2009).

78. F. T. Bowyer, A. Y. Zhuravlev, R. Wood, G. A. Shields, Y. Zhou, A. Curtis, S. W. Poulton, D. J. Condon, C. Yang, M. Zhu, Calibrating the temporal and spatial dynamics of the Ediacaran - Cambrian radiation of animals. *Earth Sci. Rev.* **225**, 103913 (2022).

79. S. W. Poulton, D. E. Canfield, Development of a sequential extraction procedure for iron: Implications for iron partitioning in continentally derived particulates. *Chem. Geol.* **214**, 209–221 (2005).

80. L. J. Alcott, A. J. Krause, E. U. Hammarlund, C. J. Bjerrum, F. Scholz, Y. Xiong, A. J. Hobson, L. Neve, B. J. W. Mills, C. März, B. Schnetger, A. Bekker, S. W. Poulton,

Development of iron speciation reference materials for Palaeoredox analysis. *Geostand. Geoanal. Res.* **44**, 581–591 (2020).

81. T. He, R. J. Newton, P. B. Wignall, S. Reid, J. Dal Corso, S. Takahashi, H. Wu, S. Todaro, P. Di Stefano, V. Randazzo, M. Rigo, A. M. Dunhill, Shallow ocean oxygen decline during the end-Triassic mass extinction. *Glob. Planet. Change* **210**, 103770 (2022).

82. T. J. Algeo, N. Tribovillard, Environmental analysis of paleoceanographic systems based on molybdenum–Uranium covariation. *Chem. Geol.* **268**, 211–225 (2009).

83. R. M. Gaschnig, R. L. Rudnick, W. F. McDonough, A. J. Kaufman, J. W. Valley, Z. Hu, S. Gao, M. L. Beck, Compositional evolution of the upper continental crust through time, as constrained by ancient glacial diamictites. *Geochim. Cosmochim. Acta* **186**, 316–343 (2016).

84. K. Hans Wedepohl, The composition of the continental crust. *Geochim. Cosmochim. Acta* **59**, 1217–1232 (1995).

85. N. Tribovillard, T. J. Algeo, T. Lyons, A. Riboulleau, Trace metals as paleoredox and paleoproductivity proxies: An update. *Chem. Geol.* **232**, 12–32 (2006).

86. R. Raiswell, D. S. Hardisty, T. W. Lyons, D. E. Canfield, J. D. Owens, N. J. Planavsky, S. W. Poulton, C. T. Reinhard, The iron paleoredox proxies: A guide to the pitfalls, problems and proper practice. *Am. J. Sci.* **318**, 491–526 (2018).

87. S. W. Poulton, R. Raiswell, The low-temperature geochemical cycle of iron: From continental fluxes to marine sediment deposition. *Am. J. Sci.* **302**, 774–805 (2002).

88. S. W. Poulton, M. D. Krom, R. Raiswell, A revised scheme for the reactivity of iron (oxyhydr)oxide minerals towards dissolved sulfide. *Geochim. Cosmochim. Acta* **68**, 3703–3715 (2004).

89. S. E. Calvert, T. F. Pedersen, Geochemistry of recent oxic and anoxic marine sediments: Implications for the geological record. *Mar. Geol.* **113**, 67–88 (1993).

90. J. Crusius, S. Calvert, T. Pedersen, D. Sage, Rhenium and molybdenum enrichments in sediments as indicators of oxic, suboxic and sulfidic conditions of deposition. *Earth Planet. Sci. Lett.* **145**, 65–78 (1996).

91. R. F. Anderson, M. Q. Fleisher, A. P. LeHuray, Concentration, oxidation state, and particulate flux of uranium in the Black Sea. *Geochim. Cosmochim. Acta* **53**, 2215–2224 (1989).

92. G. R. Helz, C. V. Miller, J. M. Charnock, J. F. W. Mosselmans, R. A. D. Pattrick, C. D. Garner, D. J. Vaughan, Mechanism of molybdenum removal from the sea and its concentration in black shales: EXAFS evidence. *Geochim. Cosmochim. Acta* **60**, 3631–3642 (1996).

93. G. T. F. Wong, P. G. Brewer, The marine chemistry of iodine in anoxic basins. *Geochim. Cosmochim. Acta* **41**, 151–159 (1977).

94. W. Lu, A. J. Dickson, E. Thomas, R. E. M. Rickaby, P. Chapman, Z. Lu, Refining the planktic foraminiferal I/Ca proxy: Results from the Southeast Atlantic Ocean. *Geochim. Cosmochim. Acta* **287**, 318–327 (2020).

95. D. S. Hardisty, Z. Lu, N. J. Planavsky, A. Bekker, P. Philippot, X. Zhou, T. W. Lyons, An iodine record of Paleoproterozoic surface ocean oxygenation. *Geology* **42**, 619–622 (2014).

96. D. S. Hardisty, Z. Lu, A. Bekker, C. W. Diamond, B. C. Gill, G. Jiang, L. C. Kah, A. H. Knoll, S. J. Loyd, M. R. Osburn, N. J. Planavsky, C. Wang, X. Zhou, T. W. Lyons, Perspectives on Proterozoic surface ocean redox from iodine contents in ancient and recent carbonate. *Earth Planet. Sci. Lett.* **463**, 159–170 (2017).

97. W. Lu, S. Wörndle, G. P. Halverson, X. Zhou, A. Bekker, R. H. Rainbird, D. S. Hardisty, T. W. Lyons, Z. Lu, Iodine proxy evidence for increased ocean oxygenation during the Bitter Springs Anomaly. *Geochem. Persp. Let.* **53–57**, 53–57 (2017).

98. R. He, A. Pohl, A. Prow, G. Jiang, C. C. Huan, M. R. Saltzman, Z. Lu, The dynamic ocean redox evolution during the late Cambrian SPICE: Evidence from the I/Ca proxy. *Global Planet. Change* **233**, 104354 (2024).

99. K. V. Lau, D. S. Hardisty, Modeling the impacts of diagenesis on carbonate paleoredox proxies. *Geochim. Cosmochim. Acta* **337**, 123–139 (2022).

100. M. Zhu, A. Y. Zhuravlev, R. A. Wood, F. Zhao, S. S. Sukhov, A deep root for the Cambrian explosion: Implications of new bio- and chemostratigraphy from the Siberian Platform. *Geology* **45**, 459–462 (2017).

101. Yu. L. Pel'man, N. A. Aksarina, S. P. Koneva, L. E. Popov, L. P. Sobolev, G. T. Ushatinskaya, *The oldest brachiopods from the territory of northern Eurasia*, (Novosibirsk, OIGGiM SO RAN., 1992), p. 145 [in Russian].

102. V. A. Datsenko, L. T. Zhuravleva, N. P. Lazarenko, Yu. N. Popov, N. E. Chernysheva, Biostratigraphy and fauna of the Cambrian deposits of the northwestern Siberian Platform. (Nauchno-Issledovatel'skiy Institut Geologii Arktiki, Trudy 155, 1968), pp. 1–213 [in Russian].

103. V. V. Khomentovsky, G. A. Karlova, in *Late Precambrian and Early Palaeozoic of Siberia. Actual Problems of the Stratigraphy* (eds V. V. Khomentovsky, Yu. K. Sovetov) 23–61 [in Russian] (Institute of Geology and Geophysics, Siberian Branch, USSR Academy of Sciences, 1989).

104. V. V. Khomentovsky, G. A. Karlova, in *Late Precambrian and Early Palaeozoic of Siberia. Siberian Platform and Its Borderland* [in Russian], V. V. Khomentovsky (United Institute of Geology, Geophysics and Mineralogy, Siberian Branch, USSR Academy of Sciences, 1991), p. 3–44.

105. K. E. Nagovitsin, V. I. Rogov, V. V. Marusin, G. A. Karlova, A. V. Kolesnikov, N. V. Bykova, D. V. Grazhdankin, Revised Neoproterozoic and Terreneuvian stratigraphy of the Lena-Anabar Basin and north-western slope of the Olenek Uplift, Siberian Platform. *Precambrian Res.* **270**, 226–245 (2015).

106. A. Kouchinsky, S. Bengtson, E. Landing, M. Steiner, M. Vendrasco, K. Ziegler, Terreneuvian stratigraphy and faunas from the Anabar Uplift, Siberia. *Acta Palaeontol. Pol.* **62**, 311–440 (2017).

107. A. K. Valkov, *Biostratigraphy and Hyoliths of the Cambrian of Northeastern Siberian Platform*, (Moscow, Nauka, 1975), p. 139.

108. V. V. Khomentovsky, A. B. Fedorov, G. A. Karlova, Lower Cambrian boundary in the inner areas on the North Siberian Platform. *Stratigr. Geol. Correl.* **6**, 3–9 (1998).

109. A. Yu. Rozanov, V. V. Missarzhevsky, N. A. Volkova, L. G. Voronova, I. N. Krylov, B. M. Keller, I. K. Korolyuk, K. Lendzion, R. Michniak, N. G. Pykhova, A. D. Sidorov, *The Tommotian Stage and the Cambrian Lower Boundary Problem* (English translation, Amerind Publishing Co., 1981; Nauka, 1969).

110. S. M. Rowland, V. A. Luchinina, I. V. Korovnikov, D. P. Sipin, A. I. Tarletskov, A. V. Fedoseev, Biostratigraphy of the Vendian-Cambrian Sukharikha River section, northwestern Siberian Platform. *Can. J. Earth Sci.* **35**, 339–352 (1998).

111. V. V. Khomentovsky, G. A. Karlova, The boundary between Nemakit-Daldynian and Tommotian stages (Vendian-Cambrian Systems) of Siberia. *Stratigr. Geol. Correl.* **10**, 13–34 (2002).

112. A. I. Varlamov, A. Yu. Rozanov, V. V. Khomentovsky, Yu. Ya. Shabanov, G. P. Abaimova, Yu. E. Demidenko, G. A. Karlova, I. V. Korovnikov, V. A. Luchinina, Ya. E. Malakhovskaya, P. Yu. Parkhaev, T. V. Pegel, N. A. Skorlotova, V. M. Sundukov, S. S. Sukhov, A. B. Fedorov, L. K. Kipriyanova, *The Cambrian System of the Siberian Platform. Part 1: The Aldan-Lena Region*. (Moscow; Novosibirsk, PIN RAS. 2008) p. 300.

113. V. V. Khomentovsky, G. A. Karlova, The boundary between Nemakit-Daldynian and Tommotian stages (Vendian-Cambrian) of Siberia. *Stratigr. Geol. Correl.* **10**, 217–238 (2002).

114. V. I. Korshunov, L. N. Repina, V. A. Sysoev, To the structure of the Pestrotsvet Formation on the East of the Aldan Anteclise. (*Geol. Geofiz.*, 10, 1969), pp. 18–21 [in Russian].

115. A. K. Val'kov, Distribution of the oldest skeletal organisms and correlation of the Cambrian lower boundary in southeastern part of the Siberian Platform in *Late Precambrian and Early*

*Palaeozoic of Siberia. Vendian Strata*, V. V. Khomentovsky, ed. (United Institute of Geology, Geophysics and Mineralogy, Siberian Branch, USSR Academy of Sciences, Novosibirsk, 1983), pp. 37–48. [in Russian].

116. A. Yu. Rozanov, P. Yu. Parkhaev, Yu. E. Demidenko, G. A. Karlova, I. V. Korovnikov, Yu. Ya. Shabanov, A. Yu. Ivantsov, V. A. Luchinina, Ya. E. Malakhovskaya, L. M. Melnikova, E. B. Naimark, A. G. Ponomarenko, N. A. Skorlotova, V. M. Sundukov, D. A. Tokarev, G. T. Ushatinskaya, L. K. Kipriyanova, *Fossils from the Lower Cambrian Stage Stratotypes*, (Moscow, PIN RAN., 2010), p. 228 [in Russian].

117. L. Devaere, S. Clausen, M. Steiner, J.-J. Álvaro, D. Vachard, Chronostratigraphic and palaeogeographic significance of an early Cambrian microfauna from the Heraultia Limestone, northern Montagne Noire, France. *Palaeontol. Electronica* **16**, 91 (2013).

118. P. Y. Parkhaev, Y. E. Demidenko, M. A. Kulsha, The problematic fossil *Mobergella radiolata* as an index species of the Lower Cambrian stages. *Stratigr. Geol. Correl.* **28**, 135–156 (2020).

119. P. Y. Parkhaev, Y. E. Demidenko, Zooproblematica and mollusca from the Lower Cambrian Meishucun section (Yunnan, China) and taxonomy and systematics of the Cambrian small shelly fossils of China. *Paleontol. J.* **44**, 883–1161 (2010).

120. P. Y. Parkhaev, G. A. Karlova, Taxonomic revision and evolution of Cambrian mollusks of the genus *Aldanella* Vostokova, 1962 (Gastropoda: Archaeobranchia). *Paleontol. J.* **45**, 1145–1205 (2011).

121. P. Y. Parkhaev, Two new species of the Cambrian helcionelloid mollusks from the northern part of the Siberian Platform. *Paleontol. J.* **39**, 615–619 (2005).

122. A. P. Gubanov, J. S. Peel, The early Cambrian helcionelloid mollusc *Anabarella* Vostokova. *Palaeontology* **46**, 1073–1087 (2003).

123. V. V. Khomentovsky, G. A. Karlova, On the lower boundary of the Pestrotsvet Formation in the Aldan River basin in *Late Precambrian and Early Palaeozoic of Siberia. Siberian*

*Platform and the Outer Zone of the Altay-Sayan Foldbelt*, V. V. Khomentovsky, ed. (United Institute of Geology, Geophysics and Mineralogy, Siberian Branch, USSR Academy of Sciences, Novosibirsk 1986), pp. 3–22 [in Russian].

124. V. V. Khomentovsky, G. A. Karlova, Specifics of the ecology of Vendian-Cambrian small shelly fossil biota from Siberian Platform. *Stratigr. Geol. Correl.* **2**, 8–17 (1994).

125. G. Geyer, The Moroccan fallotaspidid trilobites revisited. *Ther. Ber.* **18**, 89–199 (1996).

126. G. Geyer, The earliest known West Gondwana trilobites from the Anti-Atlas of Morocco, with a revision of the Family Bigotinidae Hupé, 1953. *Foss. Strata* **64**, 55–153 (2019).

127. L. N. Repina, Evolution of trilobites at the early stages of their historical development. *Trans. Inst. Geol. Geophys. Siberian Branch USSR Acad. Sci.* **764**, 34–44 (1990).

128. L. N. Repina, Dependence of morphologic features on habitat conditions in trilobites and evaluation of their significance for the systematics of the superfamily Olenelloidea in *Environment and Life in the Geological Past (Problems of Ecostratigraphy)*, O. A. Betekhtina, I. T. Zhuravleva, eds. (Novosibirsk, Nauka, 1979), pp. 11–30 [in Russian].

129. N. P. Meshkova, Lower Cambrian hyoliths of the Siberian Platform. *Trans. Inst. Geol. Geophys. Siberian Branch USSR Acad. Sci.* **1**, 110 (1974). .

130. I. N. Krasilova, Fordillids (Bivalvia) from the Lower Cambrian of the Siberian Platform. *Paleontologicheskiy Zhurnal* **1977**, 42–48 (1977).

131. V. A. Astashkin, A. K. Valkov, L. G. Voronova, N. V. Grigorieva, L. I. Egorova, E. A. Zhegallo, A. Yu. Zhuravlev, I. T. Zhuravleva, V. I. Korshunov, V. A. Luchinina, V. V. Missarzhevsky, L. M. Melnikova, N. P. Meshkova, D. V. Osadchaya, Yu. L. Pelman, L. N. Repina, A. Yu. Rozanov, V. M. Sundukov, N. P. Suvorova, V. A. Sysoev, V. D. Fonin, *Early Cambrian Stage Subdivision of Siberia. Atlas of Fossils*. (Institute of Geology and Geophysics, Siberian Branch, USSR Academy of Sciences, Transactions 558. Nauka, Moscow, 1983), p. 216 [in Russian].

132. V. V. Khomentovsky, A. K. Valkov, G. A. Karlova S. V. Nuzhnov, Key section of Precambrian Cambrian deposits of the Gonam River in Late Precambrian and Lower Palaeozoic of Siberia. Vendian Deposits (Novosibirsk, 1983), pp. 29–44.

133. Yu. L. Pel'man, V. V. Ermak, A. B. Fedorov, V. A. Luchinina, I. T. Zhuravleva, L. N. Repina, V. I. Bondarev, Z. V. Borodaevskaya, [New data on the upper Precambrian and lower Cambrian stratigraphy and palaeontology of the r. Dzhanda (right tributary of the r. Aldan)] in Biostratigraphy and Paleontology of the Cambrian of Northern Asia, L. N. Repina, ed. (Institut Geologii i Geofiziki Sibirskogo Otdeleniya Akademii Nauk SSSR, Trudy 765, 1990), pp. 3–43 1990 [in Russian].

134. V. A. Datsenko, I. T. Zhuravleva, N. P. Lazarenko, Yu. N. Popov, N. E. Chernysheva, Biostratigraphy and fauna of the Cambrian deposits of the northwestern Siberian Platform. Trans. Sci.-Res. Inst. Geol. Arctic 155 [in Russian] (Nedra, 1968).

135. I. T. Zhuravleva, Yu. K. Sovetov, T. Titorenko, N. in *Stratigraphy of the Lower Cambrian and Upper Precambrian in the South of the Siberian Platform*, B. S. Sokolov [in Russian] (Nauka, 1969b), pp. 13–16.

136. Z. A. Akul'cheva, E. M. Galperova, E. L. Drobkova, L. A. Lysova, T. N. Titorenko, A. A. Treshchetenkova, Z. Kh. Fayzulina, Mota strata and their analogies in the Irkutsk Amphitheater in *Boundary Strata of the Precambrian and Cambrian of the Siberian Platform (Biostratigraphy, Palaeontology, Conditions of the Formation)*, N. P. Meshkova, I. V. Nikolaeva, eds. (Institut Geologii i Geofiziki Sibirskogo Otdeleniya Akademii Nauk SSSR, Trudy 475 Novosibirsk, Nauka, 1981), pp. 65–139. [in Russian].

137. T. I. Burtseva, I. T. Zhuravleva, First find of archaeocyaths in the Irkutsk Amphitheatre. Dokl. Akad. Nauk SSSR 106, 885–888 [in Russian] (1956).

138. I. N. Dyatlova, A. N. Deonov, V. R. Trofimov in *Biostratigraphy and Palaeontology of the Cambrian of Northern Asia*, L. N. Repina, ed. *Trans. Inst. Geol. Geophys. Siberian Branch USSR Acad. Sci.* 765, Nauka, 1990), pp. 123–135 [in Russian].

139. V. V. Gritsik, in *Problems of Lower Cambrian Palaeontology and Biostratigraphy of Siberia and the Far East*, I. T. Zhuravleva, ed. (Nauka, 1969), pp. 186–202 [in Russian].

140. A. Kouchinsky, S. Bengtson, S. Clausen, M. J. Vendrasco, An early Cambrian fauna of skeletal fossils from the Emyaksin Formation, northern Siberia. *Acta Palaeontol. Pol.* **60**, 421–512 (2015).

141. N. P. Meshkova, I. V. Nikolaeva, Y. P. Kulikov, I. T. Zhuravleva, V. A. Luchinina, D. I. Musatov, S. D. Sidoras, Stratigraphy of Precambrian-Cambrian boundary strata on the North of the Anabar Uplift, (ed. Zhuravleva, I.T., Lower and Middle Cambrian stratigraphy and palaeontology of the U.S.S.R.). Trans. Inst. Geol. Geophys. Siberian Branch USSR Acad. Sci, 296, 3–22 (1976).

142. L. I. Egorova, V. E. Savistkiy, *Stratigraphy and Biofacies of the Cambrian of the Siberian Platform (Western Anabar Area).* (*Trans. Sib. Sci.-Res. Inst. Geol. Geophys. Miner. Res.*) **43** [in Russian] (Nedra, 1969).

143. V. M. Sundukov, F. B. Fedorov, in *Biostratigraphy and Palaeontology of the Cambrian of Northern Asia*, I. T. Zhuravleva, ed. (*Trans. Inst. Geol. Geophys. Siberian Branch USSR Acad. Sci.* 669, Nauka, 1986), pp. 108–119 [in Russian].

144. B. B. Shishkin, Vendian deposits of the southestern part of Siberian Platform. *Geol. Miner. Resour. Sib.* **3**, 3–10 (2011).

145. V. I. Korshunov, *Lower Cambrian Biostratigraphy and Archaeocyaths of the Northeastern Aldan Anteclise* (Yakutsk Publishers, 1972) [in Russian].

146. A. I. Goryachev, M. A. Zharkov, in *Stratigraphy of the Lower Cambrian and Upper Precambrian in the South of the Siberian Platform*, B. S. Sokolov, ed., (Nauka, 1969), pp. 17–33 [in Russian].

147. V. V. Marusin, A. A. Kolesnikova, B. B. Kochnev, N. B. Kuznetsov, B. G. Pokrovsky, T. V. Romanyuk, G. A. Karlova, S. V. Rud'ko, A. V. Shatsillo, A. S. Dubenskiy, V. S. Sheshukov, S. M. Lyapunov, Detrital zircon age and biostratigraphic and

chemostratigraphic constraints on the Ediacaran–Cambrian transitional interval in the Cis-Sayans Uplift, southwestern Siberian Platform. *Geol. Mag.* **158**, 1156–1172 (2021).

148. M. A. Zharkov, Yu. K. Sovetov, in *Stratigraphy of the Lower Cambrian and Upper Precambrian in the South of the Siberian Platform*, B. S. Sokolov, ed., (Nauka, 1969), pp. 34–55 [in Russian].

149. V. M. Sundukov, New archaeocyaths from the Lower Cambrian of the Lena and Kotuy. *Paleontol. Zh.* **1983**, 13–17 (1983).

150. I. T. Zhuravleva in *Biostratigraphy and Palaeontology of the Lower and Middle Cambrian of Northern Asia*, N. P. Meshkova, ed. (*Trans. Inst. Geol. Geophys. Siberian Branch USSR Acad. Sci.* 541, Nauka, 1983), pp. 81–94 [in Russian].

151. L. N. Repina, V. A. Luchinina, To the biostratigraphy of the lower part of the Lower Cambrian in northwestern Prinabar'e (R. Fomich) In N. P. Meshkova, I. V. Nikolaeva, eds. (Boundary Strata of the Precambrian and Cambrian of the Siberian Platform (Biostratigraphy, Palaeontology, Conditions of the Formation). Institut Geologii i Geofiziki Sibirskogo Otdeleniya Akademii Nauk SSSR, Trudy 475, 3–19. Novosibirsk, Nauka, 1981 [in Russian].

152. V. E. Savitskiy, New data on the stratigraphy of the Cambrian strata on the Anabar anteclise. *Collective Papers on the Geology of the Arctic* (1959), vol. 102 [in Russian].

153. N. P. Meshkova, I. T. Zhuravleva, V. A. Luchinina, Lower Cambrian and the lower part of the Middle Cambrian of the Olenek Uplift in *Problems of Paleontology and Biostratigraphy of the Lower Cambrian of Siberia and the Far* East, I. T. Zhuravleva, ed., (Novosibirsk, Nauka, 1973), pp. 194–214 [in Russian].

154. V. E. Savitskiy, Yu.Ya. Shabanov, B. B. Shishkin, Stratigraphy of the Precambrian and Cambrian strata of the Igarka area. Sibirskiy Institut Geologii, Geofiziki i Mineral'nogo Syr'ya, Trudy, Seriya Neftyanaya Geologiya Neftegazonosnykh Rayonov Sibiri, 32, Pt II 1964 (in Russian).

155. I. V. Korovnikov, Trilobites of the suborder Eodiscina from the Lower Cambrian of the northeastern Siberian Platform (Khorbosuonka River section). *Paleontol. J.* **41**, 614–620 (2007).

156. L. I. Egorova, L. N. Repina, N. P. Suvorova, Lower Cambrian stage subdivision of the Siberia. Atlas of fossils. Trans. Inst. Geol. *Geophys. Siberian Branch USSR Acad. Sci*. **558**, 1–216 (1983).

157. T. V. Pegel, L. I. Egorova, Yu. Ya. Shabanov, I. V. Korovnikov, V. A. Luchinina, A. K. Salikhova, V. M. Sundukov, A. B. Fedorov, A. Yu. Zhuravlev, P. Yu. Parkhaev, Yu. E. Demidenko, Stratigraphy of Oil and Gas Basins of Siberia. Cambrian of Siberian Platform. V. 2 – Palaeontology [in Russian] (Institute of Petroleum Geology and Geophysics, Siberian Branch, Russian Academy of Sciences, 2016).

158. A. I. Varlamov, L. I. Egorova, Stratigrafiya nizhnekembriyskikh otlozheniy t. Tol'by (yug Sibirskoy platformy) in *Biostratigrafiya i paleontologiya kembriya Severnoy Azii*, I. T. Zhuravleva, ed. (Trudy Akademiya Nauk SSSR, Sibirskoe otdelenie, Nauka, Novosibirsk, 1986), 669, 65–76.

159. L. I. Egorova, Novye nizhnekembriyskie trilobity yugo-vostoka Sibirskoy platformy. *Paleontologicheskiy Zhurnal* **1983**, 59–64 (1983).

160. L. N. Repina, V. A. Luchinin, To the biostratigraphy of the lower part of the Lower Cambrian in northwestern Prinabar'e (R. Fomich) in *Boundary Strata of the Precambrian and Cambrian of the Siberian Platform (Biostratigraphy, Palaeontology, Conditions of the Formation*, N. P. Meshkova, I. V. Nikolaeva, eds. (Institut Geologii i Geofiziki Sibirskogo Otdeleniya Akademii Nauk SSSR, Trudy 475. Novosibirsk, Nauka, 1981) pp. 3–19 [in Russian].

161. L. Na, W. Kiessling, Diversity partitioning during the Cambrian radiation. *Proc. Natl. Acad. Sci. U.S.A.* **112**, 4702–4706 (2015).

162. M. dos Reis, Y. Thawornwattana, K. Angelis, M. J. Telford, P. C. J. Donoghue, Z. Yang, Uncertainty in the timing of origin of animals and the limits of precision in molecular timescales. *Curr. Biol.* **25**, 2939–2950 (2015).

163. T. H. Torsvik, L. R. M. Cocks, The integration of palaeomagnetism, the geological record and mantle tomography in the location of ancient continents. *Geol. Mag.* **156**, 242–260 (2019).

164. L. N. Repina, N. P. Lazarenko, N. P. Meshkova, V. I. Korshunov, N. I. Nikiforov, N. A. Aksarina, Biostratigraphy and Fauna of the Lower Cambrian of the Kharaulakh (Tuora-Sis Ridge). Trans. Inst. Geol. Geophys. Siberian Branch USSR Acad. Sci. 235 [in Russian] (Nauka, 1974).

165. V. A. Luchinina, I. V. Korovnikov, D. P. Sipin, A. V. Fedoseev, Biostratigraphy of the Upper Vendian-Lower Cambrian in the Sukharikha River section (Siberian Platform). *Geol. Geophys.* **38**, 1346–1358 (1997).

166. A. Kouchinsky, S. Bengtson, V. Pavlov, B. Runnegar, P. Torssander, E. Young, K. Ziegler, Carbon isotope stratigraphy of the Precambrian–Cambrian Sukharikha River section, northwestern Siberian platform. *Geol. Mag.* **144**, 609–618 (2007).

167. A. Kouchinsky, S. Bengtson, V. Pavlov, B. Runnegar, A. Val'kov, E. Young, Pre-Tommotian age of the lower Pestrotsvet Formation in the Selinde section on the Siberian platform: Carbon isotopic evidence. *Geol. Mag.* **142**, 319–325 (2005).
